# Coexistence of *bla*_IMP-4_, *bla*_NDM-1_ and *bla*_OXA-1_ in *bla*_KPC-2_-producing *Citrobacter freundii* of clinical origin in China

**DOI:** 10.3389/fmicb.2023.1074612

**Published:** 2023-06-12

**Authors:** Jie Qiao, Yingying Chen, Haoyu Ge, Hao Xu, Xiaobing Guo, Ruishan Liu, Chenyu Li, Ruyan Chen, Jianjun Gou, Mantao Chen, Beiwen Zheng

**Affiliations:** ^1^State Key Laboratory for Diagnosis and Treatment of Infectious Diseases, The First Affiliated Hospital, College of Medicine, Zhejiang University, Hangzhou, China; ^2^Department of Laboratory Medicine, The First Affiliated Hospital of Zhengzhou University, Zhengzhou, China; ^3^Department of Neurosurgery, Shaoxing People’s Hospital (Shaoxing Hospital, Zhejiang University School of Medicine), Shaoxing, China; ^4^Department of Neurosurgery, the First Affiliated Hospital, College of Medicine, Zhejiang University, Hangzhou, China; ^5^Department of Structure and Morphology, Jinan Microecological Biomedicine Shandong Laboratory, Jinan, China; ^6^Research Units of Infectious Diseases and Microecology, Chinese Academy of Medical Sciences, Beijing, China

**Keywords:** *Citrobacter freundii*, In*1337*, Tn*AS3*, In*2054*, Tn*2*, *bla*_IMP-4_, *bla*_NDM-1_, *bla*_KPC-2_

## Abstract

**Purpose:**

To explore the genetic characteristics of the IMP-4, NDM-1, OXA-1, and KPC-2 co-producing multidrug-resistant (MDR) clinical isolate, *Citrobacter freundii* wang9.

**Methods:**

MALDI-TOF MS was used for species identification. PCR and Sanger sequencing analysis were used to identify resistance genes. In addition to agar dilution, broth microdilution was used for antimicrobial susceptibility testing (AST). We performed whole genome sequencing (WGS) of the strains and analyzed the resulting data for drug resistance genes and plasmids. Phylogenetic trees were constructed with maximum likelihood, plotted using MAGA X, and decorated by iTOL.

**Results:**

*Citrobacter freundii* carrying *bla*_KPC-2_, *bla*_IMP-4_, *bla*_OXA-1_, and *bla*_NDM-1_ are resistant to most antibiotics, intermediate to tigecycline, and only sensitive to polymyxin B, amikacin, and fosfomycin. The *bla*_IMP-4_ coexists with the *bla*_NDM-1_ and the *bla*_OXA-1_ on a novel transferable plasmid variant pwang9-1, located on the integron In*1337*, transposon Tn*AS3*, and integron In*2054*, respectively. The gene cassette sequence of integron In*1337* is *IntI1-bla*_IMP-4_*-qacG2-aacA4′-catB3Δ*, while the gene cassette sequence of In*2054* is *IntI1-aacA4cr-bla*_OXA-1_*-catB3-arr3-qacEΔ1-sul1.* The *bla*_NDM-1_ is located on the transposon Tn*AS3*, and its sequence is IS*91-sul-*IS*Aba14-aph (3′)-VI-*IS*30-bla*_NDM-1_*-ble-trpF-dsbD-*IS*91.* The *bla*_KPC-2_ is located on the transposon Tn*2* of plasmid pwang9-1, and its sequence is *klcA-korC-*IS*kpn6-bla*_KPC-2_*-*IS*kpn27-tnpR-tnpA.* Phylogenetic analysis showed that most of the 34\u00B0*C. freundii* isolates from China were divided into three clusters. Among them, wang1 and wang9 belong to the same cluster as two strains of *C. freundii* from environmental samples from Zhejiang.

**Conclusion:**

We found *C. freundii* carrying *bla*_IMP–4_, *bla*_NDM–1_, *bla*_OXA-1_, and *bla*_KPC-2_ for the first time, and conducted in-depth research on its drug resistance mechanism, molecular transfer mechanism and epidemiology. In particular, we found that *bla*_IMP-4_, *bla*_OXA-1_, and *bla*_NDM-1_ coexisted on a new transferable hybrid plasmid that carried many drug resistance genes and insertion sequences. The plasmid may capture more resistance genes, raising our concern about the emergence of new resistance strains.

## 1. Introduction

*Citrobacter* is a facultative anaerobic Gram-negative bacteria, which exists widely in nature, such as water, soil and food. Today *C. freundii* is regarded as an important nosocomial pathogen and is frequently found in patients’ blood, urine, soft tissues, and wounds (Khorasani et al., 2008; Kumar et al., 2013). In some extensive observational studies, *Citrobacter* accounted for approximately 3–6% of *Enterobacteriaceae* isolates in clinical settings (Mohanty et al., 2007). Carbapenems are the primary antimicrobial drugs for treating serious infections caused by ESBL-producing bacteria (Nicolau, 2008). The emergence of *C. freundii* carrying carbapenem-resistant gene (such as *bla*_KPC_, *bla*_NDM_, and *bla*_IMP_) has brought more significant challenges to clinical treatment (Hammerum et al., 2016; Xiong et al., 2016; Xu et al., 2018). Even the coexistence of multiple carbapenemase genes, such as *bla*_KPC-2_ + *bla*_NDM-1_, *bla*_NDM-1_ + *bla*_IMP-14_, *bla*_KPC-2_ + *bla*_NDM-1_ + *bla*_NDM-5_, undoubtedly makes antibiotic use less selective (Rimrang et al., 2012; Feng et al., 2015; Zheng et al., 2018). Carbapenemase-resistant *C. freundii* may cause an outbreak of nosocomial infection and seriously threaten public health (Pletz et al., 2018; Jung et al., 2020).

Carbapenemases can be divided into two groups according to their degree of cation dependence: serine carbapenemases (zinc-independent: classes A, C, and D) and metallo-beta-lactamases (MBLs; zinc-dependent: class B) (Queenan and Bush, 2007). Of the latter, VIM, IMP, and NDM types are the most prevalent types of carbapenemases globally (Nordmann, 2014).

IMP-type MBLs are the earliest transferable carbapenemases reported in Gram-negative bacteria. The *bla*_IMP-1_ was first discovered in *Pseudomonas aeruginosa* in Japan in 1991, and then quickly appeared in countries around the world (Watanabe et al., 1991). Over time, many variants of IMP have appeared, such as *bla*_IMP-8_, *bla*_IMP-26_, and have also been found in various *Enterobacteriaceae* bacteria, such as *Enterobacter hormaechei*, *Klebsiella pneumoniae* (Zheng et al., 2015; Gou et al., 2020; Guo et al., 2021). So far, 97 *bla*_IMP_ variants have been identified (November/2022).^1^ The *bla*_IMP-4_ has been the most reported IMP variant, frequently found in class 1 integrons and carried by multiple plasmid types (such as HI2, L/M, A/C, and N) for horizontal transfer (Roberts et al., 2020). However, unlike NDM-type MBLs, *bla*_IMP_ is not common in CREs from China (Han et al., 2020).

NDM-type MBLs were widespread worldwide, and 44 NDM-type variants have been identified (November/2022) (see Footnote 1). Among the 44 NDM-type variants, NDM-1 has the broadest host spectrum discovered so far, and has been found in many species of 11 bacterial families, of which *K. pneumoniae* and *Escherichia coli* being the main carriers of *bla*_NDM_ (Zheng et al., 2011; Wu et al., 2019). Most *bla*_NDM_ are located on plasmids, and most plasmids carrying *bla*_NDM_ belong to the limited replicon type (IncX3, IncFII, and IncC) (Kumarasamy et al., 2010; Baraniak et al., 2016). NDM-positive strains can cause various infections that have been reported to be associated with high mortality (Guducuoglu et al., 2018).

According to the SMART global surveillance program, KPC is now the most widespread carbapenemase in the world. The *bla*_KPC-2_ was first detected in *K. pneumoniae* in North Carolina in 2001, spreading rapidly around the world (Yigit et al., 2001; Zheng et al., 2020). Now, 144 KPC-type variants have been identified (November/2022).^3^

KPC is a serine enzyme that can be inhibited by β-lactamase inhibitors, such as avibactam, clavulanic acid, etc. MBLs degrades almost all beta-lactam antibiotics, and its activity cannot be suppressed by clinically available beta-lactamase inhibitors, including avibactam, relebactam and vaborbactam (Boyd et al., 2020). Moreover, strains carrying *bla*_NDM_ and *bla*_IMP_ were also resistant to ceftazidime/avibactam (Bush and Bradford, 2019). The clinical treatment options for pathogens that carry serine enzymes or metalloenzymes are quite different. However, once the same strain of metalloenzymes and serine enzymes coexist, the clinical treatment options will be more challenging to choose.

At present, the study of the multidrug resistance in *C. freundii* has been reported sporadically, especially few studies has been conducted on resistance plasmids that carry multiple carbapenemase genes (coexistence of *bla*_NDM-1_, *bla*_OXA-1_, and *bla*_IMP-4_). The research on the in the same transferable plasmid is not enough. We found a strain of *C. freundii* carrying *bla*_KPC-2_, *bla*_NDM-1_, *bla*_OXA-1_, and *bla*_IMP-4_ from cerebrospinal fluid, and through further research, we found that *bla*_NDM-1_, *bla*_OXA-1_, and *bla*_IMP-4_ coexist in a novel hybrid plasmid variant. We conducted in-depth research on the drug resistance mechanism, plasmid structure, horizontal transfer and epidemiology of *C. freundii*.

## 2. Materials and methods

### 2.1. Collection of bacterial strains and identification of antibiotic resistance genes

We continuously collected carbapenem-resistant Gram-negative bacilli from a tertiary hospital in Henan, China from 2018 to 2022. The antimicrobial susceptibility of the strains was preliminarily tested by VITEK^®^2 Compact (BioMerieux, Marcy l’Etoile, France), and then identified by MALDI-TOF MS (Bruker, Bremen, Germany) (Bizzini and Greub, 2010). We used PCR to identify common carbapenemase-encoding genes, such as *bla*_KPC_ (F: ATGTCACTGTATCGCCGTC; R: TTACTGCCCGTTGACGCC), *bla*_IMP_ (F: GTTTATGTTCATACWTCG; R: GGTTTAAYAAAACAACCAC), *bla*_NDM_ (F: ATGGAATTGCCCAATATTATGCAC; R: TCAGCGCAGCTTGTCGGC), and *bla*_OXA-48_ (F: TTGGTGGCATCGATTATCGG; R: GAGCACTTCTTTTGTGATGGC). We then used Sanger sequencing analysis to verify the PCR results (Yang et al., 2022). Supplementary Table S1 displays the pertinent primer sequences.

### 2.2. Antimicrobial susceptibility

Agar dilution and broth microdilution methods were used for antimicrobial susceptibility testing (AST), and *Escherichia coli* ATCC^®^ 25922^™^ was used as the control. AST results were interpreted based on the Clinical and Laboratory Standards Institute (CLSI) 2021 standards. Tigecycline and colistin, whose clinical breakpoints were based on the 2022 EUCAST.^4^ Supplementary Table S2 has been updated with the pertinent material information and details of antimicrobial susceptibility methodology.

### 2.3. Plasmid characterization and southern blotting and hybridization

S1-PFGE was undertaken on the CHEF-DR III system (Bio-Rad. Hercules, CA, United States), and patterns were evaluated and interpreted according to the published guidelines (Wang et al., 2019). The adjusted bacterial suspension was mixed with 1% Seakem Golden agarose and 1% sodium dodecyl sulfate (SDS), digested with proteinase K for 2 h at 56°C and then digested with S1 enzyme. The electrophoresis time was 16 h, the pulse time was from 2.16 s to 63 s, and a *Salmonella* serotype Braenderup strain (H9812) digested by Xba-I was used as the Marker. Then we used digoxigenin-labeled *bla*_IMP-4_, *bla*_NDM-1_, and *bla*_KPC-2_ probes made the dig-high prime DNA Labeling and Detection Starter Kit II (Roche Diagnostics, Swiss Confederation) to determine the location of plasmids harboring *bla*_IMP-4_, *bla*_NDM-1_, and *bla*_KPC-2_ via southern blotting and hybridization. *bla*_IMP-4_ (F: GTAGCATGCTACACCGCAGCAG; R: TCGTTAACCCTTTAACCGCC), *bla*_NDM-1_ (F: TGCCCAATTATGCACCCG; R: CCACGGTGATTTTCACTG), and *bla*_KPC-2_ (F: ATGTCACTGTATCGCCGTC; R:TTACTGCCCGTTGACGCC).

### 2.4. Conjugation assays

The transferability of plasmids was investigated by using, a NaN_3_-resistant standard strain, as the recipient for conjugation assays. To culture wang1, wang9, and *E. coli* J53, shake them and let them grow in the broth for 6 h until they reach the logarithmic growth phase. Then, add 100 microliters of wang1 and 200 microliters of *E. coli* J53 to the broth, and add 100 microliters of wang9 and 200 microliters of J53 to the broth. Culture both samples overnight at 37°. Subsequently, transconjugants carrying *bla*_IMP-4_, *bla*_NDM-1_, and *bla*_KPC-2_ were first selected using Mueller-Hinton agar (OXOID, Hampshire, United Kingdom) plates containing both 1 mg/L meropenem and 200 mg/L NaN_3_. Further, the selected transconjugant were confirmed by MALDI-TOF/MS, PCR identified the *bla*_IMP-4_, *bla*_NDM-1_, and *bla*_KPC-2_ genes, and AST was used to verify the expression of antimicrobial resistance genes.

### 2.5. Plasmid stability assays

Briefly, the isolated wang1 and wang9 strains were cultured in LB broth with shaking (180 rpm) at 37°C and then serially passaged daily at a dilution of 1:1,000 in antibiotic-free LB broth for 5 days. After 5 days, the cultures were inoculated on MH agar plates without antibiotics, and 188 single colonies were selected for PCR identification after culturing at 37° overnight.

### 2.6. Whole genome sequencing and *in silico* analyses

Genomic DNA was extracted using a Genomic DNA Isolation Kit (QIAGEN, Hilden, Germany) and sequenced using Illumina Novaseq 6000 (Illumina, San Diego, CA, United States) and Oxford Nanopore platforms (Oxford Nanopore Technologies, Oxford, United Kingdom). RAST 2.0 and Prokka were used to annotate the draft genomes obtained by SPAdes version 3.9.1 and Uncycler (Aziz et al., 2008; Seemann, 2014; Wick et al., 2017).^5^ ISfinder was used to detect insertion sequence elements and integrons.^6^ Antimicrobial resistance genes (ARGs) were identified by ResFinder.^7^ Plasmid identification were identified by PlasmidFinder 2.1.^8^ We found the plasmid sequence with the highest consistency with the plasmid pwang9-1 and pwang9-2 in this study using the NCBI blast tool. Different plasmid genome sequences were compared using the BLAST Ring Image Generator (BRIG) (Alikhan et al., 2011). The figures about the genetic context surrounding the antibiotic resistance genes were drawn by Easyfig 2.3 (Sullivan et al., 2011). Whole-genome sequencing data were imported into an online website, and MLST analysis was performed based on seven housekeeping genes.^9^ A report on both the quality of the sequences (wang1, Supplementary Figure S1; wang9, Supplementary Figure S2) and the quality of the assembly (Supplementary Table S3) has been included in the Supplementary material.

### 2.7. Phylogenetic analysis

We downloaded all *C. freundii* genomes (*n* = 42), plus 16 valid species of Citrobacter strains reference genomes,^10^ from China from public data on NCBI and conducted core genes research through Roary (Page et al., 2015). For the reliability of the data, we use average nucleotide identity (ANI) analysis for all data.^11^ Phylogenetic analysis was performed with these genomes, plus wang1 and wang9, by the maximum likelihood method on MEGA X. The resulting phylogenetic tree was modified by iTOL.^12^

## 3. Results

### 3.1. Species confirmation of strains

We isolated two carbapenem-resistant *C. freundii* strains from the cerebrospinal fluid of a 12-year-old patient with a meningococcal infection, post-pineal tumor surgery. Two strains of *C. freundii* were identified by MALDI-TOF-MS and WGS (ANI analysis, the ANI values for wang1 and wang9 were both similar to the reference genome of *C. freundii*, with values of 0.988 and 0.988, respectively, Supplementary Table S2), and designated as wang1 and wang9, respectively. Wang1 carried *bla*_KPC-2_, wang9 carried *bla*_KPC-2_, *bla*_NDM-1_, *bla*_OXA-1_, and *bla*_IMP-4_. This phenomenon aroused our curiosity, and we conducted in-depth research on wang1 and wang9, respectively.

### 3.2. AST of *Citrobacter freundii* wang1, wang9 and transconjugants wang1J1 and wang9J2

We identified the transconjugants by MALDI-TOF-MS and PCR, in which wang9J1 carried *bla*_KPC-2_, *bla*_IMP-4_, and *bla*_NDM-1_, while wang1J1 and wang9J2 carried *bla*_KPC-2_. The isolates wang1 and wang9 both displayed resistance to most of the antibiotics, for example penicillins, cephalosporins, carbapenems, amino-glycosides, fluorquinolones etc. classes. They also both displayed susceptibility to amikacin, fosfomycin and polymyxin B. For tigecycline, wang1 and wang9 were determined as intermediate. For gentamicin, imipenem and meropenem, the MIC values of wang9 were significantly higher than those of wang1. This is also proved by comparing the AST results of wang9J1 with wang9J2 and wang1J1. The results of the AST of *C. freundii* wang1, wang9 and transconjugants are shown in Table 1.

**Table 1:** MIC values of antimicrobials for *C. freundii* wang1 and wang9, recipient strain J53, transconjugants wang1J1, wang9J1, and wang9J2, and control strain *E. coli* ATCC^**®**^ 25922^**™**^.

Antimicrobials	MIC values (mg/L)		wang1J1	wang9J1	wang9J2	J53	ATCC® 25922™
	wang1	wang9					
Aztreonam	>128 (R)	>128 (R)	128 (R)	128 (R)	128 (R)	0.125 (S)	0.125 (S)
Imipenem	8 (R)	32 (R)	8 (R)	32 (R)	8 (R)	0.25 (S)	0.125 (S)
Meropenem	16 (R)	32 (R)	16 (R)	32 (R)	8 (R)	0.03 (S)	0.015 (S)
Ceftriaxone	>128 (R)	>128 (R)	128 (R)	>128 (R)	>128 (R)	0.06 (S)	0.125 (S)
Cefotaxime	>128 (R)	>128 (R)	128 (R)	>128 (R)	>128 (R)	0.25 (S)	0.25 (S)
Ceftazidime	>128 (R)	>128 (R)	>128 (R)	>128 (R)	>128 (R)	0.06 (S)	0.06 (S)
Levofloxacin	16 (R)	16 (R)	8 (R)	16 (R)	8 (R)	0.06 (S)	0.03 (S)
Ciprofloxacin	4 (R)	8 (R)	4 (R)	8 (R)	4 (R)	0.03 (S)	0.015 (S)
Amikacin	2 (S)	2 (S)	2 (S)	2 (S)	2 (S)	2 (S)	2 (S)
Gentamicin	16 (R)	64 (R)	16 (R)	64 (R)	16 (R)	2 (S)	1 (S)
P/T	128/4 (R)	>128/4 (R)	128/4 (R)	128/4 (R)	128/4 (R)	2/4 (S)	4/4 (S)
Fosfomycin	64/25 (S)	64/25 (S)	64/25 (S)	64/25 (S)	64/25 (S)	0.25/25 (S)	0.25/25 (S)
Chloromycin	128 (R)	128 (R)	128 (R)	128 (R)	128 (R)	4 (S)	4 (S)
T/S	8/152 (R)	8/152 (R)	8/152 (R)	8/152 (R)	8/152 (R)	0.25/4.75 (S)	0.25/4.75 (S)
AMC	>128/64 (R)	>128/64 (R)	128/64 (R)	>128/64 (R)	>128/64 (R)	2/1 (S)	4/1 (S)
Cefepime	>128 (R)	>128 (R)	>128 (R)	>128 (R)	>128 (R)	0.06 (S)	0.06 (S)
Tigecycline	2 (I)	2 (I)	2 (I)	2 (I)	2 (I)	0.06 (S)	0.03 (S)
Polymyxin B	1 (S)	1 (S)	1 (S)	1 (S)	1 (S)	1 (S)	1 (S)

R, resistant; S, susceptible; I, intermediate; T/S, Trimethoprim/Sulfamethoxazole; AMC, Amoxicillin-clavulanic acid; P/T, Piperacillin/Tazobactam.

### 3.3. MLST and genome of *Citrobacter freundii* isolates wang1 and wang9

According to the WGS results, wang1 and wang9 were shown by MLST to carry the genes *ar*cA (18), *aspC* (151), *clpX* (14), *dnaG* (9), *fadD* (33), *lysP* (11), *mdh* (29), confirming its typing as ST415.

As mentioned above, the results of S1-PFGE and WGS showed that isolates wang1 and wang9 both carried two plasmids of different sizes. We searched the whole genome of wang1 and wang9 by ResFinder and PlasmidFinder. Specific information is displayed in Table 2. Both wang1 and wang9 have genomes of 5,232,707 bp, 5,232,708 bp, respectively. Wang1 has three plasmids, named pwang1-1, pwang1-2, and pwang1-3, with sizes of 149,719 bp, 65,148 bp, and 4,782 bp, respectively. Wang9 contains three plasmids, designated pwang9-1, pwang9-2, and pwang9-3, with sizes 223,404 bp, 149,719 bp, and 4,782 bp, respectively. According to WGS data analysis, pwang1-1 and pwang9-2, pwang1-3, and pwang9-3, are exactly the same. The G + C contents of pwang1-2, pwang1-3, pwang9-1, and pwang9-2 were 54.4, 52.6, 49.1, and 52.6%, respectively. The pwang9-1 carried both *bla*_IMP-4_ and *bla*_NDM-1_, and the pwang9-2 carried *bla*_KPC-2_.

**Table 2:** Plasmid and genome information of *C. freundii* wang1 and wang9.

	Sizes (bp)	Type	G + C%	ARGs
wang1				
genome	5,232,707	ST415	51.8%	*bla*_CMY-48_
pwang1-1	149,719	IncFII	52.6%	*bla*_KPC-2_
pwang1-2	65,148	IncFIB (K)	54.4%	*sul1*, *aph(3″)-Ib*, *aph(6)-Id*, *aac(3)-IV*, *aph(4)-Ia*, *dfrA12*, *qacE*, *aadA2*
pwang1-3	4,782	Col	52.6%	–
wang9				
genome	5,232,708	ST415	51.8%	*bla*_CMY-48_
pwang9-1	223,404	IncHI1B	49.1%	*bla*_IMP-4_, *bla*_NDM-1_, *bla*_SFO-1_, *bla*_TEM-206_, *bla*_OXA-1_ *sul1*, *aph(3″)-Ib*, *aph(6)-Id*, *aac(6′)-Ib3*, *aph(3′)-VI*, *qacE*, *aac(3)-IId*, *mph(A)*, *mph(E)*, *aac(6′)-Ib-cr*, *catB3*, *msr(E)*, *arr-3*
pwang9-2	149,719	IncFII	52.6%	*bla*_KPC-2_
pwang9-3	4,782	Col	52.6%	*–*

### 3.4. S1-PFGE and southern blotting and hybridization

The S1-PFGE results demonstrated that that there are two plasmids in wang1, with sizes of 150 kb ~ and 70 kb~, whereas wang9 possesses two plasmids, with sizes of 230 kb ~ and 150 kb~, respectively. Interestingly, the second plasmid present in wang1 and the first plasmid of wang9 are of almost the same size.

Southern blotting and hybridisation results showed that *bla*_IMP-4_ and *bla*_NDM-1_ were both located on a 230 k ~ plasmid, and *bla*_KPC-2_ was located on a 150 kb ~ plasmid (Supplementary Figure S3). It was revealed by the results that wang1 carried *bla*_KPC-2_, whereas wang9 carried *bla*_KPC-2_, *bla*_IMP-4_, and *bla*_NDM-1_ simultaneously. The results were consistent with the WGS sequencing analysis.

### 3.5. Plasmid stability assays

The selected 188 colonies of wang1 and wang9 were subjected to PCR validation of *bla*_KPC-2_ and *bla*_IMP-4_. It was found that the preservation rate of pwang9-1 was 98.4%, while the preservation rate of pwang9-2 was 96.27% (Supplementary Table S4). This provides conclusive evidence that the resistant plasmid is capable of enduringly coexisting with and multiplying alongside the host bacteria, guaranteeing its stable existence and sustained expression over an extended period.

### 3.6. Structural characterization of the transferable plasmid

**Figure 1:**
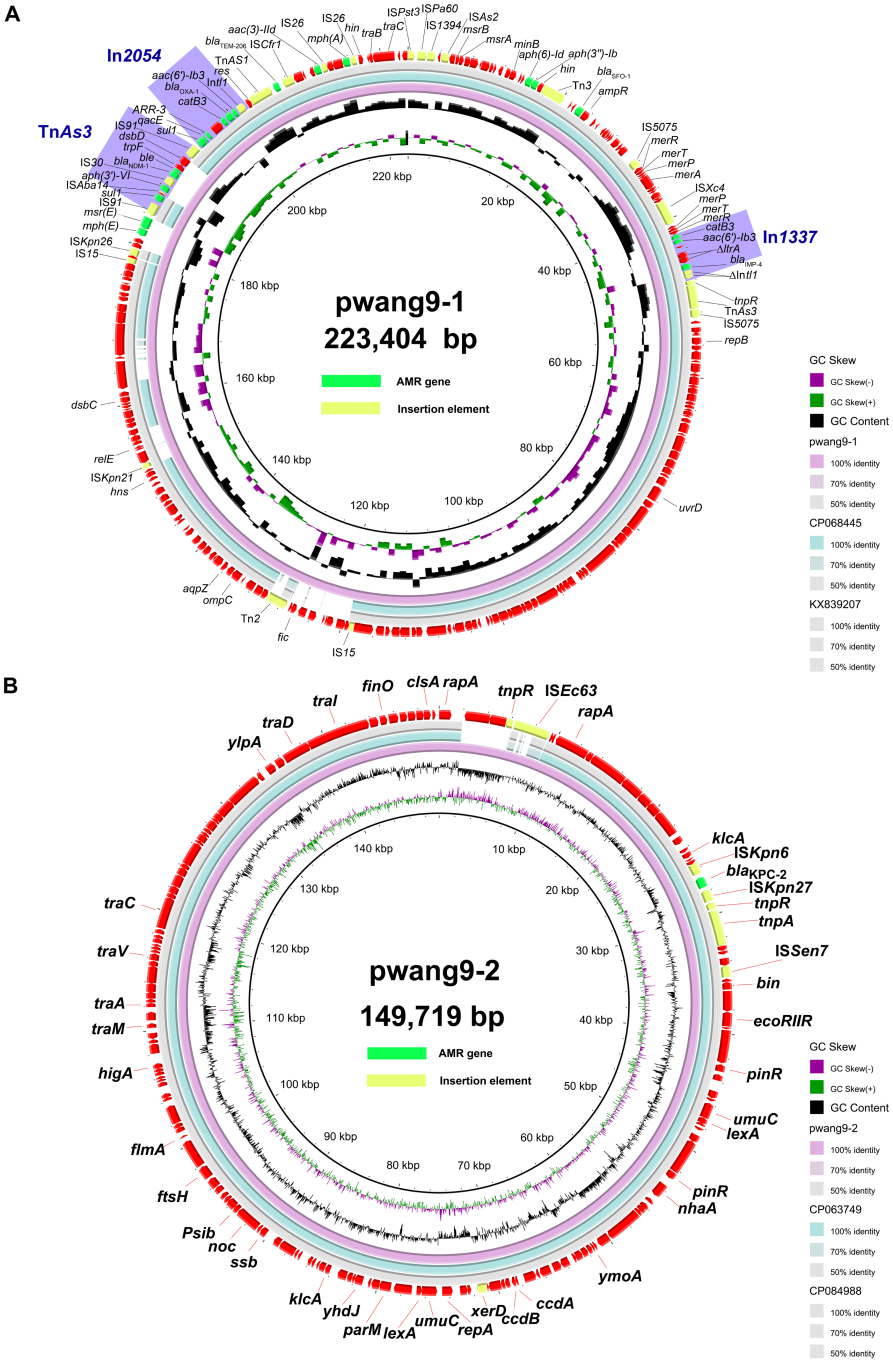
Genomic analyses of plasmid pwang9-1 **(A)** and pwang9-2 **(B)**. The comparative plasmid circular map of pwang9-1 **(A)** and pwang9-2 **(B)**, generated using BLAST Ring Image Generator (BRIG), shows the genes and their locations.

The plasmid pwang1-2 was identified as an IncFIB(K) type plasmid by PlasmidFinder analysis, while pwang9-1 and pwang9-2 could be classified into any of the known incompatibility groups. When we lowered the threshold for minimum % identity and the minimum % coverage of PlasmidFinder, pwang9-1 and pwang9-2 could have IncHI1B (the threshold for 75.22% identity and the 99.82% coverage) and IncFII (the threshold for 91.86% identity and the 96.09% coverage), respectively. The pwang9-1 carried *bla*_IMP-4_, *bla*_OXA-1_, and *bla*_NDM-1_. We found a replicon FIB downstream of IS*5075* in the plasmid and a replicon repAciN interrupted by IS*Kpn26* downstream of IS*15*, indicating that pwang9-1 might be a hybrid plasmid. Through ISfinder and INTEGRONF, we noticed that pwang9-1 has a lot of insertion sequences, transposons and integrons, such as Tn*AS3*, IS*15*, IS*26*, In*2054*, In*1337* and so on. The *bla*_IMP-4_ coexists with *bla*_NDM-1_ and *bla*_OXA-1_ on a novel transferable plasmid variant pwang9-1, located on integron In*1337* and transposon Tn*AS3*, and integron In*2054*, respectively. The gene cassette sequence of integron In*1337* is *IntI1-bla*_IMP-4_*-qacG2-aacA4′-catB3Δ*, while the gene cassette sequence of In*2054* is *IntI1-aacA4cr-bla*_OXA-1_*-catB3-arr3-qacEΔ1-sul1.* The *bla*_NDM-1_ is located on the transposon Tn*AS3*, and its sequence is IS*91-sul-*IS*Aba14-aph(3′)-VI-*IS*30-bla*_NDM-1_*-ble-trpF-dsbD-*IS*91*. The most similar plasmids identified by NCBI blast are as follows: pKP1814-1 from *Klebsiella pneumoniae* (GeneBank: KX839207, with 90% query coverage and 99.85% nucleotide identity) and pA from *Klebsiella quasipneumoniae* (GeneBank: CP068445, with 86% query coverage and 99.85% nucleotide identity). BLAST Ring Image Generator (BRIG) generated a circular image of multiple plasmid comparisons, as demonstrated in Figure 1A. We found that the main differences are concentrated in several gaps, and there were basically insertion sequences or transposons upstream and downstream of the gaps, such as IS*Kpn21*, Tn*2* and so on.

**Figure 2:**
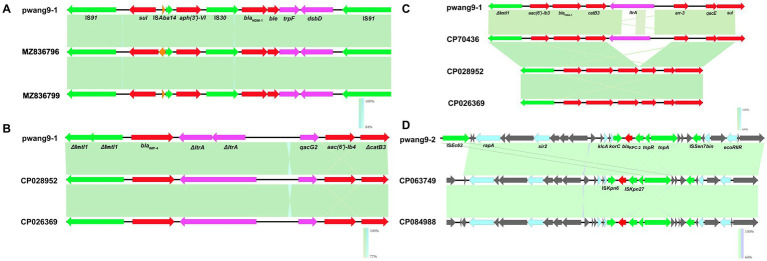
Genetic context of *bla*_NDM-1_ **(A)**, *bla*_IMP-4_ **(B)**, *bla*_OXA-1_ **(C)** on pwang9-1 and *bla*_KPC-2_ **(D)** on pwang9-2. Arrows denote genes. Genes, mobile elements, and other features are colored based on their functional classification.

At the same time, we used Easyfig 2.3 to study the upstream and downstream environments of major antibiotic resistance genes. Among these, the transposon Tn*As3* carrying *bla*_NDM-1_ is highly conserved (Figure 2A). The integrase of integron In*1337* and group II intron reverse transcriptase/maturase (*ItrA*) were both interrupted into two contiguous sequence fragments (Figure 2B). The sequence of the integron In*2054* is also highly conserved, but group II *ItrA* is inserted between *catB3* and *arr-3*. By comparing CP70436, groups II intron reverse transcriptase/maturase are not exactly the same (Figure 2C). The pwang9-1 could not be analyzed using oriTfinder, but the success of the conjugation experiment proved that it could conjugate autonomously and transfer across species.

The pwang9-2 carried only one antibiotic resistance gene, *bla*_KPC-2_, which is located on the transposon Tn*2*. The most similar plasmids identified by NCBI blast were follows: pKP19-3,023-142 K from *K. pneumoniae* (GeneBank: CP063749, with 95% query coverage and 100% nucleotide identity) and pkp18-2,110-2-2 from *K. pneumoniae* (GeneBank: CP084988, with 95% query coverage and 99.99% nucleotide identity). BLAST Ring Image Generator (BRIG) generated a circular image of multiple plasmid comparisons (Figure 1B). We found that the main differences were concentrated in one gap, and there was a Tn*3* family transposase (IS*Ec63*) downstream of the gap. At the same time, the upstream and downstream environment of *bla*_KPC-2_ was studied, and it was found to be located on the transposon Tn*2*. The sequence was highly conserved (Figure 2D). The sequence of Tn*2* is *klcA-korC-*IS*kpn6-bla*_KPC-2_*-*IS*kpn27-tnpR-tnpA.* At the same time, it was analyzed by oriTfinder that it has an autonomous conjugation module and can conjugate and transfer autonomously, which is consistent with the results of the conjugation experiment (Supplementary Figure S4). We also found class 1 integron In*27* on pwang1-2, whose gene cassette sequence is *IntI1-dfrA12-gcuF-aadA2-qacEΔ1-sul1-orf5.*

Although both wang1 and wang9 contain a 4,782 bp plasmid, pwang1-3, it is too small to contain any antibiotic resistance genes or virulence genes, therefore, is considered unimportant. The plasmid pwang1-3 was identified as an Col type plasmid by PlasmidFinder analysis. Because it does not contain any antibiotic resistance genes, the transferability of this plasmid cannot be checked by conjugation assays. We used oriTfinder to analyze the plasmid and found that it did not include type IV secretion system or type IV coupling protein (Supplementary Figure S5). We believe that this means it cannot be transferred.

### 3.7. Phylogenetic analysis

Based on the ANI results analysis, we believe that 10 strains are not part of *C. freundii* (Supplementary Figure S6, Supplementary Table S5). So, these 10 bacterial strains were excluded from the phylogenetic analysis.

**Figure 3:**
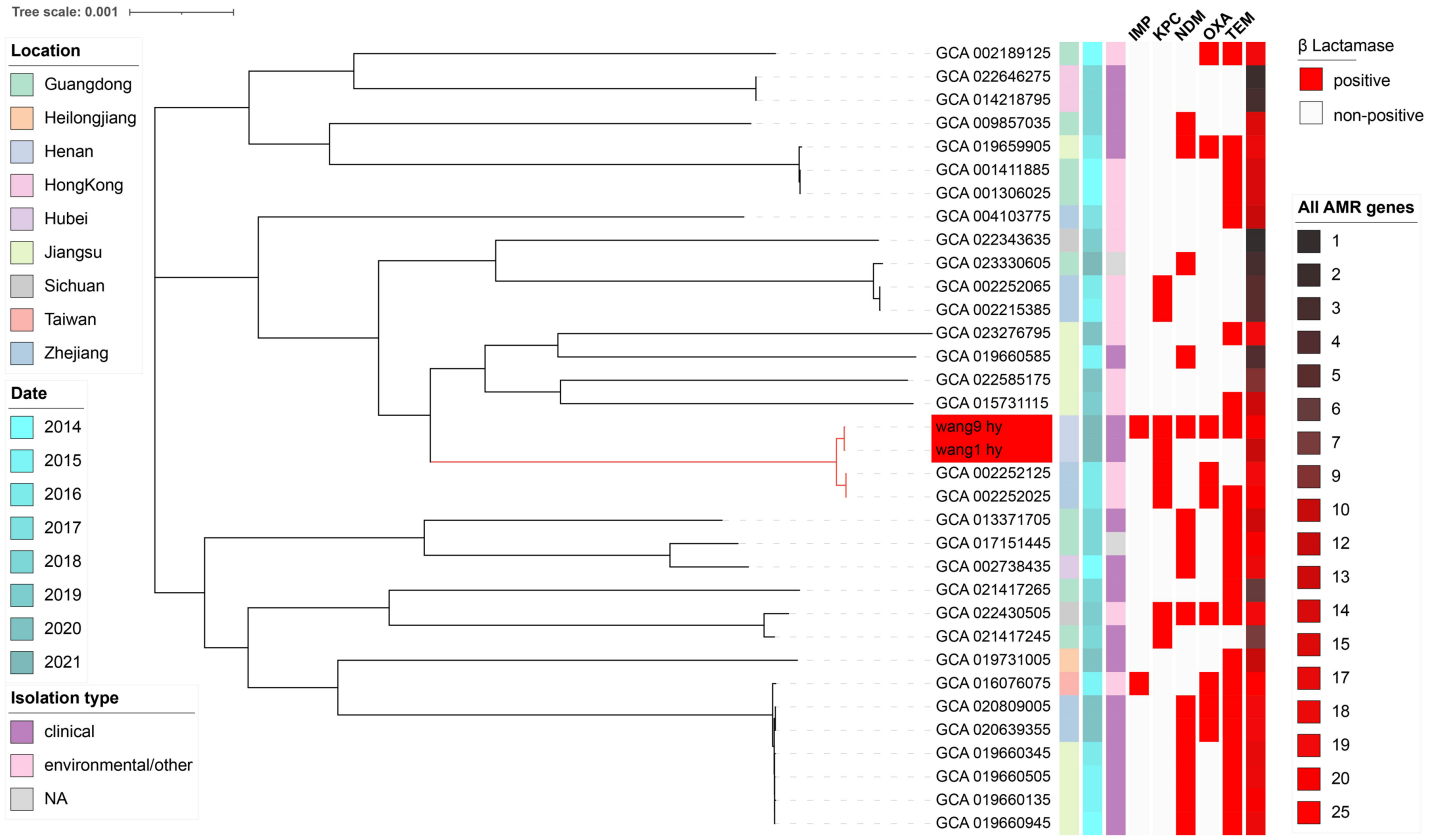
The phylogenetic tree of 32 strains of *C. freundii* from public data on NCBI and conducted core genes research through Roary. Phylogenetic analysis was performed with these genomes, plus wang1 and wang9, by the maximum likelihood method on MEGA X, and iTOL modified the resulting phylogenetic tree. We used different colors to represent different meanings. We marked wang1 and wang9 in red.

Phylogenetic analysis showed all 34 strains of *C. freundii* isolated from China were divided into three clusters (Figure 3). Among them, nine isolates were from Guangdong, nine isolates were isolated from Jiangsu, seven isolated from Zhejiang, and only two isolated from Henan. Overall, environmental and clinical isolates showed a clear segmental distribution. The data demonstrates that there are several small groups of *C. freundii* carrying genes for antibiotic resistance (*bla*_IMP_, *bla*_KPC_, *bla*_NDM_, *bla*_OXA_, and *bla*_TEM_) and all AMRs, which are in significantly greater numbers than other isolates. Among them, wang1 and wang9 belong to the same cluster as two strains of *C. freundii* from environmental samples from Zhejiang.

## 4. Discussion

*Citrobacter freundii* has become an important pathogen causing nosocomial infections, but its related research is not deep enough, especially *C. freundii* carrying multiple carbapenemases genes. We continuously collect CREs for a large tertiary teaching hospital in Henan, China. A strain of *C. freundii* carrying *bla*_KPC-2_, *bla*_IMP-4_, *bla*_OXA-1_, and *bla*_NDM-1_ was discovered in the cerebrospinal fluid of a 12-year-old post-operative brain tumor patient. After a pineal tumor surgery, the patient was treated with ceftriaxone for long-term anti-infective therapy. However, 8 days after surgery, the patient developed a meningococcal infection. Levofloxacin was added to the existing therapy to combat the infection. Nevertheless, the patient’s condition progressively worsened, and multidrug-resistant *C. freundii* was isolated 20 days post-surgery. The patient remained comatose for an extended period, and her condition further deteriorated 32 days after surgery. The family ultimately decided to discontinue treatment.

Our research on *C. freundii* has found that some isolates only carried *bla*_KPC-2_, but not *bla*_IMP-4_ or *bla*_NDM-1_. We hypothesized that the plasmids carrying *bla*_IMP_ and *bla*_NDM_ were lost and named the *C. freundii* carrying *bla*_KPC-2_ as wang1, and the *C. freundii* carrying *bla*_IMP-4_, *bla*_NDM-1_, *bla*_KPC-2_ as wang9. The WGS results and ANI analyze (Supplementary Figure S7, Supplementary Table S6) show that the chromosomal genomes of wang1 and wang9 are completely consistent, and pwang1-1 and pwang9-1 are also completely consistent. We also found that wang1 carries pwang1-2. Upon comparing it with pwang9-1, we found that it is very different, meaning that it is a plasmid unrelated to pwang9-1. We posited that the same *C. freundii* strain obtained different drug resistance plasmids during clinical treatment, thus manifesting different drug resistance profiles. Compared with wang1, and wang9, the MIC values of IMP, MEM and Gentamicin were significantly increased, which proved the coexistence of *bla*_KPC-2_, *bla*_NDM-1_ and *bla*_IMP-4_ would significantly enhance the drug resistance of the strains. The antimicrobial susceptibility results of the transconjugants wang9J1 and wang9J2 also supported the conclusion. For tigecycline, wang1 and wang9 were determined to be intermediate. The isolates wang1 and wang9 both displayed sensitivity to amikacin, fosfomycin and polymyxin B.

Plasmids can capture, assemble, maintain and disseminate genes associated with antibiotic resistance, heavy metal resistance, and virulence (Tarabai et al., 2021; Okoye et al., 2022). It is speculated that plasmids carrying multiple drug-resistance genes may impose higher adaptation costs on the host strain. The stability of the resistance plasmids was assessed to determine their potential to remain functional under antibiotic-free conditions. The plasmid pwang9-1, which contains the genes *bla*_IMP-4_ and *bla*_NDM-1_, showed high stability after serial passage for 5 days, with a retention rate of 98.4%. At the same time, pwang9-1 was analyzed by BRIG, and it was found to carry a large number of insertion sequences and drug-resistance genes. It is believed that this strain is likely to capture more drug-resistance genes, thereby enhancing its drug resistance. The main differences between pwang9-1 and CP068445, and KX839207 are located in a few sections containing transposons and insertion sequences. It is possible that the variation between pwang9-1 and the other two genomes is due to the capture of new and different genes by the insertion sequences and transposons. The upstream and downstream regions of *bla*_NDM-1_, *bla*_IMP-4_, and *bla*_OXA-1_ share a significant similarity, suggesting that transposons and integrons are key players in disseminating drug resistance. These results suggest that strains carrying pwang9-1 may be able to persist for long periods without affecting their fitness. Although oriTfinder could not analyze pwang9-1, the success of conjugation assays suggests that it can be horizontally spread across species. This raises concerns about an emerging drug resistance in the clinical setting.

The pwang9-2 is a plasmid that cannot be classified and carries *bla*_KPC-2_. The conjugation assays and oriTfinder analysis show that it can autonomously transfer and conjugate across species. The pwang9-2 plasmid, which contains the *bla*_KPC-2_ gene conferring resistance, showed high stability after 5 days of continuous passage, with a retention rate of 96.27%. This suggests that it can not only be autonomously transferred to different species, but also that the plasmid is highly stable once the transfer is successful, allowing it to spread widely among strains. This issue further compounds drug resistance and makes clinical treatment more difficult.

The results of the phylogenetic analysis showed that multidrug-resistant *C. freundii* infections are becoming more prevalent in China and that the drug resistance levels of both environmental and clinical strains have increased significantly. wang1 and wang9 form a subcluster with GCA 002252125.1 and GCA 002252025.1, suggesting that the environment is a reservoir for multidrug-resistant strains, and bacteria can spread to each other between the environment and the human body, which is consistent with previous research (Bi et al., 2015; Ji et al., 2019; Zheng et al., 2019). Although only 34 strains of *C. freundii* were isolated from China, our conclusions may not be sufficient. Although there have been articles reporting *C. freundii* carrying *bla*_IMP-4_, *bla*_NDM-1_, and *bla*_KPC-2_, a query of the strain information uploaded by NCBI reveals that the strain P10159 is not *C. freundii*, but *Citrobacter portucalensis*.

## 5. Conclusion

To the best of our knowledge, we are the first to find *C. freundii* carrying *bla*_IMP-4_, *bla*_NDM-1_, *bla*_OXA-1_ and *bla*_KPC-2_. Its drug resistance mechanism and molecular transfer mechanism have been studied in depth. We found that *bla*_IMP-4_, *bla*_OXA-1_, and *bla*_NDM-1_ coexist on a new transferable hybrid plasmid that carries many insertion sequences and drug-resistance genes. This may further capture more drug resistance genes and lead to the development of new drug resistance. The emergence of new drug-resistant strains is a cause for concern.

## Data availability statement

The datasets presented in this study can be found in online repositories. The names of the repository/repositories and accession number(s) can be found at: https://www.ncbi.nlm.nih.gov/, SAMN29992768; https://www.ncbi.nlm.nih.gov/, SAMN29992269.

## Author contributions

The experiments were conceived and designed by JG and BZ. The samples and experiments were collected and performed by JQ, YC, HG, RL, CL, and RC. The data was analyzed by HX and XG. The manuscript was written by JQ and revised by BZ. All authors contributed to the article and approved the submitted version.

## Funding

This work was supported by research grants from Henan Science and Technology Department (No. 192102310059), the National Natural Science Foundation of China (82072314), the Research Project of Jinan Microecological Biomedicine Shandong Laboratory (JNL-2022011B), the Fundamental Research Funds for the Central Universities (2022ZFJH003), CAMS Innovation Fund for Medical Sciences (2019-I2M-5-045), Henan Province Medical Science and Technology Research Project Joint Construction Project (No. LHGJ20190232), and Zhejiang Provincial Natural Science Foundation of China (LQ20H200003).

## Conflict of interest

The authors declare that the research was conducted in the absence of any commercial or financial relationships that could be construed as a potential conflict of interest.

## Publisher’s note

All claims expressed in this article are solely those of the authors and do not necessarily represent those of their affiliated organizations, or those of the publisher, the editors and the reviewers. Any product that may be evaluated in this article, or claim that may be made by its manufacturer, is not guaranteed or endorsed by the publisher.

## Footnotes

^1^ http://bldb.eu/BLDB.php?prot=B1

^3^ http://bldb.eu/BLDB.php?prot=A

^4^ http://www.eucast.org

^5^ http://rast.nmpdr.org/

^6^ https://www-is.biotoul.fr/

^7^ https://cge.food.dtu.dk/services/ResFinder/

^8^ https://cge.food.dtu.dk/services/PlasmidFinder/

^9^ https://cge.food.dtu.dk/services/MLST/

^10^ https://lpsn.dsmz.de/genus/citrobacter

^11^ https://github.com/widdowquinn/pyani

^12^ https://itol.embl.de/
