# Supplementary material for: Coexistence of blaIMP-4, blaNDM-1 and blaOXA-1 in blaKPC-2-producing Citrobacter freundii of clinical origin in China
Source: Front Microbiol. 2023 Jun 12;14:1074612. doi: 10.3389/fmicb.2023.1074612 (PMC10291173; doi:10.3389/fmicb.2023.1074612)
Supplement: Supplementary file 1 [file Data_Sheet_1.zip › Table S5.docx]

| Table S5. all C. freundii genomes from China from public data on NCBI | | | | | | | | | | |
| --- | --- | --- | --- | --- | --- | --- | --- | --- | --- | --- |
| Assembly | Location | Date | Isolation type | *bla*_IMP_ | *bla*_KPC_ | *bla*_NDM_ | *bla*_OXA_ | *bla*_TEM_ | AMR genes | AMR genotypes |
| GCA_001922445.1 | Guangdong | 2015-09 | environmental/other | 0 | 0 | 0 | 0 | 0 | 2 | *bla*_CFE_, *qnrB* |
| GCA_009856695.1 | Guangdong | 2015 | environmental/other | 0 | 0 | 0 | 0 | 1 | 10 | *aac(3)-IId, aadA2, bla*_CMY-2_, *bla*_TEM-1_*, catA1, dfrA12, fosA3*  *, mph(A), qnrB9, sul1, tet(B)* |
| GCA_021417265.1 | Guangdong | 2018-12 | clinical | 0 | 0 | 0 | 0 | 1 | 6 | *aadA2, bla*_CMY_*, bla*_TEM-1_*, dfrA12, qnrS1, sul1* |
| GCA_002189125.1 | Guangdong | 2014 | environmental/other | 0 | 0 | 0 | 1 | 1 | 19 | *aac(3)-IVa, aac(6')-Ib-cr5, aac(6')-If, aadA1,*  *aadA2, ant(2'')-Ia, sul3, tet(A), sul1, sul2, qnrA1*  *aph(3')-Ia, aph(4)-Ia, arr-3, blaCMY-151,*  *bla*_OXA-1_*, bleO, catB3, cmlA1, dfrA12, floR, fosA7,*  *mcr-1.1, mcr-3.19, oqxA, oqxB,* |
| GCA_021417245.1 | Guangdong | 2018-12 | clinical | 0 | 1 | 0 | 0 | 0 | 7 | *aadA2, bla*_CMY_*, bla*_KPC-2_*, dfrA12, dfrA1, qnrB4, sul1* |
| GCA_023330605.1 | Guangdong | 2021-07 | NA | 0 | 0 | 1 | 0 | 0 | 3 | *bla*_CMY_*, bla*_NDM-1_*, ble* |
| GCA_001306025.1 | Guangdong | 2014-08 | environmental/other | 0 | 0 | 0 | 0 | 1 | 14 | *aac(6')-Ib-cr5, aadA16, aph(3'')-Ib, aph(6)-Id, arr-3,*  *bla*_CMY,_ *bla*_TEM-1_*, dfrA27, floR, qnrB6, sul1, sul2, tet(A)*  *, tet(D)* |
| GCA_001411885.1 | Guangdong | 2014-08 | environmental/other | 0 | 0 | 0 | 0 | 1 | 14 | *aac(6')-Ib-cr5, aadA16, aph(3'')-Ib, aph(6)-Id, arr-3,*  *bla*_CMY_*, bla*_TEM-1_*, dfrA27, floR, qnrB6, sul1, sul2, tet(A),*  *tet(D)* |
| GCA_017151445.1 | Guangdong | 2018-11 | NA | 0 | 0 | 1 | 0 | 1 | 20 | *aac(3)-IId, aac(6')-Ib-cr5, aadA16, aph(3'')-Ib,*  *aph(3')-Ia, aph(6)-Id, arr-3, bla*_CMY-152_*, mph(A),*  *bla*_CTX-M-3_*, bla*_EC_, *qnrS1, sul1, sul2, tet(A), floR,*  *bla*_NDM-5_*, bla*_TEM-1_*, ble, dfrA27* |
| GCA_017151595.1 | Guangdong | 2019-02 | environmental/other | 0 | 0 | 1 | 0 | 1 | 26 | *aac(3)-IId, aac(6')-Ib3, aadA22, aadA2, aph(3')-Ia,*  *aph(3')-Ia, aph(6)-Id, arr-2, arr-3, blaCMY-35, sul3,*  *bla*_CTX-M_*, bla*_LAP-2_*, floR, lnu(F), mph(A), sul1, sul2, qnrB9*  *bla*_NDM-5_*, bla*_TEM-1_*, ble, dfrA12, dfrA14, qnrB91, tet(A)* |
| GCA_013371705.1 | Guangdong | 2018-08 | clinical | 0 | 0 | 1 | 0 | 1 | 13 | *aac(3)-IId, aph(3'')-Ib, aph(6)-Id, bla*_CMY-152_*, tet(A)*  *bla*_CTX-M-55_*, bla*_NDM-1_*, bla*_SHV-12_*, bla*_TEM_*, ble, floR, fosA3, sul2,* |
| GCA_009857035.1 | Guangdong | 2018-03 | clinical | 0 | 0 | 1 | 0 | 0 | 15 | *aac(3)-IId, aac(6')-Ib-cr5, aadA16, aph(3'')-Ib,*  *aph(6)-Id, arr-3, bla*_CMY-78_*, bla*_NDM-1_*, ble,*  *catB3, dfrA27, fosA7, mph(A), sul1, sul2* |
| GCA_020683225.1 | Guangxi | 2020-10 | environmental/other | 0 | 0 | 0 | 0 | 0 | 9 | *aac(3)-IId, aadA2, bla*_CMY_*, bla*_TEM-1_*, dfrA12,*  *mph(A), qnrB6, sul1, sul2* |
| GCA_019731005.1 | Heilongjiang | 2020-03 | clinical | 0 | 0 | 0 | 0 | 1 | 10 | *aph(3'')-Ib, aph(6)-Id, bla*_CMY-72_*, bla*_LAP-2_*,*  *bla*_TEM-1_*, catA2, dfrA14, qnrS1, sul2, tet(A)* |
| wang1hy | Henan | 2021-09 | clinical | 0 | 1 | 0 | 0 | 0 | 10 | DDDDJGJ454KK |
| wang9hy | Henan | 2021-09 | clinical | 1 | 1 | 1 | 1 | 1 | 20 | YYYUK455 |
| GCA_022646275.1 | HongKong | 2018 | clinical | 0 | 0 | 0 | 0 | 0 | 2 | *bla*_CMY-48,_ *tet(X4)* |
| GCA_014218795.1 | HongKong | 2018 | clinical | 0 | 0 | 0 | 0 | 0 | 3 | *bla*_CMY-48_*, floR, tet(X4)* |
| GCA_015894005.1 | HongKong | 2012-12 | clinical | 1 | 0 | 0 | 0 | 1 | 7 | *bla*_CMY-86_*, bla*_IMP-4_*, bla*_LAP-2_*, bla*_TEM-1_*, qnrB30,*  *qnrS1, tet(D)* |
| GCA_015893905.1 | HongKong | 2012-05 | clinical | 0 | 0 | 1 | 0 | 1 | 21 | *aac(3)-IId, aadA2, aadA2, aph(3'')-Ib, aph(3')-I, ble,*  *aph(6)-Id, bla*_CMY_*, bla*_DHA-1_*, bla*_NDM-1_*, bla*_SHV-12_*, sul1, qnrB4,*  *bla*_TEM-1_*, catA2, dfrA12, dfrA12, dfrA19, mcr-9.1,*  *mph(A), qnrB13,* |
| GCA_002738435.1 | Hubei | 2014 | clinical | 0 | 0 | 1 | 0 | 1 | 18 | *aac(3)-IId, aph(3'')-Ib, aph(6)-Id, armA, bla*_CMY-152_*,*  *bla*_NDM-1_*, bla*_SFO-1_*, bla*_TEM-1_*, bla*_VEB-3_*, ble, sul1, qnrS1,*  *dfrA12, dfrA14, fosA3, mph(A), mph(E), msr(E),* |
| GCA_019795045.1 | Hunan | 2019-04 | clinical | 0 | 0 | 0 | 0 | 0 | 2 | *bla*_CMY_*, qnrB75* |
| GCA_022585175.1 | Jiangsu | 2020 | environmental/other | 0 | 0 | 0 | 0 | 0 | 9 | *aph(3'')-Ib, aph(6)-Id, bla*_CMY_*, catA2, sul2, tet(A)*  *dfrA14, floR, qnrS1,* |
| GCA_019660585.1 | Jiangsu | 2015 | clinical | 0 | 0 | 1 | 0 | 0 | 4 | *bla*_CMY,_ *bla*_NDM-1_*, bla*_SHV-12_*, ble* |
| GCA_015731115.1 | Jiangsu | 2019 | environmental/other | 0 | 0 | 0 | 0 | 1 | 13 | a*adA2, aph(3'')-Ib, aph(3')-Ia, aph(6)-Id, bla*_CMY-48_*,*  *bla*_TEM-1_*, dfrA12, floR, qnrS2, sul1, sul2, tet(D), tet(X4)* |
| GCA_019659905.1 | Jiangsu | 2016 | clinical | 0 | 0 | 1 | 1 | 1 | 18 | *aac(6')-Ib-cr5, aadA5, aph(3')-Ia, arr-3, bla*_CMY_,  *bla*_DHA-1_*, bla*_NDM-1_*, bla*_OXA-1_*, bla*_SHV-12_*, sul1, tet(A)*  *blaTEM-1, ble, catB3, dfrA17, floR, mph(A), qnrB4,* |
| GCA_023276795.1 | Jiangsu | 2020 | environmental/other | 0 | 0 | 0 | 0 | 1 | 19 | *aac(3)-IId, aac(6')-Ib-cr5, aadA16, aadA2, aph(3'')-Ib,*  *aph(6)-Id, arr-3, bla*_CMY_*, bla*_SHV-12_*, bla*_TEM-1_*, tet(X4)*  *dfrA27, floR, lnu(F), mph(A), qnrB6, sul1, sul2, tet(A),* |
| GCA_019660345.1 | Jiangsu | 2016 | clinical | 0 | 0 | 1 | 0 | 1 | 17 | *aac(3)-IId, aadA1, aadA2, bla*_CMY-48_*, bla*_DHA-1_*, sul1, sul2*  *bla*_NDM-1_*, bla*_SHV-12_*, bla*_TEM-1_*, ble, catA2, dfrA12, sat2,*  *dfrA1, mph(A), qnrB4,* |
| GCA_019660505.1 | Jiangsu | 2015 | clinical | 0 | 0 | 1 | 0 | 1 | 18 | *aac(3)-IId, aadA1, aadA2, bla*_CMY-48_*, bla*_DHA-1_*,*  *bla*_NDM-1_*, bla*_SHV-12_*, bla*_TEM-1_*, ble, catA2, dfrA12,*  *dfrA1, mph(A), qnrB4, sat2, sul1, sul2, tet(D)* |
| GCA_019660945.1 | Jiangsu | 2014 | clinical | 0 | 0 | 1 | 0 | 1 | 18 | *aac(3)-IId, aadA1, aadA2, bla*_CMY-48_*, bla*_DHA-1_*,*  *bla*_NDM-1_*, bla*_SHV-12,_ *bla*_TEM-1_*, ble, catA2, dfrA12,*  *dfrA1, mph(A), qnrB4, sat2, sul1, sul2, tet(D)* |
| GCA_019660135.1 | Jiangsu | 2015 | clinical | 0 | 0 | 1 | 0 | 1 | 20 | *aac(3)-IId, aac(3)-IIg, aac(6')-IIc, aac(6')-Ib3, aadA1,*  *aph(3'')-Ib, aph(3')-Ia, aph(6)-Id, arr, bla*_CMY-48_*, sul1*  *bla*_DHA-1_*, bla*_NDM-1_*, bla*_SHV-12_*, bla*_TEM-1_*, ble, tet(D), sul2,*  *catA2, dfrA19, dfrA1, ere(A), mcr-9.1, qnrB4, sat2,* |
| GCA_011077955.1 | Shandong | 2018 | environmental/other | 0 | 0 | 0 | 0 | 0 | 13 | *aac(6')-Ib-cr5, aadA16, aph(6)-Id, arr-3, qnrB6, sul1,*  *catA, dfrA27, floR, mph(A), oqxB, tet(A), vat* |
| GCA_011077325.1 | Shandong | 2018 | environmental/other | 0 | 0 | 1 | 0 | 0 | 16 | *aac(6')-Ib-cr5, aadA16, aph(6)-Id, arr-3, tet(A), vat*  *bla, bla*_NDM-5_*, ble, catA, dfrA27, floR, qnrB6, sul1,*  *mph(A), oqxB,* |
| GCA_001273815.1 | Sichuan | 2015-01 | environmental/other | 0 | 1 | 1 | 1 | 1 | 17 | *aac(6')-Ib-cr5, arr-3, bla*_CMY-137_*, bla*_CMY-6_*, qnrB10, sul1*  *bla*_CTX-M-12_*, bla*_CTX-M-14_*, bla*_KPC-2_*, bla*_NDM-1_*, mph(A), fosA3,*  *bla*_OXA-1_*, bla*_SHV-12_*, bla*_TEM_*, ble, catB3,* |
| GCA_022343635.1 | Sichuan | 2019-02 | environmental/other | 0 | 0 | 0 | 0 | 0 | 1 | *bla*_CMY-48_ |
| GCA_022430505.1 | Sichuan | 2019-08 | environmental/other | 0 | 1 | 1 | 1 | 1 | 19 | *aac(3)-IId, aac(6')-Ib-cr5, aadA2, arr-3, bla*_CMY_*,*  *bla*_CTX-M-14_*, bla*_KPC-2_*, bla*_NDM-1_*, bla*_OXA-1_*, sul2, tet(D), qnrA1,*  *bla*_SHV-12_*, bla*_TEM-1_*, ble, catB3, dfrA12, mph(A), sul1,* |
| GCA_016076075.2 | Taiwan | 2015 | environmental/other | 1 | 0 | 0 | 1 | 1 | 25 | *aac(3)-IId, aac(6')-Ib4, aac(6')-Ib, aadA2, ampC*  *, armA, bla*_CMY-48_*, bla*_CMY_*, bla*_CTX-M-3_*, bla*_GES-5_*, sul2, tet(D)*  *bla*_IMP-8_*, bla*_OXA-1_*, bla*_SHV-12_*, bla*_TEM-1_*, catA2, catB3, dfrA12,*  *dfrA1, mph(A), mph(E), msr(E), qnrB2, sul1, sat2,* |
| GCA_020639355.1 | Zhejiang | 2020-01 | clinical | 0 | 0 | 1 | 1 | 1 | 19 | *aac(3)-IId, aac(6')-Ib-cr5, aadA1, arr-3, bla*_CMY-48_*, sat2,*  *bla*_DHA-1_*, bla*_NDM-5_*, bla*_OXA-1_*, bla*_TEM-1_*, ble, tet(D), sul2, sul1,*  *catA2, catB3, dfrA1, mph(A), qnrB4,* |
| GCA_020809005.1 | Zhejiang | 2020-01 | clinical | 0 | 0 | 1 | 1 | 1 | 19 | *aac(3)-IId, aac(6')-Ib-cr5, aadA1, arr-3, bla*_CMY-48,_  *bla*_DHA-1_*, bla*_NDM-5_*, bla*_OXA-1_*, bla*_TEM-1_*, ble, tet(D), sul1, sul2,*  *catA2, catB3, dfrA1, mph(A), qnrB4, sat2,* |
| GCA_004103775.1 | Zhejiang | 2017 | environmental/other | 0 | 0 | 0 | 0 | 1 | 12 | *aac(3)-IId, aadA2, bla*_CMY_*, bla*_DHA-1_*, bla*_TEM-1_*, sul2, tet(D)*  *catA2, dfrA12, mph(A), qnrB4, sul1,* |
| GCA_002252065.1 | Zhejiang | 2016-01 | environmental/other | 0 | 1 | 0 | 0 | 0 | 5 | *bla*_CMY_*, bla*_KPC-2,_ *tmexC2, tmexD2, toprJ2* |
| GCA_002252125.1 | Zhejiang | 2016-01 | environmental/other | 0 | 1 | 0 | 1 | 0 | 19 | aac(6')-Ib-cr5, aadA5, armA, arr-3, blaCMY,  *bla*_CTX-M-14_*, bla*_KPC-2_*, bla*_OXA-1_*, bla*_TEM-1_*,*  *catB3, dfrA17, fosA3, mph(A), mph(E), msr(E), qnrB32,*  *tmexC2, tmexD2, toprJ2* |
| GCA_002252025.1 | Zhejiang | 2016-01 | environmental/other | 0 | 1 | 0 | 1 | 1 | 20 | *aac(6')-Ib-cr5, aadA5, armA, arr-3, bla*_CMY_*, tmexC2,*  *bla*_CTX-M-14_*, bla*_KPC-2_*, bla*_OXA-1_*, bla*_TEM-1_*, tmexD2, toprJ2*  *catB3, dfrA17, fosA3, mph(A), mph(E), msr(E), qnrB32,*  *sul1,* |
| GCA_002215385.1 | Zhejiang | 2015-01 | environmental/other | 0 | 1 | 0 | 0 | 0 | 5 | *bla*_CMY_*, bla*_KPC-2_*, tmexC2, tmexD2, toprJ2* |
| Citrobacter_braakii |  |  | GCF_009648935 |  |  |  |  |  |  |  |
| Citrobacter europaeus |  |  | GCF_003795375 |  |  |  |  |  |  |  |
| Citrobacter portucalensis |  |  | GCF_008693605 |  |  |  |  |  |  |  |
| Citrobacter freundii |  |  | GCF_003812345 |  |  |  |  |  |  |  |
| Citrobacter_sedlakii |  |  | GCF_018128425 |  |  |  |  |  |  |  |
| Citrobacter_gillenii |  |  | GCF_003429605 |  |  |  |  |  |  |  |
| Citrobacter_murliniae |  |  | GCF_004801125 |  |  |  |  |  |  |  |
| Citrobacter_cronae |  |  | GCF_016893685 |  |  |  |  |  |  |  |
| Citrobacter_werkmanii |  |  | GCF_008693645 |  |  |  |  |  |  |  |
| Citrobacter_pasteurii |  |  | GCF_019047765 |  |  |  |  |  |  |  |
| Citrobacter_koseri |  |  | GCF_000018045 |  |  |  |  |  |  |  |
| Citrobacter_amalonaticus |  |  | GCF_001558935 |  |  |  |  |  |  |  |
| Citrobacter_rodentium |  |  | GCF_021278985 |  |  |  |  |  |  |  |
| Citrobacter_farmeri |  |  | GCF_003938205 |  |  |  |  |  |  |  |
| Citrobacter_youngae |  |  | GCF_900638065 |  |  |  |  |  |  |  |
| Citrobacter_telavivensis |  |  | GCF_009295665 |  |  |  |  |  |  |  |
