# Supplementary material for: Coexistence of blaIMP-4, blaNDM-1 and blaOXA-1 in blaKPC-2-producing Citrobacter freundii of clinical origin in China
Source: Front Microbiol. 2023 Jun 12;14:1074612. doi: 10.3389/fmicb.2023.1074612 (PMC10291173; doi:10.3389/fmicb.2023.1074612)
Supplement: Supplementary file 1 [file Data_Sheet_1.zip › Table S6.docx]

wang1genome wang9genome

wang1genome 1.0 1.0

wang9genome 1.0 1.0

Citrobacter_cronae GCA_020683225 GCA_020809005 Citrobacter_farmeri GCA_023276795 Citrobacter_gillenii GCA_023330605 Citrobacter_europaeus wang1_hy wang9_hy Citrobacter_koseri Citrobacter_amalonaticus Citrobacter_murliniae Citrobacter_pasteurii Citrobacter_portucalensis Citrobacter_rodentium GCA_019795045 Citrobacter_sedlakii Citrobacter_freundii Citrobacter_braakii Citrobacter_telavivensis GCA_020639355 Citrobacter_werkmanii Citrobacter_youngae GCA_001273815 GCA_001306025 GCA_001411885 GCA_001922445 GCA_002189125 GCA_002215385 GCA_002252025 GCA_002252065 GCA_002252125 GCA_002738435 GCA_004103775 GCA_009856695 GCA_009857035 GCA_011077325 GCA_011077955 GCA_013371705 GCA_014218795 GCA_015731115 GCA_015893905 GCA_015894005 GCA_016076075 GCA_017151445 GCA_017151595 GCA_019659905 GCA_019660135 GCA_019660345 GCA_019660505 GCA_019660585 GCA_019660945 GCA_019731005 GCA_021417245 GCA_021417265 GCA_022343635 GCA_022430505 GCA_022585175 GCA_022646275

Citrobacter_cronae 1.0 0.9087827480719795 0.9066866743002545 0.8217013910993219 0.9064295453364509 0.8646954734286485 0.9061582447792758 0.9094518848957024 0.9062601163391932 0.9062624534643228 0.8361854175588865 0.8261400842105263 0.8639166339602072 0.8861385868976719 0.9082876326103998 0.821221089420655 0.9083701422291184 0.8206109923664121 0.9061549092303759 0.9090744966442952 0.8253879680915113 0.9066903384223919 0.992337929921623 0.8874048255051168 0.9081881685363945 0.9065121380731581 0.9064477634630251 0.9097040802422406 0.9060025045184612 0.9061913261554623 0.9063004268292683 0.9057778893008476 0.9061338277511961 0.9053764817708334 0.9060396718790054 0.9076451836195508 0.9063618461538461 0.7732771765175719 0.7732984378745504 0.9062075395795485 0.9063385186136073 0.9065745718654435 0.9075627578001058 0.907799080520861 0.9059453276353275 0.9059812412542109 0.9071523746081505 0.9064715859030839 0.9067256902356903 0.9066237614913176 0.906630599642766 0.906264299847793 0.906633976026524 0.9061271681643133 0.9066164008394545 0.9069781526836893 0.9057808718080991 0.906657595169336 0.9061995860118284 0.90632071117933

GCA_020683225 0.9088325596919127 1.0 0.9457666887888125 0.8231283852606351 0.9434033961802155 0.8657877777777779 0.9440085739217153 0.9279536130592504 0.9428031607495069 0.9428374280796655 0.835210616397367 0.8259233126660763 0.8651859533175977 0.892763921309744 0.9823328119092628 0.8215859337833494 0.989769821145574 0.8213320145852326 0.9430002193516939 0.9239220812053115 0.8261223580394501 0.9457677625029628 0.9075038226452907 0.8968136396771675 0.9242132724505326 0.9443843313521546 0.9442992395343077 0.9284068073032321 0.9440651851851852 0.9440550802271044 0.942619512195122 0.9432703269278762 0.9425169685534591 0.943819599009901 0.9454084511132623 0.9743274653937947 0.9433902507232402 0.7757418696883852 0.7757667570850203 0.9449364682734873 0.9442665056678167 0.9436081137578998 0.9830033564975678 0.9828381479702136 0.9448167433325177 0.944248301006629 0.9748084460260974 0.9443016703896748 0.9450220325796257 0.9458232550898203 0.9455325781063677 0.9431903067632851 0.9457141494968855 0.9446043460964696 0.9443740849267446 0.9456962416107382 0.9434812868832135 0.9455709663760896 0.9432730561687517 0.9442082610825374

GCA_020809005 0.9067139635535307 0.9451075397767753 1.0 0.8220315600947308 0.9858364315257353 0.8655991218354432 0.9868016861007461 0.9258066658595643 0.9862139725085911 0.9861133962693359 0.8350396906603215 0.8283950584212026 0.8640505535248042 0.900911785804496 0.9447324783147462 0.8207109516129033 0.9457999610231426 0.8229420041941282 0.9879447767346003 0.9237007133885957 0.8264400231147068 0.9999981000387748 0.9054406822128505 0.9069849811699723 0.9237904904157331 0.987713515909091 0.9876716806913806 0.9255910555826427 0.9888824820726347 0.9871140056351255 0.9867735100286532 0.9872989995247149 0.9867051863354037 0.9881934937332141 0.9890261088799643 0.9449693455174116 0.9871776808035715 0.7797859899068325 0.7758676886606085 0.9888857202921 0.9882940692640692 0.9869128469505178 0.9454776878191102 0.9449985586034914 0.9990413605015676 0.9892604344949273 0.9451798983789016 0.9885781124407317 0.9994034448296961 0.9994102692384971 0.9997736190666934 0.985793463963964 0.999784983974359 0.988229979619565 0.9885961806335655 0.9883583218492998 0.9878426589057044 0.9883188328912468 0.9877010048735205 0.9881737653200182

Citrobacter_farmeri 0.8219565269104966 0.8231402230259193 0.822261248142645 1.0 0.8219916497613367 0.8223289273877674 0.8228102558762274 0.8219987956309284 0.8221559329017069 0.8220951970588236 0.8330663924618605 0.9125117335631618 0.8193093356848911 0.821915821958457 0.8223657001201925 0.8428656590509667 0.8219849774164409 0.8412813082287308 0.822723181547619 0.8228272875436554 0.9377927147525676 0.822278273915627 0.822460477306003 0.8214060844986613 0.8221905726092089 0.8220912636874814 0.8219953420195439 0.8225856104486087 0.8220306968538665 0.8222169549657025 0.8226444228468901 0.8221149910394268 0.8225703741394792 0.8218290531314929 0.8225517398945519 0.8232789056376244 0.8221015393689177 0.7718245906656465 0.7718243687834736 0.8227080505415163 0.8218509061102832 0.8219357761947166 0.8224692900485439 0.8229612884731443 0.8221463454653938 0.8224206630984242 0.8219489991009888 0.8221697038791826 0.8226311080999698 0.8222336409346348 0.8222629259589652 0.8222371838407496 0.8222956747919145 0.8227766686320754 0.8224532909144717 0.8225710996765656 0.8223547610619468 0.8217498170551786 0.8222982084690553 0.8218126674605537

GCA_023276795 0.9057624721430422 0.9427341770909984 0.9861087356057117 0.8218020005945303 1.0 0.8643066464059197 0.9876834652082848 0.9235788348803127 0.9903073762486126 0.990177804121427 0.8339358876811593 0.8239190324466529 0.863597311684855 0.8997985639958378 0.9423886308190192 0.8193890462519937 0.9436085764499121 0.8208811824729892 0.9894829573652156 0.9229537272494233 0.8251274578277599 0.9861095232611701 0.9048984469696969 0.9057756049638056 0.9216357778902101 0.9866643788748565 0.9865651309141019 0.9246471794871794 0.9888374690348211 0.9889967512217827 0.9896457629427792 0.9890592354312355 0.9895970574869348 0.9869484312807305 0.9887698457000228 0.9431525808878857 0.9871825165714284 0.7754969107282693 0.7756085674822416 0.9867864001881026 0.9884296071180958 0.9914257581147176 0.9438246301369864 0.9419333082706767 0.9868397674959312 0.9871344731332867 0.9437276834637964 0.9863466421173763 0.9856878738865448 0.9862613743093923 0.9861937993079585 0.9890072196313568 0.9862100507380075 0.9879573433466322 0.9874794210150108 0.9870803682584937 0.989807495507637 0.9854477573358175 0.9912227319017046 0.9884245454545455

Citrobacter_gillenii 0.8644284434852979 0.8657339361423713 0.8647566984379137 0.82173212012012 0.8642834603174603 1.0 0.8644297539875642 0.8634661466942148 0.8624610832680764 0.862710494246862 0.8341826399510555 0.8235979204892967 0.8674761938469915 0.8648155495397942 0.8637205955463491 0.8201211740375438 0.8644283758043758 0.8183227485380117 0.8648293382546797 0.8656614121114684 0.8233554747774483 0.8647788724192694 0.8626671572527183 0.864027064171123 0.864824941718623 0.8647046509158482 0.8647079102496018 0.8632196987795377 0.8645011522086216 0.8642420172367358 0.8646473471008738 0.8643123940620783 0.8646209962904082 0.864193025682183 0.8635604464751958 0.8638514470557169 0.8642409008769599 0.7699937265093963 0.7700346541383448 0.8649997411701267 0.8651891597979261 0.8644645093396476 0.8648364893895729 0.8649833042328042 0.8653494880000001 0.8650487697245253 0.8632154663940711 0.8646485820499205 0.8655544114470842 0.8652764384288747 0.8653551569984035 0.864202399372713 0.8653709385801649 0.8638027669133193 0.8641497369833603 0.8649822056473094 0.8639013638757241 0.8636659705093834 0.8646383363946472 0.8651814771519659

GCA_023330605 0.9069302285864426 0.9444784059775841 0.9875917527932961 0.8232427680945347 0.9874510566829952 0.8654745252145922 1.0 0.925351340909091 0.98840193304288 0.9883959341923608 0.8343915020945543 0.8279653718285215 0.8652161182994457 0.9016022366710013 0.9439489069649213 0.8204145671546784 0.9449233936651584 0.821494510869565 0.9902936310769949 0.9231672613458528 0.8280391686114352 0.9875916670547148 0.906598899435608 0.9079987516391294 0.9241481219449448 0.9878017398608111 0.9874788958233317 0.9253556436637389 0.9886171254188607 0.9977724194594596 0.9885092265975005 0.997773334064007 0.9885120486767486 0.9874473114912068 0.9874189222118088 0.9443246617869763 0.9872686788048554 0.777587625 0.7735227844073191 0.987993045614035 0.9879856453529832 0.9889765192083819 0.9453804805260496 0.9440173918640576 0.9870037503024438 0.9874337054631829 0.9453175783404363 0.9877537613149119 0.9870614255014326 0.9873608227550541 0.9873351048292108 0.9900273845093084 0.987321853509185 0.9877576052631579 0.9874464929812041 0.9874257682198327 0.9899881483208955 0.9873527908078654 0.9899187353903693 0.9879175431544102

Citrobacter_europaeus 0.9089912455874938 0.927567736215691 0.92485181906852 0.8208723437949899 0.923390282000981 0.8628670083030616 0.9242424911302586 1.0 0.9230926773006136 0.9230926773006136 0.8339526572187778 0.8233860151471016 0.8630905080771235 0.8930570457482553 0.9272014229053049 0.8174982443690219 0.9268301804251112 0.8176778447761194 0.9237734371957157 0.934680880121396 0.8227085767682575 0.9248519970738844 0.9080566756756758 0.8952817861732203 0.9336394332909785 0.9240137530742746 0.9238663157894739 0.9913290811044004 0.923797703396042 0.9243938256048386 0.9234129376257546 0.9243743517816527 0.9233543011835811 0.9238963169587757 0.9244261418853256 0.9274791390067383 0.9232523937198068 0.7714137186742118 0.7714250808407438 0.9248127411793733 0.9243326178901313 0.923958505859375 0.9276167659681899 0.9278932090680102 0.9252019456790123 0.9247188970768851 0.9285690225750435 0.9239345599803344 0.9249173344904538 0.9248238108042043 0.9249211967694566 0.9234002642424243 0.9249491703377387 0.9247009637076999 0.9241154604280738 0.9246032644422311 0.9237786810492769 0.924173824412206 0.9236557952334632 0.9242179476617398

wang1_hy 0.9067104554812148 0.9423061660470881 0.9854830089306161 0.8205388190587549 0.989173220338983 0.8628925692108668 0.987033472381389 0.9237321010248902 1.0 0.9999360819486404 0.8347909332934491 0.824948148255814 0.8639190952130459 0.9003881650435677 0.9401094046740468 0.8194564155107189 0.9421283106939986 0.8208360130718955 0.9880738108047524 0.923089948992604 0.8260814694224234 0.9854832356308678 0.905284306315266 0.9059660449835734 0.9240271558059775 0.9838937692656391 0.9838323129251701 0.9245313662006378 0.9880422902547323 0.9872343511450382 0.9969515758754863 0.9875640877073392 0.9969247058823532 0.9854261474075796 0.9864360018111841 0.9409184678217821 0.985819194063927 0.7751453464947623 0.7750896451612904 0.9854800745573158 0.9879294316328907 0.989123542402827 0.9432643155510819 0.9415270849080372 0.9838421250291578 0.9873860438024231 0.9431856477832513 0.9851596116061228 0.9848148722157092 0.9848150616438356 0.9854715473441108 0.9869460135282566 0.9839608295036766 0.9868206953488371 0.9843235008103727 0.9837196548529071 0.9869958643617021 0.9834188542621449 0.9907133422818792 0.9878134414831982

wang9_hy 0.9067445419454453 0.9423098644652538 0.9852911136363637 0.8205315434083602 0.9890789332748025 0.8628155354371444 0.9870366811627375 0.9236964308367895 0.9996711194873729 1.0 0.8347404096889952 0.8252108159117304 0.8640519680293502 0.9003047309072271 0.9401279138991389 0.8194252442483455 0.9420812052358609 0.821829209672663 0.9881743148688047 0.9230497654258032 0.8262264326160815 0.9852913386363636 0.9052058868767056 0.905872927506946 0.9241320395701026 0.9840343950056754 0.984022697547684 0.9244758141245708 0.9877226959758084 0.9869731491836278 0.9967582638590459 0.9872539142273656 0.9967379273274566 0.9855460798338718 0.9862740530814216 0.9408487638512683 0.9856597299137541 0.7764436958266451 0.7762975090397751 0.9852488347260909 0.9877325447778554 0.9888870852610707 0.9432504272863568 0.94151034942182 0.9836576315789474 0.9873983782527881 0.9427767035942886 0.9851787517053207 0.9843806083916082 0.9845754865048764 0.9852073405231758 0.9866324880382774 0.9837410342465753 0.9865337368789363 0.9843853777061263 0.9838134790654638 0.98680011517165 0.983362034090909 0.990565227120161 0.9876178356481482

Citrobacter_koseri 0.8368686146788992 0.8356076696969695 0.8350488210652305 0.8331528284944626 0.8349496639418709 0.8356920928088506 0.8345981587684879 0.835415061983471 0.835049318455971 0.835066209288299 1.0 0.8362243405775529 0.8322950754830918 0.8348107130017975 0.8363678974358975 0.8344413356598984 0.8340035760171306 0.8313832453509298 0.8350016831091182 0.8369115980629539 0.8353523241317897 0.8350900179586951 0.8371102535899786 0.8350691153381642 0.8362237178702572 0.8347754205327746 0.8346514589574596 0.8364190890207714 0.8343192692422882 0.834607868753763 0.8338184538274607 0.8336975319148937 0.8337539768926725 0.8333244652567976 0.8344239453357102 0.8356546032707449 0.8344434939402896 0.7786746934632562 0.7786922168087699 0.8346939539060162 0.8346486734693876 0.8341126205450735 0.8342106594761171 0.8346936439370564 0.8337624544349939 0.834448484026522 0.8350089759759759 0.8345055518796993 0.8349910057733212 0.834744971445747 0.8347253663663665 0.8344599608551642 0.8348311895464104 0.8344817390002993 0.8378093457667258 0.8351058862876255 0.8340688187556358 0.8351736240193119 0.8342731257485032 0.8349083976083708

Citrobacter_amalonaticus 0.8259374962473733 0.8253895301418439 0.8284147200689062 0.9124813300492612 0.8241010983847283 0.8253103601953602 0.8275353066037736 0.8245004970930232 0.8258520152986172 0.8259886660777385 0.8361996456223943 1.0 0.8211867954345917 0.8238624926079244 0.8258204193932182 0.8467425835322197 0.8262735202398802 0.8472872940534643 0.8257067859237538 0.8255059848705267 0.9187143768400392 0.8284168733850129 0.8260811144042681 0.8248079657007688 0.8257805774896878 0.8250169258054981 0.824873522626442 0.8255364485981307 0.824899318985849 0.8262676467153284 0.8253081384388807 0.8252752856301533 0.825048202479339 0.8256467597765362 0.8251467981165391 0.8247511884144523 0.8250190397447055 0.77869624430956 0.7766421612284069 0.8261790521047985 0.8242800089445439 0.8235220443786984 0.8260555995167624 0.826135848603184 0.825221931884909 0.8261090802577623 0.8256589493933116 0.8256099941673958 0.8276919242068155 0.8272308781051416 0.8275109204052098 0.8262744385964912 0.8275746699478864 0.8254243610366399 0.8268033795749705 0.826242747510252 0.8244240307328606 0.8283929030390739 0.8243645695561513 0.8244298989898989

Citrobacter_murliniae 0.8630839797869435 0.8636498293067228 0.8628590954248365 0.8184053415061295 0.8629953333333334 0.8670407084910763 0.863265173878418 0.862639474909467 0.8628168560905026 0.8628106421052631 0.8311788192987715 0.8195008676599476 1.0 0.8640889668769716 0.863527622322137 0.8133663186813186 0.8627364223569736 0.8134048077514612 0.8621817563291141 0.8641155925925926 0.8198273099078341 0.8628583843137255 0.8619449449368789 0.8651905735528413 0.8656773112098248 0.8627158170280277 0.8626192936507937 0.8620769792207792 0.8623308496732025 0.8641429569327732 0.8634321682590235 0.8639497601476015 0.8633899601699416 0.8624834222107172 0.8622024434624384 0.8639022643008474 0.8624166285714285 0.7687362343938784 0.7687318445428916 0.8636323987538941 0.8627613970007894 0.863273998940678 0.8636996795550848 0.8641719353128313 0.8631235417761429 0.8621558980392157 0.8638177502606884 0.8630633412572637 0.8634004962845012 0.8629025065342394 0.8628763581699346 0.862293118251251 0.8629554272275936 0.8624876323413222 0.8626515895953756 0.8635818979645784 0.8621761688140941 0.8630321197668256 0.8624425883603509 0.8627873443656422

Citrobacter_pasteurii 0.8866476892001077 0.8934772192936216 0.9011150881168177 0.8220043834808257 0.9001363478935126 0.8649231205298014 0.9010843244639628 0.8943083196300102 0.9011266692170365 0.901055778458397 0.8345231655315369 0.8238661409198115 0.8661653212216958 1.0 0.8925518983675619 0.8197225356679637 0.8921265225366877 0.8183878051029819 0.9030023957032225 0.8953066657894736 0.8243361103089807 0.9011114979859014 0.8874218552390908 0.9470866916203816 0.8952293946540881 0.9011123899210994 0.9009890193581254 0.894607922901344 0.9022815425264217 0.9005450442017682 0.900372135770235 0.9000071297268908 0.9002406462585033 0.9007684354595682 0.9016945910828025 0.894075703145308 0.901816718000505 0.7712905141700404 0.7712938841636292 0.9008371406844106 0.9016041067813764 0.9002974395632961 0.8926183644112946 0.8929387886048988 0.9000822970550576 0.9011255145929339 0.8928579738058552 0.901214784273679 0.9013291255144033 0.9009669135802468 0.9011830573087604 0.9005458387013655 0.9010451718039414 0.9022267938931298 0.9022851686533098 0.9013127897539945 0.9008058311888831 0.9010388541935485 0.9005154934725849 0.9017097731283086

Citrobacter_portucalensis 0.9086212375719519 0.9824177153646448 0.9441694315373209 0.8221556173393124 0.9424778555528075 0.8644532897435899 0.9425487737041721 0.9278755694956949 0.940587140406763 0.9407074650649667 0.8361091808669654 0.825032067547724 0.8650280734908137 0.8922285424354243 1.0 0.8210471683913453 0.9829482223796033 0.8201081361035831 0.9436818026706232 0.9251274279835391 0.825256626506024 0.9442283382789318 0.9065882488014131 0.8967202008756117 0.9244816976744186 0.942357762444554 0.9422313165680476 0.9284745353709637 0.9442691197799451 0.9428795299469295 0.9422353908836262 0.9426453226217801 0.9421674528301887 0.9434083434038267 0.9444115100421523 0.9744452894491129 0.9430562747524752 0.7749837194388777 0.7750088527878058 0.9452427765180147 0.9447526018495377 0.9443605493133583 0.9864190633151529 0.9974452841918295 0.9441059752712592 0.944979015777611 0.9761454299065419 0.9438835548917102 0.9442543213925326 0.9442176457454727 0.9443075663607046 0.9436013697955161 0.9442756206554122 0.9442811247803163 0.9441763084832906 0.9443425828658846 0.9440695181321412 0.9428108445346183 0.9440473541147134 0.9447102615193028

Citrobacter_rodentium 0.8216944493952896 0.8244093052428434 0.8209023514127963 0.8432858461084907 0.8204710714285715 0.8223632468780019 0.8198851635663887 0.8197095811355881 0.8192855369551485 0.8193039304897315 0.8354649568827851 0.8474592541353383 0.8153890708252112 0.8197590329957529 0.8218148253968254 1.0 0.8201281346469622 0.8656909199224163 0.8208909105534106 0.821883947368421 0.8454837915808068 0.820894294996751 0.8213716708542714 0.8186835879332478 0.8207461798469388 0.8214302573174653 0.8214029761904762 0.820153682254568 0.8204483824471492 0.8193197260273972 0.8188969741461857 0.8189589499192246 0.8186386464000001 0.820103177356021 0.8193496168461052 0.820831388530928 0.8208595750160979 0.7788087821543408 0.7788065956591641 0.8209745021221025 0.8211228349514563 0.820332907460775 0.8205066237198547 0.8202052657480315 0.8197826308857425 0.8211474300585557 0.8205597395171538 0.8211646312399357 0.8211914916035561 0.8208557621059472 0.8208251384815901 0.8196535842408713 0.8209042936197917 0.820706273752013 0.8197889958821667 0.8206336097717776 0.8183555450535601 0.8197477249683143 0.8202219673704414 0.8212386073500967

GCA_019795045 0.9089432612055641 0.9901416214382632 0.94624579946459 0.8222782476076556 0.9436098343373495 0.8642924801026958 0.9443590680733484 0.9278485604748948 0.9430530843433096 0.9431143490099011 0.8342777626811594 0.8262986252241483 0.8634336507517806 0.8925993861959957 0.9824247025628967 0.8201835619540602 1.0 0.8211270446944362 0.9441593084845512 0.9239025983648443 0.8257533981154299 0.9462454027743976 0.9076534905897116 0.8964383095670211 0.9248442470260693 0.9444498994722292 0.9443597587333501 0.9273646896035901 0.9446138569636134 0.9436356834712817 0.9435521974118244 0.9436771378358022 0.9434609728219455 0.9442570864567716 0.9443266566641662 0.9743606216739236 0.9451758337372758 0.7726313751017088 0.7726327949552482 0.9446116156335574 0.9439386489176411 0.9438360924369747 0.9825157389105953 0.9822176501305482 0.9460383002694098 0.9446252288072019 0.9745496601711028 0.9442038318695107 0.9461640024752476 0.9463347714007783 0.9464371599219894 0.945156406968304 0.9464664820687974 0.9454223995932894 0.9455108747464505 0.9463050351229302 0.9436500962012828 0.9445745816430019 0.9443968672302337 0.9439219105590063

Citrobacter_sedlakii 0.8206020661157025 0.8209973331310679 0.8230849199153822 0.8409983365785815 0.8205243239987956 0.8197508072437077 0.8207184788560997 0.8182988550295858 0.8214427324535093 0.821954085055406 0.8315680754265189 0.847075306957708 0.8154029131912265 0.8181530452292443 0.8196902147610405 0.8656471223418946 0.8204705287286064 1.0 0.8200595121221192 0.8196413172205439 0.8451421216290589 0.823080528860683 0.8211882361824223 0.8182050247066092 0.8204490178301602 0.8212746225563909 0.8213376812030075 0.8186051981076287 0.8201002988228194 0.8218806995515696 0.8230357253731343 0.8201359648591335 0.823001613385121 0.8210023065157541 0.8205426682764865 0.8195973884197827 0.8256280427251733 0.7795594732765299 0.7795133875968994 0.826097283840191 0.8190287025220299 0.8195440765290751 0.8193589512646515 0.819104426380368 0.8220681557251908 0.820661862089915 0.8189223667570009 0.8215738102409639 0.8228606574500767 0.8227942185128985 0.8227241267434809 0.8200788348928464 0.8227603818181818 0.8203025474006116 0.8225107462686567 0.821753035495716 0.8188712579762989 0.8230316309272123 0.819363164901664 0.8192768239564429

Citrobacter_freundii 0.9059124358648718 0.9421585178875639 0.9879010339293902 0.8222197358266549 0.9884886289959478 0.8648893718921749 0.9894036622654621 0.9242459056831923 0.9882086618769647 0.9882041580601706 0.834496033717835 0.8253434285714285 0.862979652357124 0.9024588316237176 0.9431281672422298 0.8202788366179276 0.943648931372549 0.8200878873659119 1.0 0.9227042555306986 0.8250675130434784 0.9879040591336237 0.9054935613682092 0.9071164705882354 0.9228751834170854 0.9869018985876362 0.9868553580533025 0.925361357265798 0.9886750584728273 0.9892555242027988 0.987128226363009 0.9893017811704836 0.9870800644418872 0.9873610094929381 0.9884785373134329 0.9428568110236221 0.9888974910233393 0.7733899919257167 0.7734339135702745 0.9881002964519141 0.9881391632837168 0.9881384711111112 0.9436859244532804 0.943387729985082 0.9878591153127917 0.9881298062558357 0.9435589621249386 0.9865452949340737 0.9871311162899091 0.9875867424764531 0.9879403240634338 0.9892241377787591 0.9878027124483234 0.9881240708789928 0.986998723805039 0.9879171364175564 0.9901881830065361 0.9876479498806683 0.9892822217294901 0.9879831899641578

Citrobacter_braakii 0.9094374288643937 0.9239975725190839 0.9236642799799298 0.8235608868144693 0.9230721313980137 0.8671801011984022 0.9230752247336971 0.9352625631407853 0.923347962302598 0.9233208125318391 0.8368557818620067 0.8258984128360798 0.8660449377813079 0.8959485937086968 0.9246206111967128 0.8215949556962024 0.9233987757699161 0.8205673037439614 0.9230484109589041 1.0 0.826624445414847 0.9236650777722027 0.9090622324159022 0.8974283762480295 0.9874410002353494 0.9235225506072874 0.9234299392251204 0.9352042525900346 0.9234196330043026 0.9231642029358743 0.9230166361735495 0.9227740581969343 0.9229483124018839 0.9223382008688986 0.9235556610425586 0.9250960515356585 0.9234197809667676 0.7734678485576924 0.773470140224359 0.9230269493251847 0.9233359873896594 0.9229843220125787 0.9238934871661915 0.92434788750648 0.9232142875350497 0.9230617882653062 0.9243365763231911 0.9233967914438502 0.9234338221768014 0.9236014379249561 0.9236183110999245 0.9226967943548389 0.9236095118268748 0.9236021680286006 0.9234134192218502 0.92389119078105 0.9229196032144651 0.9233390974729243 0.9229615869017633 0.9234066733668342

Citrobacter_telavivensis 0.824496047465573 0.8255538308886974 0.8256647114845939 0.9347225200826256 0.8240638465974626 0.8231230444829578 0.826415533239038 0.8226415783602901 0.8259559327638466 0.826320995862069 0.8339194239631337 0.9157739491807172 0.8197208178752107 0.8227335768688293 0.8245572112917025 0.8446318608695653 0.8245340993434199 0.8436838728790735 0.8241987650856021 0.8245341149943631 1.0 0.8256920117680022 0.8250982857142859 0.8224772338593974 0.823938111551529 0.8240168192090397 0.8239244673636621 0.8233350165471595 0.8225070775740478 0.8267250532809871 0.8317580980926431 0.8261679569892474 0.8314708049113233 0.830598039534254 0.8242445508307519 0.8247723119054483 0.8240337869492472 0.7753435910976991 0.7752952095130238 0.8236064934696196 0.8230218654785858 0.8314426187208344 0.8247348697916667 0.8239179721496953 0.832209385368507 0.8243903373015874 0.8241725342075258 0.8242242533936651 0.8258282371428572 0.8249897117829896 0.8250163020685747 0.8313048151191454 0.8251100283045572 0.8244658432708688 0.824699889073766 0.8259417931609674 0.82326443019943 0.8255628258426966 0.8235285257702276 0.8232857359679265

GCA_020639355 0.9065719823232324 0.9451531773925435 0.9999980729672036 0.8219522599704578 0.985560098737084 0.8648919226722777 0.9867544612873134 0.9255284752714112 0.9859538742857143 0.985704935271406 0.8349734719334719 0.8284665150214593 0.8634418111053451 0.9005873674956041 0.9446545833333333 0.820858344072165 0.9457949220652704 0.8228807627879151 0.9879039519450801 0.9232554741055792 0.8260306275303644 1.0 0.9054271488729255 0.9068535524999999 0.9236411240984831 0.9875241064552661 0.9874996103307657 0.9254625358619012 0.9884165276497697 0.9869237256740914 0.9863991597238753 0.9870806957967229 0.9863293715781957 0.9880023181919892 0.9884113863585868 0.9450761203268135 0.9867915271593946 0.7798262680734662 0.7759090728213291 0.9887289674994472 0.9879998112349329 0.9864398095456633 0.9455251646743109 0.9449459800249688 0.9991024659543264 0.988804414612676 0.9450106886517944 0.9884358004953839 0.9993888516550878 0.9993488521949256 0.9997622075055189 0.9855191883992807 0.9997615114411883 0.988251298642534 0.9883989879086431 0.9882331098779135 0.9877663708362451 0.9880869023420239 0.9873586538461538 0.9878764039855074

Citrobacter_werkmanii 0.9917133922018349 0.9074290813779331 0.9050804015865147 0.8218952799535828 0.905457052737825 0.8631007063784923 0.905568452289588 0.9085529393049439 0.9055359641701916 0.9056779328358211 0.836479460355743 0.8261958045467965 0.8631512587038028 0.8869591192660551 0.9061937707808565 0.821339458372021 0.9069684887218046 0.8213352473763118 0.905291589089089 0.9082786452762923 0.825951467057101 0.9050963717472119 1.0 0.8872350193548387 0.9072821353228712 0.9054541116116116 0.9054114117647061 0.9094196403402188 0.9058274968490043 0.9049559841917391 0.9056384949832778 0.9044263297325101 0.9055598712667354 0.904576097806907 0.9055055839325564 0.9069941565349542 0.9079204773502191 0.7730183005507475 0.7729867453758362 0.9059734167298459 0.905554441919192 0.9056794761904763 0.9063495764225568 0.9063130656370656 0.9044588227911647 0.905442142316427 0.9053037693459811 0.9054538944723619 0.9051918851214574 0.9052029858173674 0.9050512397311427 0.9054484913151366 0.9048211868186323 0.9060645678092399 0.9055533569261881 0.9056312893477716 0.9049208013062046 0.9052868795758647 0.9054149336338593 0.9055602613065327

Citrobacter_youngae 0.8881121874184286 0.8976023036785808 0.9079242305751767 0.8211448039215686 0.9069430572094227 0.8653245240641713 0.9083525375494071 0.8968835668626952 0.9071362726576462 0.9071357518509063 0.8349694041916169 0.8255097756126366 0.8670251220806795 0.948200496350365 0.8978091546018615 0.8188267901234568 0.8967953736006249 0.8188132008626002 0.9080073371104815 0.8977947077409162 0.8239923099587507 0.9079257467204843 0.8880421172217918 1.0 0.8972410727464418 0.9079493992395438 0.9078428466413182 0.8972306287878788 0.9080333531848784 0.9088263960447567 0.9073761264308012 0.9081923172014729 0.9073864147993745 0.9083288348271447 0.9076751327433629 0.8977494162105812 0.9083859835009024 0.7721851556813587 0.7721847634452084 0.907926454755191 0.9080587682832948 0.9077970266081115 0.8973436459430981 0.8972402274509804 0.9068836045314109 0.9083257860824743 0.8973529162410623 0.9085117740286299 0.9080342638672887 0.9075437591888468 0.9076654049923587 0.907563995869902 0.9076836389101095 0.9089740883697308 0.9095584805293969 0.9096764305804144 0.9079271121351766 0.9090220249936242 0.9078545324675326 0.9081799872090048

GCA_001273815 0.9072267171457905 0.9237495913281835 0.9231614096267191 0.8215183963911525 0.9207294129255057 0.8641345408838317 0.9225863469746421 0.9334722020854023 0.922955431014011 0.9230312384560739 0.8343363668957275 0.8251135875216639 0.8655608644501279 0.8944980417956656 0.9230304645227158 0.8192525562499999 0.9239925204867148 0.8196782607407406 0.9220050444883837 0.9865537076566124 0.8244843850114418 0.9231452001965118 0.9057983665238216 0.8957663827032625 1.0 0.9226587596899225 0.922480875 0.9332891218905474 0.9234828459080855 0.924089216011369 0.9240253541416568 0.9241152377555872 0.9239168523409365 0.9245058000972763 0.9196666885325558 0.9238296766683911 0.9225362154898608 0.778047230011368 0.7741927216653816 0.9201577451946773 0.9229466799501869 0.9213474920088518 0.9239569330346029 0.9252548577134299 0.9212445684779935 0.9235778153542336 0.9232814208984373 0.9235975779906658 0.9224828209585297 0.922975227328582 0.9225787822244046 0.923106899542279 0.9205831239347455 0.9224214535901928 0.9206200314161431 0.9218470443227093 0.9182940748898679 0.9220936852394919 0.9216923596339353 0.9229228425764735

GCA_001306025 0.9070408508430611 0.9446257743307481 0.9885112743525097 0.8219072036926742 0.9868914846455783 0.865492624567013 0.988037113752122 0.9254904907521578 0.9862454400554401 0.9863355663655317 0.8341380035810206 0.8243948810939358 0.8636902698370292 0.9014705902061857 0.9432553201600803 0.8204638602116063 0.9449622132185362 0.8205556071964017 0.9878528664646936 0.9237450982393469 0.8244881571175431 0.9885137680495072 0.9061793678598629 0.9075589880498347 0.9239830565552698 1.0 0.9998391319807571 0.9264180959446092 0.9895983423085928 0.9879727445783134 0.986369607142857 0.9879687925170068 0.986321588361555 0.9884617121702916 0.988784729481889 0.9445178321149484 0.9885302533978346 0.777414804313099 0.7774473780975218 0.9895523415533534 0.9894287374562428 0.9869800139534884 0.9453917060895823 0.9442546190837763 0.9885093853704136 0.9899678231917336 0.9457897426286858 0.9986264721085506 0.9879394192037471 0.9884002466005991 0.9885536604469016 0.9868119246861925 0.9885337563334868 0.9893103375430541 0.989156669005848 0.9891661238854996 0.988699520692074 0.9884141957229196 0.9872869745370371 0.9892416573686658

GCA_001411885 0.9067636948102248 0.9441005565217391 0.9880268403015764 0.8221207359050446 0.9867578158803224 0.8652087768069896 0.987458389277952 0.9251436624907885 0.9862467664670659 0.9862797644341801 0.8347426480214221 0.8250314281498967 0.8635882535062186 0.9011827592402465 0.9432504708520179 0.8208544210526316 0.9446401590106007 0.8215434353435345 0.9878079394644936 0.923589010708822 0.8251480518715002 0.9880271236006397 0.9060094767441861 0.9072189435013934 0.9237422887864825 0.9998925982441472 1.0 0.9260077000738735 0.98939183663944 0.9877653374969972 0.9863987811831788 0.9878547481840194 0.9863665064255117 0.9882172390650312 0.9884937817433082 0.9442797437185929 0.9884284594159577 0.7773669218626676 0.7773647829518548 0.9891699838895283 0.9891949232201025 0.986985515404216 0.94471745 0.9441935048150025 0.9879869082013049 0.9896587052341598 0.9456099203782035 0.9988579440258343 0.9873469732246798 0.9879892403029608 0.9880957018348625 0.9867145298749422 0.9880984193622391 0.9887543927589368 0.9887312803171642 0.9889600889096865 0.9885217844646605 0.9879683692878682 0.9873320716763005 0.9890837099767983

GCA_001922445 0.9097939176559737 0.9277456377991612 0.9253600808229244 0.8225275525866971 0.9246036920039487 0.8632017474226804 0.9247787201408098 0.9918411909385114 0.9246538397040691 0.9246986704489392 0.835336373270533 0.8255113013100436 0.8635338523524824 0.8946559907715971 0.9282629677419354 0.8199746648216483 0.9266962012987012 0.8186418677734955 0.9253973256100566 0.9351907671232877 0.824470978601997 0.9253725073457395 0.9093539454456893 0.8964207626045096 0.9343647682957711 0.9254842087127739 0.9254390273331692 1.0 0.9252652228207641 0.9252134905422446 0.9246326215805472 0.9248273790117167 0.9245421444866923 0.9247416928838952 0.9252256389698738 0.9294143341645886 0.9253165780570324 0.7736575088827478 0.7736575759968417 0.9256304426310584 0.9252482127972543 0.9252362830328476 0.9273891399747793 0.9279352555668017 0.9249853409942543 0.9255536044499383 0.9284872563087581 0.9255938155291792 0.9252412945871789 0.9251913643067847 0.9253949286417322 0.9248894512493875 0.9253903542435424 0.9251974750182792 0.9252894382301764 0.9249640064741037 0.9250092442146727 0.9249625086934924 0.9247197776130467 0.9252430662106035

GCA_002189125 0.9065846899922818 0.9443229926289928 0.9892125338535691 0.8217264484068986 0.9889070016282857 0.8652412450119714 0.9884647984736465 0.9254171609139524 0.9886261200279134 0.9886516453339539 0.8341821874076264 0.8240412153082092 0.8635353468961919 0.9023415262361252 0.9450853257861636 0.8197615030187482 0.9447823586550436 0.8200884825721155 0.9894663792314731 0.9238323717623158 0.8237035632183907 0.9892146145020653 0.9061434853830644 0.9075019253539255 0.924583245614035 0.9896193671766343 0.9895861261888194 0.9263356457925636 1.0 0.9882648380231733 0.9877193665480427 0.988454965599051 0.9876945339990489 0.9889138937043797 0.9883476650563607 0.9453023312421582 0.990479376401974 0.7743287890625001 0.7743550938232994 0.9888241116279071 0.9909833181507629 0.9885923290203327 0.946195933268859 0.9450187290289782 0.989083724042455 0.9890522063893358 0.9474994562146892 0.9897559772516249 0.9886172780531771 0.9893728288868445 0.9891949954022988 0.9887662044134727 0.98922966858458 0.9889152882380288 0.9886365185185185 0.9892742853762423 0.9896813916455114 0.9887826338127801 0.9891118173388966 0.9909166385789115

GCA_002215385 0.9060850938967135 0.9433447962690232 0.9865194521819869 0.8211514378891195 0.9887031785465523 0.8648929984051037 0.9956832955759778 0.924729220355979 0.9868322644444446 0.9866495317354638 0.834612361944778 0.8262016964800925 0.8657051423348133 0.9000770646249674 0.9433660340015224 0.8195861300605674 0.9435009937421778 0.8223219769162475 0.9890058028362306 0.9225432964202936 0.8274217780979827 0.9865199419684308 0.904832998979071 0.9080132538860104 0.9245243642611685 0.9874671663097787 0.9871036030461686 0.9252761277344732 0.9876250765606597 1.0 0.9876105100700526 0.9998436952061183 0.9876866133975482 0.9860713434969033 0.9847779707160832 0.9421156073127973 0.9851868920467568 0.7766276895593489 0.7767575516693164 0.9838604439252338 0.986923566400751 0.9884235180118559 0.9435279979929755 0.942229130100077 0.9861594823529412 0.9865768882031806 0.943227913561848 0.9863015890926187 0.9857828241732096 0.9850076425233646 0.9862506672932331 0.9888269123369192 0.9848524713851905 0.9871926467094322 0.9843468828744751 0.9861978795688847 0.9867984708020905 0.9847526111111112 0.9884275097633816 0.9868500957943924

GCA_002252025 0.906076173455979 0.9418381597222222 0.9851579938995775 0.822540608128152 0.988585572519084 0.8645090661070305 0.9877407416939634 0.9236595114656032 0.9953106231454006 0.9957483082467671 0.8328982872596155 0.8247461700288184 0.8642708985507246 0.8997308700182339 0.9412236156186614 0.8185469977924944 0.9419725411471322 0.8227964421669107 0.9866267671921406 0.922619908496732 0.8325913961218836 0.9851590450492727 0.905033724137931 0.9067451759834368 0.9234205071196603 0.9850905802526907 0.9849371472033701 0.9244812434061794 0.9867024167257266 0.9872534303215926 1.0 0.9872883796803151 0.9998759414298019 0.9856424558991981 0.9841435627991793 0.9434564352436254 0.9841903015764222 0.7744122261208577 0.7742683294301327 0.983197243394903 0.9858219253484527 0.9889445802841154 0.9423151154039137 0.9418611006128704 0.9856056494215237 0.9859379030732861 0.9425541844823445 0.9848484133849306 0.9840241911764708 0.9842535423490876 0.9845970905678086 0.989644862385321 0.9829348221343874 0.9849710632792286 0.9835450578435909 0.9859396935407827 0.985309381834412 0.983589765463325 0.9889829750566892 0.9857722926484448

GCA_002252065 0.9053683111806099 0.9421500541338582 0.9862987327887982 0.8218904420866491 0.9886407799120575 0.8634494370685076 0.9956392518756699 0.9241846774595267 0.9866925987062236 0.9867986303814411 0.8333238898862958 0.8247145782784518 0.8647203506017792 0.8990745435684647 0.9430031414807303 0.8183046759405628 0.9427771421483503 0.8209786490498815 0.9888958697142858 0.9219337461300309 0.82635343069874 0.9862994375729288 0.9040421599592461 0.9071664403717088 0.9239148047454276 0.9870742731172546 0.9870097281831187 0.9248514149523331 0.9875376310223267 0.9999381240795289 0.987469197907585 1.0 0.9873775992150021 0.9855645200945626 0.984290124137931 0.9419841643559366 0.9849316636071593 0.7723108263806119 0.7725153065286624 0.983625173627405 0.9864652589641435 0.9888402947224126 0.9428472825 0.9421302771421307 0.9857522563261482 0.9864879455621303 0.9426229635145198 0.9857804854596622 0.985462488687783 0.9848987025761125 0.9859487573546717 0.9888278702010969 0.9846552611852893 0.9870000284562486 0.983923969767442 0.9853464116279068 0.9865291862567811 0.9845722462253192 0.9884284722858452 0.9864758249473439

GCA_002252125 0.9063190853979969 0.9419495942245457 0.9855198800846461 0.8222886119402985 0.9888658857913669 0.8647183850604945 0.9884774154929576 0.9239664785214785 0.9952979949077022 0.9954580626720305 0.8329062488701418 0.8247145076967762 0.8642923876255951 0.8999958206607235 0.9413535107731305 0.8185220190174326 0.9420711852963242 0.8228920411764706 0.9868361568358482 0.9227921479150275 0.8321046917712692 0.9855208323536327 0.9052310199386503 0.9070585480093678 0.9235367702330244 0.9853562978623444 0.9852912335526317 0.9249522583901084 0.9868110628247521 0.9874320113935146 0.999959860296654 0.9875595786701777 1.0 0.9859142719559735 0.9845944309594689 0.9434362746585735 0.98461379231473 0.773997374411303 0.7737958035363458 0.9836486306179775 0.9861591391984824 0.9893275873974967 0.9426246225465527 0.942428923824131 0.9859282213957558 0.9862786344238975 0.943030369654506 0.9850853837368662 0.9843004442860537 0.9845834130332864 0.9849704650070455 0.9898715066039848 0.9833534684369905 0.9854789686098656 0.9837273040241917 0.9862139389638042 0.9856542563184971 0.9835855278930383 0.9894211446740859 0.9861032939229132

GCA_002738435 0.9052225226156629 0.9435825196463655 0.9878837190635451 0.8217265592669227 0.9864432919981283 0.8638881138600693 0.9865812674362674 0.9243267777505514 0.9858301415313224 0.9858888111726686 0.8331979514824798 0.8259565205959685 0.8628766692627207 0.9005830186304129 0.9433805835010061 0.8196069075144509 0.943456100271672 0.8213923472474289 0.9875159321642825 0.922457942229039 0.831550881702598 0.9878852630405708 0.9046865878208354 0.9080517563089473 0.9252290594675293 0.9879721546581 0.9878633448038541 0.9247083415719228 0.988162882554162 0.9862941688591245 0.9865329863520702 0.9861929858614904 0.9865227701920851 1.0 0.987801805936073 0.9438805211480363 0.9877151299589605 0.7740432974051897 0.7735246517213772 0.9906957862767155 0.9883374537987679 0.9870722262361707 0.9443242608695652 0.9441743457473578 0.9880119481368489 0.9956416447656592 0.9437702153465347 0.9886635280641467 0.9873088379482523 0.9877143888888889 0.9879114648829432 0.9874013577439164 0.9878686290682123 0.9888080078485687 0.9882005922089618 0.9883620648424927 0.9870610972451143 0.9868672390266092 0.9872869950968947 0.9882642967859586

GCA_004103775 0.9061099898167008 0.9440696509388542 0.9881943206582168 0.8223602775343265 0.9874868365276213 0.8645167961418144 0.9862147793602616 0.9244728485576924 0.9858184650011255 0.9859846507972154 0.8337573329408301 0.8257014619883042 0.8630550012909889 0.9008873323170731 0.9435010544554454 0.8190140050141023 0.9434778763040238 0.8207630343166767 0.9885594304088194 0.922811824120603 0.8249106033977738 0.988224129032258 0.9056131043005438 0.906430658652642 0.921868040439341 0.9878948168373151 0.9877587209831588 0.924779589570256 0.9876540323325635 0.9846181178094799 0.9848389427516159 0.9848140321453529 0.9847538346038346 0.9872417364447494 1.0 0.9441851497895519 0.9867817190305206 0.7755056208382296 0.7755078300039171 0.9854633844073908 0.9884875522868306 0.987971362391602 0.9443843885601578 0.9432646010571355 0.9883722222222224 0.9877358498745153 0.9435771780028944 0.9878968514715948 0.9881774880546076 0.9885017270504557 0.9884768968589885 0.9879544190260475 0.9885599492945327 0.9874737887067396 0.9855388318898513 0.9863886951605609 0.9877663084961381 0.9868013775510204 0.9883149293849657 0.9884079803293687

GCA_009856695 0.9082320430393197 0.9744959683225343 0.9454925692883894 0.8232847338345864 0.9432803152585121 0.8645209234828497 0.943416565836299 0.9282885778443114 0.9422698077883176 0.9423355594318465 0.8358596043491392 0.8248771929824562 0.8652825172780436 0.8936423404811715 0.9752952773859898 0.8206105840821566 0.9745956364078852 0.819667685774947 0.9437638168698681 0.9254344076700813 0.8258454248952722 0.9456073469897578 0.9075588049029624 0.8974651620491375 0.9249092866613632 0.9445284799596673 0.9444616750756812 0.9294686198305928 0.9448677879249112 0.9432036697247707 0.9439151655969192 0.9430138511076765 0.9437832707636925 0.9444742612474438 0.9452943864491843 1.0 0.9442560173913043 0.7742856637168142 0.7743108128772638 0.9456944337957124 0.944837101596149 0.9443681958893682 0.9763728984088128 0.9759160377815451 0.9448386083397289 0.9452542991366175 0.992194746703678 0.9446992031171444 0.945204246680286 0.9449221473314958 0.9455089841986455 0.9437489276745353 0.9455621179422835 0.9452841418764303 0.9447365807117072 0.9448383687943261 0.9442418250188017 0.9440748756976154 0.9434871121235129 0.944727382753404

GCA_009857035 0.9066902804691483 0.9422999591247897 0.9870291016495765 0.8219977761457108 0.9867215437471372 0.8649075796681592 0.9864801867849639 0.9234119149447382 0.9852026844262295 0.9854707408246489 0.8340657605177996 0.8245913919308357 0.8634281888745149 0.9009710068130204 0.942245379088206 0.8199710548119822 0.9440739603001694 0.8255830786904079 0.9887371800628648 0.9235880278128953 0.8250808124459809 0.9870286465997771 0.9085551597950721 0.9072218416347383 0.9243486361373817 0.9877318566849818 0.987707192580719 0.925448018300024 0.990071915562542 0.9850284223801881 0.9847229866236162 0.9850898679638639 0.9846994034682081 0.9877322663925062 0.9870978981606102 0.9434588500369366 1.0 0.7746260365369341 0.7745324592769169 0.988258505416759 0.9897856580427447 0.9866698680618745 0.9435467538614849 0.943146213814119 0.9868412160620581 0.9882709798994975 0.9426243740831295 0.9874588479157125 0.9866275466545288 0.987263633714414 0.9868466039847773 0.9860942777155655 0.9866718234636872 0.9876713600917431 0.9861759298569451 0.987773171581147 0.9889476179621609 0.9864856500691563 0.9869333874285715 0.9897217334826428

GCA_011077325 0.7719708319672131 0.7734901821125051 0.778191320754717 0.771649301260023 0.7743631626984128 0.7710643400809717 0.775938423782921 0.7711998636181308 0.7732128565689033 0.7735482771084339 0.7770683906186817 0.7761589874857794 0.7682290714574083 0.7699850265848671 0.7725650141185962 0.777368826405868 0.7715397914110429 0.7773561254901961 0.7711608612244899 0.7714573479318735 0.7744925213343677 0.7781155110062894 0.771490523138833 0.7700981781211874 0.7772618349705306 0.7759316466826539 0.775566188951161 0.771994938467646 0.7724222814755413 0.7728982184602983 0.773891924 0.7722628276699028 0.7737612164865947 0.7739441512469831 0.7741090666134822 0.7728944848484849 0.7732194962015194 1.0 0.9999253470031545 0.7771339935846031 0.7718211572942135 0.7717154409987919 0.7763231222267042 0.7756838315874295 0.7740648367593711 0.7785687085308058 0.7790300663285215 0.779488427895981 0.7763335060240963 0.7779084743083002 0.7773749286846275 0.7748711797528897 0.7772824307205067 0.7724076030719483 0.7724065027977619 0.7732318163751987 0.7707952429149798 0.7781124705882354 0.7746996670677898 0.7717664306064306

GCA_011077955 0.7721419088669952 0.7735325752644426 0.7737718895116094 0.7720100153433065 0.77425603117506 0.7710013950667205 0.7714206902151847 0.7715285467683661 0.7724739188102894 0.7724735156878519 0.7774638514610389 0.7741232693046486 0.7684141696969696 0.7695414589293011 0.7729110701256587 0.7772139024390243 0.7718248665297742 0.7774914105594957 0.7709823500611995 0.7718653955954323 0.7742080598755833 0.7737852420968386 0.7717460442655936 0.7700049959349593 0.7739206247496996 0.7755915231259968 0.7753616373801917 0.7723238522637015 0.7720408219727345 0.7722205927419354 0.7731973104549081 0.7717242313915859 0.7730741653354634 0.7727911771795902 0.7732428951548848 0.7726339265536722 0.7727773728474169 0.9999864208389717 1.0 0.772649511002445 0.7712908580723873 0.7714310800970874 0.7721022084878452 0.7731354004106777 0.7738512374042725 0.7739774108216433 0.7747650218167393 0.7752875029964044 0.772660431771894 0.7737901287208367 0.7737387771520514 0.7704638392857142 0.7737172054684359 0.7719622974607013 0.7718173596176823 0.7724679385964913 0.7705131083265967 0.7738496421471173 0.774249599839936 0.7712083462007314

GCA_013371705 0.9064299610692966 0.9444360084033613 0.9892955215268531 0.8222739490254871 0.9867253498712849 0.865677323300188 0.9875021257625528 0.925932884851461 0.9854432102803738 0.9855740948376547 0.8350152554961379 0.8260810815551008 0.8649184577501297 0.9005176569250317 0.945169597977244 0.8208211540941329 0.9440465870052277 0.825897546556311 0.9882415284249767 0.9229392081736909 0.8250734703995335 0.9892959476253883 0.9059324383422325 0.9072924527820316 0.9226541647655259 0.9894574793577983 0.9894302019274898 0.9259280414917263 0.9887316313213704 0.9851318605200946 0.9849339235699027 0.9852248219832735 0.9849461739543727 0.991073793571752 0.9862915971907568 0.9453431193353475 0.9886325785745906 0.7786487692916503 0.7750005711987127 1.0 0.9891237136929462 0.9868472710323934 0.9456504377188594 0.9444217667934094 0.9877152250622032 0.992486461916462 0.9451173979340876 0.9894551574623459 0.988781503164557 0.9891787708009763 0.989245263509006 0.987564527996298 0.988142436266903 0.9887726752470697 0.9868343722441401 0.9880357306255836 0.9867848508853682 0.986714137694419 0.9882330543146014 0.9890595128676469

GCA_014218795 0.9064282127221224 0.9445326012422359 0.9890428525567531 0.8215499254399046 0.9891090923862582 0.8653360991207035 0.9881396851718337 0.925550189182634 0.9883798874032372 0.9884474636320977 0.8339709391932572 0.8234232355582161 0.8635035003977726 0.9009072132822478 0.944827100681302 0.8191307029990326 0.9443118865107017 0.8183218598959927 0.9892247877412031 0.9234615577119509 0.8237294931831655 0.9891130252293577 0.905652105263158 0.9073061583954745 0.9239987925300589 0.9896446236559139 0.9895675040897408 0.9258137579931137 0.99141119825207 0.9879155589988081 0.9868961218637994 0.987989675417661 0.9869050896914615 0.9891923140495866 0.9892131603336424 0.9450342882472138 0.9907724245860741 0.7723295776957769 0.7722502503077554 0.9891783677254812 1.0 0.9879885054285055 0.9448756046976512 0.944796068548387 0.9891763625839315 0.989532543698252 0.9450936695652173 0.9892281674049896 0.9887359450011652 0.9890485528120714 0.9892759674087676 0.9882048641745996 0.9893078691159586 0.9888530072210575 0.9889181466540103 0.989304636342099 0.9896472851201479 0.9883610934157356 0.9890405095688266 0.9999656117622867

GCA_015731115 0.9064764249363867 0.9427453519417475 0.9862084238258877 0.821502432033097 0.9911827323131515 0.864299628661088 0.9884006258724988 0.9238674309258361 0.9890752777777779 0.9891018663136997 0.8331344905885868 0.8228870593445529 0.8636813679493938 0.8992809501435657 0.9435094322161081 0.81883966984127 0.9427691892551893 0.8185581924111145 0.987824819490587 0.9224526747413576 0.8319240500985083 0.9862585701191567 0.9055419808708786 0.906295758354756 0.9218986435568952 0.9856022794959909 0.9855316219931273 0.9253065064102564 0.9878604373987503 0.9889031084503799 0.9888248541171386 0.9889963325635104 0.9887905790838376 0.9870480883678991 0.9878520613636365 0.9433430608651913 0.9871186416382253 0.7710103397866456 0.7713012644135188 0.9857283491989784 0.9865981277080959 1.0 0.9425731362530412 0.9431287965043695 0.9876329988876531 0.9863349619728048 0.9428583460309657 0.9841351643927533 0.9855695774320873 0.9855828900022775 0.9861186732355637 0.9904630534351145 0.9861433356258598 0.9876539841933984 0.9862853158018314 0.9864771485849056 0.9894109689662871 0.9858063313748531 0.9911101818978741 0.9865062104304257

GCA_015893905 0.9076617490297543 0.9830370873124147 0.9459791549295775 0.8223368888224745 0.9439802764864205 0.8649038568845258 0.9445979935435809 0.9281686694747275 0.9436586717594875 0.9436618979892104 0.834674256285974 0.8263587511124297 0.8642423540241977 0.8920086149003149 0.9856057358753779 0.8201030558244595 0.9823289120580236 0.8204351976885644 0.9440383761310834 0.9235885553854009 0.8252779021803183 0.9459663705826171 0.9065948741821842 0.8959475593395254 0.9245311535580525 0.9446935504407443 0.9446330575275398 0.9274645903257651 0.9457646089452284 0.9437295951012247 0.9430111483612709 0.9438044188376753 0.94291676088044 0.9448203897058822 0.9447793146718146 0.9756747903763697 0.9443517200880842 0.7781464071146243 0.7741530958132046 0.9458741859897485 0.9443595336278842 0.9431402681807199 1.0 0.9851652042464806 0.9449982148920688 0.945587610383309 0.9759958419167041 0.9459967408661989 0.9476474364406781 0.9463880594530322 0.9462808874045802 0.9455246459537573 0.9462498450905625 0.9445512077056063 0.9435635680280631 0.9451059460128777 0.9443450358290092 0.9454319797330696 0.9443334486141772 0.9443630914439813

GCA_015894005 0.907880966245813 0.9832511670963205 0.9450752765647744 0.822765611764706 0.9421478783419182 0.8647596066252586 0.9434275831873906 0.9285219597249509 0.942722697156984 0.9427734147545028 0.8345939910313902 0.825945281030445 0.8646533841778696 0.8925454554973824 0.9982444574652778 0.8203527359693876 0.9827754023255812 0.8202077910447761 0.9433617884990253 0.9243004200404858 0.8251230580682812 0.9450743182920911 0.9065530075376884 0.8957815431783342 0.9260418793619143 0.9436749191572757 0.9435381758080312 0.9284211050947576 0.9447845662440963 0.9428140115259335 0.9423309297052153 0.9427527373179307 0.9422677466565733 0.9446745961207955 0.9442295934959348 0.9757306101854024 0.9438572120612951 0.7755007429245283 0.7726057888446216 0.9445738379846875 0.9441431987273617 0.9438391486220472 0.9855284797064893 1.0 0.9449652808710716 0.945678812392427 0.9771940782508631 0.9441963407479834 0.9446604788941001 0.9448865927910375 0.9448307267865823 0.9444601272949819 0.944840019436346 0.9445930346534653 0.9439850203562341 0.9438088847305389 0.9438819224159097 0.9435542222779729 0.9444674871794871 0.9440475915974598

GCA_016076075 0.905661424250063 0.9439363919716648 0.9976005079301347 0.821612111111111 0.9849440360360361 0.8645538113795792 0.98516087909498 0.9247284741275571 0.9828799637599093 0.9825540635488923 0.8327318382352943 0.8257551729065448 0.8630468804515137 0.8989808701072051 0.9434814799310516 0.8196128420378084 0.9446850773993808 0.8232751186639321 0.9865827868480724 0.9223952292658482 0.8326537192118226 0.9976006164658634 0.9040655187637971 0.9055057565872021 0.9213510465116279 0.9867407216494846 0.986635222944208 0.9244981065952788 0.9870113429148887 0.9838829135122735 0.9849263197264374 0.9839603864734301 0.9847972888603806 0.9866806961489633 0.9870663021057154 0.9439319198623403 0.9853877137793532 0.7765720349514564 0.7765710058252429 0.9852191378184195 0.98748731696328 0.9865238794604004 0.943791928057554 0.9438242354099975 1.0 0.9876585332164 0.9435007903377111 0.9867737463719581 0.9967959124087591 0.9968994805194805 0.997498452928243 0.9859192370632464 0.9970386711224691 0.9873822839919626 0.9859042937608318 0.9881246961690885 0.9848340877546451 0.9860844418356457 0.9869582422857144 0.9872631826360774

GCA_017151445 0.9056657833804992 0.9436574975466142 0.9888091728828434 0.822457878698225 0.9866440454124189 0.8648655534908416 0.9868297560397917 0.9252965670910872 0.9869800905712959 0.986997924878275 0.8342548476702509 0.8263446742623429 0.8629112908283252 0.9006877464065708 0.9440563898645258 0.8202886587327115 0.9437090692479524 0.8209425441803778 0.9879979665738161 0.9228368411644536 0.8253232426104771 0.9888088350153577 0.9054826732174351 0.9074789902112315 0.9241218163317221 0.989427975348094 0.9893384611872147 0.9251384293451501 0.9884409417453374 0.9869638580614433 0.986741071513992 0.9870835081417625 0.986701077586207 0.9957431511959624 0.9883538698630138 0.9444098262839878 0.9883811027568924 0.7782647576580068 0.7743514364640884 0.9919644264117384 0.9886545703839122 0.9864025149700599 0.9452347066074953 0.9455376207332489 0.9887213946117274 1.0 0.9449803146341463 0.9890364317976513 0.9883540930652812 0.9887652235502435 0.9890713927143492 0.9880866097785977 0.9888899800266312 0.9884610187300138 0.9876633401920439 0.987845819235226 0.9871650256052142 0.9878671121281465 0.9880202667594525 0.9886303263350068

GCA_017151595 0.907121651925078 0.9745329406220548 0.945466085913127 0.8219660370038796 0.943791325446644 0.8635212614445575 0.9445665439163972 0.9287935223367698 0.9435092559379544 0.9430565206812653 0.834455735075735 0.8260356112576958 0.8645464335846115 0.8926576846409404 0.9753938790697675 0.8199345488839986 0.9740737201607186 0.8204112589285713 0.9439315720953083 0.924025321264661 0.8251530424597364 0.9454797744721689 0.9052030469332009 0.8967473507085019 0.924623409147713 0.9455331704934051 0.9454719139574675 0.9282873501345732 0.9472293024344569 0.9432126971883554 0.9439430356709538 0.9429931708542714 0.943752152422693 0.9439333382752658 0.9441104548719177 0.9911606222931388 0.9429458867832848 0.783011859047619 0.7790945168800931 0.9454085471743876 0.944565189468504 0.9432125841053144 0.975871330755232 0.9765560207205085 0.9444719321462947 0.9456136924560135 1.0 0.9462092128069332 0.9462248677373641 0.9452526820475847 0.9451026806358382 0.9446554216572967 0.94494593712503 0.9461221757118521 0.9421843177290837 0.9439969721808247 0.9434527643467644 0.944203046018992 0.9446380564417178 0.9445107788697789

GCA_019659905 0.9061123910239877 0.943884038222884 0.9885501716350497 0.8219376329787234 0.9860250563088944 0.864654295212766 0.9878546907957815 0.924413598827266 0.9853237943668423 0.9854985809109637 0.8334624240601504 0.8247642327887982 0.8633291033394688 0.9006874749034751 0.9435616202658642 0.8190896919127085 0.9439947961990498 0.8206719424460432 0.9874791695743197 0.9232863110770018 0.8243373141345872 0.9885510817524842 0.9052541355846775 0.9072689464285715 0.9242000125376129 0.9988601422107306 0.9987233239982767 0.9257424659906011 0.9896578023770682 0.9874012954599476 0.9854822679227943 0.9874899475441106 0.9853925385146011 0.9892870025304809 0.9886638964015585 0.9440214299975044 0.9881898160073598 0.7791173190661479 0.7755423685251088 0.9890386821705426 0.9887653640381484 0.9851898714189038 0.9462480842157104 0.9445961534603812 0.9882056551724138 0.9891767050847458 0.9455855539323106 1.0 0.9878509473442171 0.9877226744446937 0.9885383999999999 0.9871199358239744 0.9885208182640145 0.9890796758620689 0.9881145248554914 0.9883742062572423 0.9883605694444444 0.9878989017472205 0.9871096403712298 0.9887024668681701

GCA_019660135 0.9063901267459907 0.9447558971871969 0.9994218962360121 0.8221430702280913 0.9853417408529481 0.865836960731576 0.9866756954436451 0.9259869975247524 0.9856566525622943 0.9853508813877169 0.8340388490737928 0.8273175344070278 0.863951938559322 0.9008854698420917 0.9446769150823827 0.8204306373193166 0.945965547067521 0.8216443583460951 0.9876991573295986 0.9234026811033772 0.8264395312042191 0.9994250701932859 0.9051649835067243 0.9068264955935719 0.9237908917197453 0.9873112850196804 0.9872363015284854 0.9254061549962621 0.9886977006556635 0.9865678231619414 0.9858112047904191 0.9866314647072996 0.9858026102588684 0.987884134331745 0.9887065597999092 0.9450089655172415 0.9869993471810089 0.7762488105553367 0.772749224 0.9889168531468532 0.9879153655714947 0.9858067985109353 0.9475505519713262 0.9451760267069791 0.998343694014232 0.9889530890999548 0.9465850918635171 0.9883618271661688 1.0 0.9991794555058919 0.9995110843373495 0.9856794007319305 0.9996159854750858 0.9880243946703423 0.9881995417236662 0.9879791436963399 0.9874629236985976 0.9879567665078285 0.9873289173320806 0.9878399236817762

GCA_019660345 0.9061296039856923 0.9448117777777776 0.9989893877960659 0.8212379338601111 0.9851774937056536 0.8654781463928383 0.9866960198300284 0.9252420247632922 0.9848503157172271 0.9847602096627165 0.8337961863391253 0.8256255912743973 0.8634220558309419 0.9000477907567295 0.9439720129546586 0.8196004623343034 0.9453220106460198 0.8218881023002422 0.9872303668042183 0.9232184416898558 0.8248899389534883 0.998989787276741 0.904906972959439 0.9063125101317122 0.9237599472361809 0.9873768065693432 0.9873545871350365 0.925113075221239 0.988479547022304 0.9857481921438712 0.985390018957346 0.9858920773979108 0.9853251279620853 0.9878363078291815 0.9890122989529964 0.9439992857142858 0.9869632906846242 0.777593745583039 0.773671246504195 0.9889078375387339 0.9876478019487877 0.985373409090909 0.9455716178960097 0.944552518666003 0.9980794048357098 0.9887656839309429 0.9449976035861402 0.9874539699955217 0.998808976602238 1.0 0.9991010597609562 0.9864399796793859 0.9992171174945143 0.9880170947320823 0.9882174659522215 0.9878851319583055 0.9871311839666358 0.9877558398047481 0.987237915218902 0.9875650079311128

GCA_019660505 0.905975520488674 0.9446664033412888 0.9996066947115385 0.8211646160635482 0.9855033960100893 0.8652253695364239 0.9867920584045584 0.9254389598051158 0.9860021136890952 0.9858506312138728 0.8331557599283369 0.8265450115141048 0.8635416325995807 0.9006138424396442 0.9443234115884117 0.8194428738241972 0.9457238089450657 0.821298109332528 0.9877677609195403 0.9231759294057897 0.8249619181659895 0.999607440929115 0.9046526273726273 0.9063317615658363 0.9232717426340973 0.9874353991788319 0.9873865457449236 0.9250404126750185 0.9886999053773368 0.9868127190476189 0.9863211050250058 0.9869455632458234 0.9863056404761903 0.9881565630590341 0.9890875497874245 0.9445908002991773 0.9870128320971004 0.7766198315047023 0.7732334049323787 0.9888856431904022 0.9880697653223969 0.9863994989657551 0.9455423328488373 0.9447627157423526 0.9990557385539672 0.9892832591273376 0.9450493704423919 0.9884539805605786 0.99948505038291 0.9994715960846985 1.0 0.9863505851784906 0.9999459920556107 0.9882339420683414 0.9886106967857946 0.988073224871852 0.9878681924607822 0.988416697986577 0.9874880459503365 0.988037866575592

GCA_019660585 0.9049744087371329 0.9408002877007912 0.9838952149574565 0.8214569893402477 0.9868794295154186 0.8625148845561827 0.9868991100067767 0.9226337066283801 0.9857832914681681 0.9858203875297684 0.8324633610451307 0.8249808357515961 0.8623121997902466 0.8982847771516393 0.9419990308370045 0.8179340360303414 0.9430613088129927 0.8186053621881574 0.9879724383802817 0.9208926497869139 0.829984835042972 0.983891829787234 0.9040797352941177 0.9049528293681249 0.9224110550795592 0.9841386392840751 0.9841032988980716 0.9231739338324078 0.9866856296296297 0.9880142827728209 0.989180033519553 0.9881231965145609 0.9892344407158836 0.9860625869859918 0.9866666870194483 0.9414852165354332 0.9848388138238573 0.7753156560908735 0.7712783645791782 0.9859536675097723 0.98630774282434 0.9896014917477782 0.9439948572120038 0.9426400049419325 0.9852364791248046 0.9866838276415311 0.943518064672988 0.9855895160190866 0.9839263541430193 0.9849226995515694 0.9846598299015219 1.0 0.9849705801664044 0.9855818354285716 0.9841737779850747 0.9844238130495141 0.9885887755555557 0.9826394478951 0.9897565161431066 0.9861973732507455

GCA_019660945 0.9060122239224687 0.944833547386011 0.9997118112602685 0.8214120354505171 0.9854501996328593 0.8652376965845909 0.986590407679545 0.9253608609756097 0.9844683821832448 0.9843976553152325 0.8332444866806346 0.8265304332755633 0.8633421624445026 0.9004041215357234 0.9441680009982532 0.8195205483452304 0.9454637333333336 0.821480849969752 0.9876775201288246 0.922986494296578 0.8252186922406277 0.9997124003205771 0.9045069109947644 0.9061634945278696 0.9225471169811321 0.9872812206251425 0.9872615123175184 0.9249342049643647 0.9884155061102144 0.9848946150212565 0.9847831437337738 0.985043518957346 0.9848483856502241 0.9880386260053621 0.9891148396372486 0.9447202536051716 0.9867326787715759 0.7763797786407767 0.7730284154513205 0.9878183107958325 0.9879975153863688 0.9862072372648002 0.9457053060730705 0.9446526417910448 0.9990014420901049 0.9889708477970627 0.9448798790517658 0.9881696341463415 0.9995669056298097 0.9995838284686126 0.9999280325590629 0.9866241400407886 1.0 0.9879725860507247 0.9888373975044563 0.9883807266666667 0.9874308122534231 0.9884902121546962 0.9871452226345084 0.9879374544211487

GCA_019731005 0.9058358685567012 0.9445011094452775 0.9886123055872291 0.8221846768958395 0.9877820122525919 0.8633635763338614 0.9873577069297401 0.9252422347740668 0.9871991342092915 0.9872682341831918 0.8332147874251499 0.8233792696797367 0.8626527010582011 0.9010330801033593 0.9436518066801619 0.8186075369774919 0.9444653665318503 0.8190754002463055 0.9884460775558167 0.9227216550477667 0.8250109200825715 0.9886715944343065 0.9055491364205257 0.907333124840439 0.922562621559043 0.9891091356321838 0.9889340574712644 0.925137365710081 0.9892106192821958 0.9879329841040463 0.9862592751196172 0.988046928088803 0.9862278075634275 0.9887867679109669 0.9878302249254075 0.9448221764556319 0.9880225689135973 0.7718015513794481 0.77171094 0.9889003059096176 0.9889696756882876 0.9879973198779628 0.9447687861271676 0.9442712439582802 0.9888747471264369 0.9890231114693688 0.9461934997540581 0.9893743918762983 0.9879450508788158 0.9884031809913598 0.9885311597174756 0.9880669443798 0.9885143146630236 1.0 0.987446812776871 0.9881265337995337 0.9880964776754074 0.988477788956481 0.9875222315397159 0.9888308760484624

GCA_021417245 0.9064558589037501 0.9442683951533137 0.9890773996431758 0.8223013237028302 0.9870121634388287 0.8640467156993848 0.9866012411347517 0.9245920320591863 0.9844980105795769 0.984832794117647 0.8356640696635903 0.8262961138979048 0.8636651154855643 0.901779888296522 0.9434626137239783 0.8189591366223908 0.9450237875751505 0.8218625523520485 0.9873124941231781 0.9230255257039524 0.8260029691553764 0.9890781686370733 0.9054430304568528 0.9089308415466263 0.9226102504339203 0.9885607647874308 0.9885327293737 0.9254068552990817 0.988550370027752 0.985248206928839 0.9849045340167755 0.9852175011742602 0.9848730109531577 0.9883092022346369 0.9863946287728576 0.9444213945752304 0.986559903403864 0.7729370247933884 0.7729414994096813 0.9866872831838049 0.9886396889726673 0.9869336691658857 0.9442528673287498 0.9442368001039771 0.988186620566585 0.9875803831330764 0.9427597975506125 0.9881429449541286 0.9880420998864926 0.989244853202847 0.9888878929765886 0.9857482523364486 0.9891826383355468 0.9875047745664739 1.0 0.99073124725033 0.9862241643258428 0.9974200744680853 0.9875235958254269 0.988534517647059

GCA_021417265 0.9065587461139897 0.944550651425762 0.9883415706115265 0.8222688312445351 0.9862296604574392 0.8645266833905467 0.9859089744801512 0.9246291963627427 0.9842505716269383 0.9844525196850394 0.8340237322023629 0.8252696835075493 0.8637793967334035 0.9005815286947688 0.9433488382165605 0.8197886455331412 0.9453557923362176 0.8201883677184466 0.987593415210203 0.922541546312835 0.8269515173203551 0.9883443171321752 0.9053742597730139 0.907691364325417 0.9220108848207477 0.988367417938049 0.9882481377079482 0.924261635375494 0.9882740103920642 0.9863162044817927 0.9865541200094495 0.9864389245548267 0.9864887813535258 0.9879013637400229 0.9867786321709787 0.9432691902024495 0.9875206264447526 0.7743311465721041 0.7741935156559652 0.987176453769887 0.9880452796630002 0.9865841263503993 0.9444355550024888 0.943039453663518 0.9893371595243438 0.9871581641072658 0.9429799528886684 0.9876086550777676 0.9876834278640558 0.9879791033568905 0.9883270972745402 0.9861704022320392 0.9883174177159266 0.9878143074441117 0.990227613611416 1.0 0.986635561572875 0.9892429560997131 0.9870794900849857 0.9879900958167797

GCA_022343635 0.906086891017344 0.9430581655480984 0.9883263653439776 0.8216658529411766 0.989742618724559 0.8638388300395258 0.9899776317638791 0.9244564547016942 0.9874417520035619 0.9872397486095662 0.8333430801561091 0.823353736037625 0.8626663476874002 0.8998909629821381 0.943679213229767 0.8181454338908174 0.9434164558208221 0.8183067767584098 0.9904386792040236 0.9224560187294357 0.8241839876760564 0.9883266377083822 0.9050808572153183 0.9059251071428571 0.9211713421453991 0.9883365809768638 0.9881306052816079 0.9249728155818541 0.9892478996282528 0.9873921237113402 0.9866794000452795 0.9877814400736141 0.9866427723669309 0.9874127700307256 0.9881670880876311 0.94395510910459 0.9893189349519012 0.7708447841871723 0.7708097254743642 0.9865472019635345 0.988933215108245 0.9895572614761475 0.9441568250377074 0.9437594666329626 0.9871884787159806 0.9875866971830984 0.9440712716049383 0.9876538802506383 0.9878064229390681 0.9874290013966482 0.9883701483749413 0.9903827237966712 0.9878898134328359 0.9878079547062987 0.9852223312883438 0.9870877111586961 1.0 0.9849048986722572 0.9908262067415732 0.9889317674097909

GCA_022430505 0.9067696635367765 0.9449899554786051 0.9878626783754116 0.8219745828437133 0.9849247563805105 0.8640606492815327 0.9858109721566776 0.9249180973560661 0.9839032478828108 0.9839297635288767 0.8352537114636337 0.8289042848986584 0.8645173189360021 0.9003205351170567 0.942998374936322 0.8206346153846154 0.9440608621124276 0.8234886059701494 0.9877018814003334 0.9232084047496127 0.8263705534736235 0.9878628935236005 0.9051264866229177 0.9078939969643309 0.9239111387631976 0.9878538404794835 0.9878370290188854 0.9250281932462412 0.9882037399716848 0.9849835322805387 0.984946316886727 0.9851748487712664 0.9849416121549526 0.98673876309795 0.9874074430939227 0.9435968145563309 0.9864875638371291 0.7809780137772675 0.7776535903426792 0.9859119623838659 0.9880324263839811 0.9855964906103286 0.9447004784688997 0.9432510671123682 0.9879401245551601 0.9870891586867306 0.9440767270501835 0.9877478703703703 0.9872665782914117 0.9872287535722137 0.9879922140957448 0.984235592712177 0.9879189609820255 0.9877355573580535 0.9970310693579557 0.98867094 0.9863648389355743 1.0 0.9866852422802851 0.9877885436210133

GCA_022585175 0.9058144248466258 0.942458240332844 0.9881842119944212 0.8214022716627635 0.991520143837132 0.8644492450851899 0.9895675957049487 0.9240441714975846 0.9912735983917802 0.9912528079588643 0.8331870334220643 0.8229163470588234 0.8631502235826457 0.8999648390473698 0.9431981153270094 0.8179271200750471 0.9441669637537239 0.8187783912522469 0.9897315582685905 0.9228339432336543 0.8242620941176472 0.9881844361776331 0.905536773138833 0.9068545678053831 0.922888256227758 0.9872133564013841 0.9871445175438597 0.9248375905395417 0.9889872033503956 0.9887696408012895 0.9902264405225764 0.9887802607291185 0.9901351021344961 0.9881422715587753 0.9887804814898933 0.9429158980155741 0.9874972673357665 0.7745217245508981 0.774481105347167 0.9886946740409508 0.9883649840036562 0.9913830856892396 0.9443216019900497 0.9442684469413234 0.9883546680741262 0.9885720941066853 0.9444485200793258 0.98717148714385 0.9878183293950851 0.9882312898719442 0.9882989999999999 0.9915009357878589 0.9883015827505829 0.9877969874476986 0.9875791730219257 0.9880197577197148 0.9906760478640126 0.9873534080610541 1.0 0.9882168702986097

GCA_022646275 0.9071331395646607 0.9446088609467457 0.9892173079553226 0.8214150386904762 0.9891683468395462 0.8661490490066226 0.9880424019024969 0.9250527638554218 0.988528794227188 0.9884500605214152 0.8343886320474779 0.8243576468851491 0.8641618738501972 0.9021108672431334 0.9449727236281632 0.8203127310521267 0.9441623117206984 0.819427360943168 0.9891686331907228 0.9238088433308022 0.824545142773035 0.9892177547298839 0.9063561860111508 0.9078526999999998 0.9242803279938977 0.9895464020427112 0.9895207733395264 0.9258597876494997 0.9911399862825789 0.9875849646226417 0.9867498096597668 0.9875330335474661 0.9867662225929457 0.9891293362425355 0.9891501356633708 0.9450359446540882 0.9906088731762064 0.7735292366412213 0.7734575462590507 0.9895112203545936 0.999962661604761 0.9878202345366751 0.9448819003505259 0.9447053865461846 0.9891407865952989 0.9894884202066591 0.9452477360834991 0.989183242740999 0.9886975412119806 0.9890453038925563 0.9893267367939629 0.9885173926096996 0.9893347632120797 0.9887723510317645 0.9890595857988166 0.9891984457547168 0.9895437255803263 0.9886357051736355 0.9888465802157448 1.0
