# Supplementary material for: Coexistence of blaIMP-4, blaNDM-1 and blaOXA-1 in blaKPC-2-producing Citrobacter freundii of clinical origin in China
Source: Front Microbiol. 2023 Jun 12;14:1074612. doi: 10.3389/fmicb.2023.1074612 (PMC10291173; doi:10.3389/fmicb.2023.1074612)
Supplement: Supplementary file 1 [file Data_Sheet_1.zip › Table S1.docx]

| Table S1. The pertinent primer sequences | | | |
| --- | --- | --- | --- |
| name | sequences | Destination fragment length | Annealing temperature |
| blaNDM- F  blaNDM- R | ATGGAATTGCCCAATATTATGCAC  TCAGCGCAGCTTGTCGGC | 813 bp | 55 |
| blaKPC-F  blaKPC-R | ATGTCACTGTATCGCCGTC  TTACTGCCCGTTGACGCC | 902 bp | 55 |
| blaVIM-F  blaVIM-R | TTTGGTCGCATATCGCAACG  CCATTCAGCCAGATCGGCAT | 500 bp | 55 |
| blaIMP-F  blaIMP-R | GTTTATGTTCATACWTCG  GGTTTAAYAAAACAACCAC | 432 bp | 42 |
| blaOXA-48-F  blaOXA-48-R | TTGGTGGCATCGATTATCGG  GAGCACTTCTTTTGTGATGGC | 700 bp | 55 |
| blaOXA-23-F  blaOXA-23-R | GAT CGG ATT GGA GAA CCA GA  ATT TCT GAC CGC ATT TCC AT | 501bp | 55 |
| MCR-1-F MCR-1-R | AGTCCGTTTGTTCTTGTGGC  AGATCCTTGGTCTCGGCTTG | 320 bp | 58 |
| MCR-2-F  MCR-2-R | GCGATGGCGGTCTATCCTGTAT  TGCGATGACATGGGGTGTCAGC | 378bp | 55 |
| MCR-3-F  MCR-3-R | TATGGGTTACTATTGCTGG  CTACCCTGATGCTCATCG | 814bp | 55 |
| MCR-4-F  MCR-4-R | GTCATAGTGGTATAAAAGTACAG  CCACCGTCTATCAGAGCCAAC | 669bp | 55 |
| MCR-5-F  MCR-5-R | GCGGTTGTCTGCATTTATCAC  CTTTGAAAACCTGTCTTCGGCA | 1042bp | 55 |
| MCR-6-F  MCR-6-R | GTCCGGTCAATCCCTATCTGT  ATCACGGGATTGACATAGCTAC | 556bp | 55 |
| MCR-7-F  MCR-7-R | TGCTCAAGCCCTTCTTTTCGT  TTCATCTGCGCCACCTCGT | 892bp | 55 |
| MCR-8-F  MCR-8-R | TCAACAATTCTACAAAGCGTG  AATGCTGCGCGAATGAAG | 856bp | 55 |
| MCR-9-F  MCR-9-R | ATG CCT GTA CTT TTC AGG GTG AAA G  TTC CGC GAA TGC CGT GGC TAA | 1619bp | 55 |
| MCR-10-F  MCR-10-R | AAAAAAGAGCTCTCCGCTTTGTATCCCAATAC  AAAAAAGAATTCTTTTATAATTTCCGGCAGCA |  | 55 |
|  |  |  |  |
