# Supplementary material for: Coexistence of blaIMP-4, blaNDM-1 and blaOXA-1 in blaKPC-2-producing Citrobacter freundii of clinical origin in China
Source: Front Microbiol. 2023 Jun 12;14:1074612. doi: 10.3389/fmicb.2023.1074612 (PMC10291173; doi:10.3389/fmicb.2023.1074612)
Supplement: Supplementary file 1 [file Data_Sheet_1.zip › Table S2.docx]

# pertinent material information

MUELLER-HINTON AGAR CM0337 (OXOID, Hampshire, United Kingdom).

PETRI DISHES (Hang zhou KE Di chen Biological technology Co., Ltd, Hangzhou, China).

Antibiotics (meilunbio, Dalian, China)

Antimicrobial susceptibility methodology

Agar dilution methods

1. Prepare antibiotic solution

The 31st CLSI standard has set the concentration gradients for each antimicrobial and determined the necessary dilution ratio for preparing MHA culture medium containing antimicrobial by diluting and adding them to the corresponding concentration of empty plates.

1. Preparation of MHA medium

After measuring out 15.20 g of MHA powder, it should be combined with 400mL of pure water. The mixture should be thoroughly mixed and then sealed with tin foil. To sterilize the mixture, it should be subjected to high pressure at 121 ℃ for 30 minutes. Once sterilized, the mixture should be cooled to around 65 ℃ for backup purposes. After adding antibiotics to an empty plate, pour 20ml of agar into it and mix thoroughly. Allow the mixture to sit at room temperature for 24 hours.

1. Medium that contain antibiotics are prepared through a specific process.

To test the drug sensitivity of a bacterial strain, inoculate the strain onto an MHA plate the night before and let it grow overnight. Prepare sterile glass tubes and add 3ml of sterile physiological saline to each. Use a sterile inoculating loop to pick individual bacterial colonies and grind them on the tube wall. Adjust the turbidity of the bacterial suspension to 0.5 McFarlandstandard turbidity using a turbidimeter, dilute with high-pressure sterilized physiological saline 1:9. Sterilize the needle tube with an alcohol lamp and use a multi-point inoculum to sequentially inoculate the bacterial suspension onto drug-containing MHA plates from low to high drug concentration. Leave it at room temperature in the dark for 1-2 hours until the bacterial solution is fully absorbed. Incubate at 37 ℃ for 16-20 hours before reading the results. Use a multi-point inoculum to inoculate the samples onto non-medicated MHA medium before and after each batch of samples as a pre and post control.

1. Assess the outcomes.

Upon examining the non-medicated MHA plates before and after the experiment, it was evident that the sample strains thrived, indicating that the outcomes were trustworthy. If not, the outcome cannot be trusted.

After placing the culture medium on a black background desktop, read the MIC values in order and determine the appropriate classification based on the CLSI standard. The concentration of the antibiotic plate at which the sample strain does not grow is considered the MIC value for the antibiotic in the sample.

Broth microdilution methods

1. Prepare antibiotic solution

In order to have it available for later, create antimicrobial solution at a concentration of 64ug/ml.

1. To prepare a broth culture medium that includes antibiotics, follow these steps.

Firstly, sterilize a 96 well plate and label it with a serial number. Then, add 180μL of MH broth culture medium to the first well in each row, followed by adding 100μL of the same medium to every well in that row. Next, add 20μL of the antimicrobial from step (1) to the first well and mix it well. After mixing thoroughly, take in 100μL and transfer it to the next hole. Repeat this process until the 11th hole is reached, taking in 100 μ each time. Remove 100ul of liquid from the 11th hole. Finally, use the 12th well as a positive control and avoid exposing it to light during dilution.

1. The process of preparing and inoculating bacterial solution.

Adjust the turbidity of the bacterial suspension to 0.5 McFarlandstandard turbidity using a turbidimeter, dilute with high-pressure sterilized physiological saline 1:8. Following this, add 4μL of bacterial suspension into each hole of the 96 well plate and then cover it with a sealed lid. The plate should then be incubated at a temperature of 37 ℃ for 16-20 hours.

1. Assess the outcomes.

After the cultivation period is over, the 96 well plate should be retrieved and the positive control well in the 12th position should be examined. If bacteria are present and growing in the well, the sample can be considered meaningful. If not, it should be discarded. Additionally, the results should be evaluated in conjunction with the quality control bacteria. If the quality control bacteria results are not satisfactory, then the entire batch of results should be deemed invalid.

The EUCAST guidelines should be referred to for interpreting the results after reading the MIC value. The MIC value of the test strain for the drug is the maximum concentration of the drug that does not result in growth in the well.
