# Supplementary material for: Coexistence of blaIMP-4, blaNDM-1 and blaOXA-1 in blaKPC-2-producing Citrobacter freundii of clinical origin in China
Source: Front Microbiol. 2023 Jun 12;14:1074612. doi: 10.3389/fmicb.2023.1074612 (PMC10291173; doi:10.3389/fmicb.2023.1074612)
Supplement: Supplementary file 1 [file Data_Sheet_1.zip › Table S3.docx]

Table S3. The quality of the assembly has been included in the supplementary material.

Starting Unicycler (2022-07-14 18:31:12)

----------------------------------------

Welcome to Unicycler, an assembly pipeline for bacterial genomes. Since you provided both short and long reads, Unicycler will perform a hybrid assembly. It will first use SPAdes to make a short-read assembly graph, and then it will use various methods to scaffold that graph with the long reads.

For more information, please see https://github.com/rrwick/Unicycler

Command: ./unicycler-runner.py --spades_path /home/xhao/software/SPAdes/bin/spades.py --racon_path /home/xhao/miniconda2/bin/racon --bowtie2_path /home/xhao/software/bowtie2-2.4.2-linux-x86_64/bowtie2 --bowtie2_build_path /home/xhao/software/bowtie2-2.4.2-linux-x86_64/bowtie2-build --pilon_path /home/xhao/software/pilon-1.22.jar -1 /data5/xuhao/3data/wang1_wang9/illumina_Result/01.Cleandata/wang1/wang1.L350_FDSW220003794-2r_1.fq.clean.gz -2 /data5/xuhao/3data/wang1_wang9/illumina_Result/01.Cleandata/wang1/wang1.L350_FDSW220003794-2r_2.fq.clean.gz -l /data5/xuhao/3data/wang1_wang9/01.Cleandata/wang1/barcode07.fastq -o /data5/xuhao/3data/wang1_wang9/01.Cleandata/wang1/wang1_hy

Unicycler version: v0.4.9

Using 8 threads

Making output directory:

/data5/xuhao/3data/wang1_wang9/01.Cleandata/wang1/wang1_hy

Dependencies:

Program Version Status

spades.py 3.13.0 good

racon 1.4.13 good

makeblastdb 2.9.0+ good

tblastn 2.9.0+ good

bowtie2-build 2.4.2 good

bowtie2 2.4.2 good

samtools 1.9 good

java 1.8.0_282 good

pilon 1.22 good

bcftools not used

SPAdes read error correction (2022-07-14 18:32:39)

--------------------------------------------------

Unicycler uses the SPAdes read error correction module to reduce the number of errors in the short read before SPAdes assembly. This can make the assembly faster and simplify the assembly graph structure.

Command: /home/xhao/software/SPAdes/bin/spades.py -1 /data5/xuhao/3data/wang1_wang9/illumina_Result/01.Cleandata/wang1/wang1.L350_FDSW220003794-2r_1.fq.clean.gz -2 /data5/xuhao/3data/wang1_wang9/illumina_Result/01.Cleandata/wang1/wang1.L350_FDSW220003794-2r_2.fq.clean.gz -o /data5/xuhao/3data/wang1_wang9/01.Cleandata/wang1/wang1_hy/spades_assembly/read_correction --threads 8 --only-error-correction --phred-offset 33

Corrected reads:

/data5/xuhao/3data/wang1_wang9/01.Cleandata/wang1/wang1_hy/spades_assembly/corrected_1.fastq.gz

/data5/xuhao/3data/wang1_wang9/01.Cleandata/wang1/wang1_hy/spades_assembly/corrected_2.fastq.gz

Choosing k-mer range for assembly (2022-07-14 18:55:35)

-------------------------------------------------------

Unicycler chooses a k-mer range for SPAdes based on the length of the input reads. It uses a wide range of many k-mer sizes to maximise the chance of finding an ideal assembly.

SPAdes maximum k-mer: 127

Median read length: 150

K-mer range: 27, 47, 63, 77, 89, 99, 107, 115, 121, 127

SPAdes assemblies (2022-07-14 18:56:59)

---------------------------------------

Unicycler now uses SPAdes to assemble the short reads. It scores the assembly graph for each k-mer using the number of contigs (fewer is better) and the number of dead ends (fewer is better). The score function is 1/(c*(d+2)), where c is the contig count and d is the dead end count.

K-mer Contigs Dead ends Score

27 too complex

47 525 1 6.35e-04

63 430 1 7.75e-04

77 370 1 9.01e-04

89 299 1 1.11e-03

99 272 1 1.23e-03

107 269 1 1.24e-03

115 257 1 1.30e-03 ← best

121 250 2 1.00e-03

127 238 2 1.05e-03

Read depth filter: removed 6 contigs totalling 1992 bp

Deleting /data5/xuhao/3data/wang1_wang9/01.Cleandata/wang1/wang1_hy/spades_assembly/

Determining graph multiplicity (2022-07-14 19:13:47)

----------------------------------------------------

Multiplicity is the number of times a sequence occurs in the underlying sequence. Single-copy contigs (those with a multiplicity of one, occurring only once in the underlying sequence) are particularly useful.

Saving /data5/xuhao/3data/wang1_wang9/01.Cleandata/wang1/wang1_hy/001_best_spades_graph.gfa

Cleaning graph (2022-07-14 19:13:48)

------------------------------------

Unicycler now performs various cleaning procedures on the graph to remove overlaps and simplify the graph structure. The end result is a graph ready for bridging.

Graph overlaps removed

Removed zero-length segments:

155, 157, 159, 160, 162, 164, 165, 169, 172, 174, 178, 180, 183, 188, 192, 202, 205, 206, 207, 208, 210, 211, 222, 236

Removed zero-length segments:

161, 228, 235, 253

Removed zero-length segments:

156, 224

Merged small segments:

219, 220, 225, 226, 227, 229, 230, 231, 232, 233, 237, 238, 239, 240, 241, 242, 243, 244, 245, 246, 247, 248, 249, 250, 251, 252, 254, 255, 256, 257

Saving /data5/xuhao/3data/wang1_wang9/01.Cleandata/wang1/wang1_hy/002_overlaps_removed.gfa

Unicycler now selects a set of anchor contigs from the single-copy contigs. These are the contigs which will be connected via bridges to form the final assembly.

73 anchor segments (5,263,225 bp) out of 200 total segments (5,332,957 bp)

Creating SPAdes contig bridges (2022-07-14 19:13:49)

----------------------------------------------------

SPAdes uses paired-end information to perform repeat resolution (RR) and produce contigs from the assembly graph. SPAdes saves the graph paths corresponding to these contigs in the contigs.paths file. When one of these paths contains two or more anchor contigs, Unicycler can create a bridge from the path.

Bridge

Start Path End quality

-28 -116 47 63.2

-27 -116 48 61.8

-3 -164 → -82 → 174 → 164 41 11.0

1 137 72 29.9

4 -174 -35 63.2

5 -139 → 175 → -139 -24 25.8

6 -143 -26 63.0

8 157 33 62.5

12 49 -33 3.6

15 -157 -17 62.7

36 -150 -73 50.0

38 147 → -76 → 122 → 81 → -155 → 96 → -130 → -119 → -154 → -136 → -134 → -129 → -140 → -132 → -158 → -95 → -148 -69 4.3

40 -111 25 45.1

41 144 → 99 → -137 -72 5.5

51 -142 61 63.1

53 -142 55 55.2

56 111 62 36.6

57 138 → 87 → 138 56 5.9

61 147 -71 35.5

68 -143 60 46.7

70 148 → 94 → 158 → 131 → 140 → -128 → 134 → -135 → 154 → 118 → 130 → 97 → 155 → -80 → -122 71 7.1

74 150 29 61.2

Creating loop unrolling bridges (2022-07-14 19:13:49)

-----------------------------------------------------

When a SPAdes contig path connects an anchor contig with the middle contig of a simple loop, Unicycler concludes that the sequences are contiguous (i.e. the loop is not a separate piece of DNA). It then uses the read depth of the middle and repeat contigs to guess the number of times to traverse the loop and makes a bridge.

Loop count Loop count Loop Bridge

Start Repeat Middle End by repeat by middle count quality

-30 -149 185 17 0.21 0.84 1 24.4

5 -139 175 -24 0.69 0.67 1 33.1

57 138 87 56 0.26 0.73 1 19.5

Loading reads (2022-07-14 19:13:49)

-----------------------------------

180,908 / 180,908 (100.0%) - 1,302,906,540 bp

Assembling contigs and long reads with miniasm (2022-07-14 19:14:04)

--------------------------------------------------------------------

Unicycler uses miniasm to construct a string graph assembly using both the short read contigs and the long reads. It will then use the resulting string graph to produce bridges between contigs. This method requires decent coverage of long reads and therefore may not be fruitful if long reads are sparse. However, it does not rely on the short read assembly graph having good connectivity and is able to bridge an assembly graph even when it contains many dead ends.

Unicycler uses two types of "reads" as assembly input: anchor contigs from the short-read assembly and actual long reads which overlap two or more of these contigs. It then assembles them with miniasm.

Aligning long reads to graph using minimap

Saving to /data5/xuhao/3data/wang1_wang9/01.Cleandata/wang1/wang1_hy/miniasm_assembly/01_assembly_reads.fastq:

72 short-read contigs

20,623 long reads

Finding overlaps with minimap...

success

3,432,111 overlaps

Assembling reads with miniasm...

success

338 segments, 336 links

Saving /data5/xuhao/3data/wang1_wang9/01.Cleandata/wang1/wang1_hy/miniasm_assembly/11_branching_paths_removed.gfa

Merging segments into unitigs:

3 circular unitigs

2 linear unitigs

total size = 5,177,983 bp

Saving /data5/xuhao/3data/wang1_wang9/01.Cleandata/wang1/wang1_hy/miniasm_assembly/12_unitig_graph.gfa

Polishing miniasm assembly with Racon (2022-07-14 19:16:01)

-----------------------------------------------------------

Unicycler now uses Racon to polish the miniasm assembly. It does multiple rounds of polishing to get the best consensus. Circular unitigs are rotated between rounds such that all parts (including the ends) are polished well.

Saving to /data5/xuhao/3data/wang1_wang9/01.Cleandata/wang1/wang1_hy/miniasm_assembly/racon_polish/polishing_reads.fastq:

72 short-read contigs

180,908 long reads

Polish Assembly Mapping

round size quality

begin 5,177,983 118,107.56

1 5,202,723 130,509.83

Best polish: /data5/xuhao/3data/wang1_wang9/01.Cleandata/wang1/wang1_hy/miniasm_assembly/racon_polish/006_rotated.fasta

Saving /data5/xuhao/3data/wang1_wang9/01.Cleandata/wang1/wang1_hy/miniasm_assembly/13_racon_polished.gfa

Saving /data5/xuhao/3data/wang1_wang9/01.Cleandata/wang1/wang1_hy/003_long_read_assembly.gfa

Contigs in the short-read assembly graph which end in dead ends may contain bogus sequence near the dead end. Unicycler therefore uses the read clipping values from the miniasm assembly to trim these dead ends to only the parts which aligned well to long reads.

Trimmed Trimmed

Previous from from Final

Segment length start end length

10 124,551 - 80 124,471

32 52,086 - 64 52,022

Unicycler now places the single copy contigs back into the unitig graph. This serves two purposes: a) it replaces long read assembly sequences (which may be error prone) with Illumina assembly sequence (which is probably quite accurate), improving the assembly quality, and b) it defines inter-contig sequences for use in building bridges.

Searching for contigs using 5,000 bp of contig ends.

Contig Result Start pos End pos Strand

1 found in unitig 1 2126512 2777539 -

2 found in unitig 1 1320154 1657504 -

3 found in unitig 1 1777379 2093175 -

4 found in unitig 1 678308 954588 +

5 not found

6 found in unitig 1 3167041 3433422 -

7 found in unitig 1 4077167 4288933 -

8 found in unitig 1 4576222 4740197 +

9 found in unitig 1 200811 359200 -

10 found in unitig 1 3488116 3612563 -

11 found in unitig 1 560739 674322 +

12 found in unitig 1 4806523 4913107 -

13 found in unitig 1 2888799 2993996 +

14 found in unitig 1 2782427 2885305 +

15 found in unitig 1 4441796 4543545 -

16 found in unitig 1 3618079 3719672 +

17 found in unitig 1 4346689 4441675 +

18 found in unitig 1 1678532 1770681 +

19 found in unitig 1 361115 451046 -

20 found in unitig 1 3986393 4071644 +

21 found in unitig 1 1230503 1315583 +

22 found in unitig 1 3011549 3090251 +

23 found in unitig 1 478728 557244 +

24 not found

25 found in unitig 2 -7079 66850 -

26 found in unitig 1 3095729 3166873 +

27 found in unitig 1 1158887 1229486 +

28 found in unitig 1 1073889 1140315 +

29 found in unitig 1 3833156 3894917 -

30 found in unitig 1 4291038 4346218 -

31 found in unitig 1 3730264 3785034 +

32 found in unitig 1 3436047 3488058 +

33 found in unitig 1 4740318 4790834 +

34 found in unitig 1 46407 90065 +

35 found in unitig 1 954648 997399 -

36 found in unitig 1 114765 152932 +

37 found in unitig 1 3948328 3980811 -

38 found in unitig 1 3913379 3945706 -

39 found in unitig 1 3790492 3822378 -

40 found in unitig 2 67283 94428 -

41 found in unitig 1 2095164 2121783 +

42 found in unitig 1 4544471 4570608 +

43 found in unitig 2 95732 120946 -

44 found in unitig 1 455935 478265 +

45 found in unitig 1 90853 112960 +

46 found in unitig 1 4916602 4938420 -

47 not found

48 found in unitig 1 1141843 1158582 -

50 found in unitig 1 1038233 1053446 -

51 found in unitig 1 180424 195310 -

52 found in unitig 2 124661 139354 +

53 not found

54 found in unitig 1 1659300 1672071 -

55 found in unitig 1 4944803 4955252 -

56 found in unitig 3 22327 32044 -

57 found in unitig 3 33698 42968 -

58 found in unitig 3 609 9844 +

59 found in unitig 1 2998889 3008057 +

60 found in unitig 3 56773 64980 +

61 found in unitig 1 172778 180251 -

62 found in unitig 3 15199 21894 -

63 found in unitig 1 3721717 3728139 +

64 found in unitig 3 43788 48729 -

66 found in unitig 1 4939765 4943983 -

67 found in unitig 1 3823301 3827519 -

68 found in unitig 3 52542 56605 +

69 found in unitig 1 3900595 3904596 +

70 found in unitig 1 159398 163276 +

71 found in unitig 1 169048 172632 +

72 found in unitig 1 2122914 2126288 -

73 found in unitig 1 153072 156220 -

74 found in unitig 1 3895057 3898205 -

Searching for contigs using 2,500 bp of contig ends.

Contig Result Start pos End pos Strand

5 not found

24 not found

47 not found

53 not found

Searching for contigs using 1,000 bp of contig ends.

Contig Result Start pos End pos Strand

5 not found

24 not found

47 found in unitig 1 1054973 1073584 -

53 not found

Searching for contigs using 500 bp of contig ends.

Contig Result Start pos End pos Strand

5 not found

24 not found

53 not found

Saving /data5/xuhao/3data/wang1_wang9/01.Cleandata/wang1/wang1_hy/miniasm_assembly/15_contigs_placed.gfa

Creating miniasm/Racon bridges (2022-07-14 19:40:25)

----------------------------------------------------

Now that the miniasm/Racon string graph is complete, Unicycler will use it to build bridges between anchor segments.

Start → end Best path Quality

1/68 -9 → -19 -151, -177, 186, 124, 103, 109, -117 81.012

2/68 -19 → 44 93, -199, -78, -199, 83, 179, 127 51.743

3/68 44 → 23 110 66.709

4/68 23 → 11 -108, 200, 78, 200, 98 73.511

5/68 11 → 4 -127, -98, -200, -78, -200, 108, -161, 72.990

-156

6/68 4 → -35 -174 99.290

7/68 -35 → -50 23.201

8/68 -50 → -47 102, -121, -105 73.373

9/68 -47 → 28 116 96.987

10/68 28 → -48 102, 120, -105 87.506

11/68 34 → 45 -92 85.597

12/68 -48 → 27 116 94.842

13/68 27 → 21 145, -92, 170 90.643

14/68 21 → -2 93, -199, 83, 179, 127, -101, -98, -200, 71.609

108

15/68 -2 → -54 -85, -106 74.169

16/68 -54 → 18 -127, -179, -83, 199, -93, 113, -98, 59.705

-200, -78, -200, 108

17/68 18 → -3 -108, 200, 78, 199, 78, 200, 98, -91, 65.294

156

18/68 -3 → 41 -164, -82, 174, 164 71.493

19/68 41 → -72 144, 99, -137 26.621

20/68 -72 → -1 -137 46.273

21/68 -1 → 14 -127, -179, -83, 199, 78, 199, -93 69.419

22/68 45 → 36 192, -85, -106 84.068

23/68 14 → 13 -108, 200, 78, 200, 98 73.026

24/68 13 → 59 -127, -179, -83, 199, 78, 199, -93 55.667

25/68 59 → 22 -108, 200, 78, 200, 98 66.765

26/68 22 → 26 -152, -173, 75, 169, -126, -195, 89, 60.560

-191, 194, 107, -198, -162, -184, -172,

-178

27/68 26 → -6 143 97.952

28/68 -6 → 32 -127, -179, -83, 199, -93 80.973

29/68 32 → -10 99.351

30/68 -10 → 16 -133, -173, 75, 169, 153, -196, 89, 68.920

-190, 194, 107, -197, -162, -184, -172,

-178, 167

31/68 16 → 63 -166, 117, 187, 144, -125, -103, 123, 27.179

-186, 176, 151, 181

32/68 63 → 31 -181, -151, -176, -123, 103, 109, -117, 28.386

166, -160

33/68 36 → -73 -150 77.976

34/68 31 → -39 182, -173, 75, 171, -126, -195, 89, 66.740

-190, 193, 107, -197, -162, -184, -172,

-178, 167

35/68 -39 → -67 -90 69.695

36/68 -67 → -29 -189, -133, -173, 75, 171, 112, -196, 59.012

89, -191, 194, 107, -197, -162, -184

37/68 -29 → -74 -150 95.449

38/68 -74 → 69 -86, 163, -170, 92, -145 68.283

39/68 69 → -38 148, 95, 158, 132, 140, 129, 134, 136, 44.754

154, 119, 130, -96, 155, -81, -122, 76,

-147

40/68 -38 → -37 -127, -179, -83, 199, -93 78.567

41/68 -37 → 20 182, -173, 75, 168, 112, -195, 89, -188, 68.106

107, -197, -162, -180, -146

42/68 20 → -7 -152, -173, 75, 169, -126, -195, 89, 63.761

-190, 193, 107, -198, -162, -180, -146

43/68 -7 → -30 -151, -177, 186, -123, 103, 109, -117, 77.379

166, -160

44/68 -73 → 70 -86, 104, 85, -192 76.981

45/68 -30 → 17 -149, 185, -149, 185, -149 95.598

46/68 17 → -15 157 97.942

47/68 -15 → 42 90 84.745

48/68 42 → 8 172, 184, 162, 197, -107, 188, -89, 195, 66.652

-112, -168, -75, 173, 141

49/68 8 → 33 157 97.687

50/68 33 → -12 -49 49.034

51/68 -12 → -46 -108, 200, 78, 200, 98 71.122

52/68 -46 → -66 84, 183 43.834

53/68 -66 → -55 165, 100 72.706

54/68 70 → 71 148, 94, 158, 131, 140, -128, 134, -135, 66.833

154, 118, 130, 97, 155, -80, -122

55/68 -25 → -40 111 93.555

56/68 -40 → -43 -84 88.521

57/68 -43 → 52 114, -77, -115, -159, 114 75.399

58/68 52 → -25 -159, 114, 79, -115, -159, 114 77.959

59/68 58 → -62 100, 88, -108, 200, 78, 200, 98 66.997

60/68 -62 → -56 -111 75.874

61/68 -56 → -57 -138, -87, -138 46.504

62/68 -57 → -64 -100, -165 55.475

63/68 -64 → 68 -78, -199, 83 75.249

64/68 68 → 60 -143 72.668

65/68 71 → -61 -147 55.316

66/68 60 → 58 165, 100 92.606

67/68 -61 → -51 142 98.119

68/68 -51 → -9 180, 162, 197, -107, 188, -89, 195, 126, 59.537

-169, -75, 173, 133, 189

Creating simple long read bridges (2022-07-14 19:44:20)

-------------------------------------------------------

Unicycler uses long read alignments (from minimap) to resolve simple repeat structures in the graph. This takes care of some "low-hanging fruit" of the graph simplification.

Aligning long reads to graph using minimap

Two-way junctions are defined as cases where two graph contigs (A and B) join together (C) and then split apart again (D and E). This usually represents a simple 2-copy repeat, and there are two possible options for its resolution: (A→C→D and B→C→E) or (A→C→E and B→C→D). Each read which spans such a junction gets to "vote" for option 1, option 2 or neither. Unicycler creates a bridge at each junction for the most voted for option.

Op. 1 Op. 2 Neither Final Bridge

Junction Option 1 Option 2 votes votes votes op. quality

142 -61 → 142 → -53, -61 → 142 → -51, 0 381 4 2 86.4

-55 → 142 → -51 -55 → 142 → -53

90 -15 → 90 → 39, -15 → 90 → 42, 1 377 2 2 75.3

67 → 90 → 42 67 → 90 → 39

116 -48 → 116 → 27, -48 → 116 → 28, 311 0 7 1 97.7

-47 → 116 → 28 -47 → 116 → 27

143 -60 → 143 → -68, -60 → 143 → -6, 270 0 3 1 98.5

26 → 143 → -6 26 → 143 → -68

111 -25 → 111 → -40, -25 → 111 → 62, 282 0 6 1 77.4

56 → 111 → 62 56 → 111 → -40

110 44 → 110 → 23, 44 → 110 → 35, 323 0 3 1 83.2

50 → 110 → 35 50 → 110 → 23

150 73 → 150 → -36, 73 → 150 → 29, 457 2 3 1 95.7

74 → 150 → 29 74 → 150 → -36

157 8 → 157 → -15, 8 → 157 → 33, 17 0 431 3 2 98.4

17 → 157 → 33 → 157 → -15

Simple loops are parts of the graph where two contigs (A and B) are connected via a repeat (C) which loops back to itself (via D). It is possible to traverse the loop zero times (A→C→B), one time (A→C→D→C→B), two times (A→C→D→C→D→C→B), etc. Long reads which span the loop inform which is the correct number of times through. In this step, such reads are found and each is aligned against alternative loop counts. A reads casts its "vote" for the loop count it agrees best with, and Unicycler creates a bridge using the most voted for count.

Read Loop Bridge

Start Repeat Middle End count Read votes count quality

-30 -149 185 17 202 2 loops: 202 votes 2 100.0

5 -139 175 -24 188 1 loop: 188 votes 1 89.6

12 49 -33 13 0 loops: 13 votes 0 99.8

57 138 87 56 56 0 loops: 1 vote 1 52.7

1 loop: 55 votes

Determining low score threshold (2022-07-14 19:48:39)

-----------------------------------------------------

Before conducting semi-global alignment of the long reads to the assembly graph, Unicycler must determine a minimum alignment score threshold such that nonsense alignments are excluded. To choose a threshold automatically, it examines alignments between random sequences and selects a score a few standard deviations above the mean.

Automatically choosing a threshold using random alignment scores.

Random alignment mean score: 61.66

standard deviation: 1.31

Low score threshold: 61.66 + (7 x 1.31) = 70.86

Aligning reads (2022-07-14 19:49:00)

------------------------------------

180,908 / 180,908 (100.0%)

Read alignment summary (2022-07-15 00:30:00)

--------------------------------------------

Total read count: 180,908

Fully aligned reads: 164,905

Partially aligned reads: 14,574

Unaligned reads: 1,429

Total bases aligned: 1,293,600,136 bp

Mean alignment identity: 90.6%

Deleting /data5/xuhao/3data/wang1_wang9/01.Cleandata/wang1/wang1_hy/read_alignment/

Building long read bridges (2022-07-15 00:30:02)

------------------------------------------------

Unicycler uses the long read alignments to produce bridges between anchor segments. These bridges can be formed using as few as one long read, giving Unicycler the ability to bridge the graph even when long-read depth is low.

Start → end Best path Quality

1/109 59 → 22 -108, 200, 78, 200, 98 70.799

2/109 13 → 59 -127, -179, -83, 199, 78, 199, -93 64.377

3/109 62 → 14 6.559

4/109 -10 → 16 -133, -173, 75, 169, 153, -196, 89, 80.015

-190, 194, 107, -197, -162, -184, -172,

-178, 167

5/109 29 → 67 184, 162, 197, -107, -194, 191, -89, 63.086

196, -112, -171, -75, 173, 133, 189

6/109 9 → 51 -189, -133, -173, 75, 169, -126, -195, 69.520

89, -188, 107, -197, -162, -180

7/109 -20 → 37 146, 180, 162, 197, -107, 188, -89, 195, 79.896

-112, -168, -75, 173, -182

8/109 20 → -7 -152, -173, 75, 169, -126, -195, 89, 76.811

-190, 193, 107, -198, -162, -180, -146

9/109 52 → -25 -159, 114, 79, -115, -159, 114 81.951

10/109 22 → 26 -152, -173, 75, 169, -126, -195, 89, 72.649

-191, 194, 107, -198, -162, -184, -172,

-178

11/109 -43 → 52 114, -77, -115, -159, 114 80.654

12/109 -1 → 14 -127, -179, -83, 199, 78, 199, -93 81.776

13/109 46 → 12 -98, -200, -78, -200, 108 76.561

14/109 23 → 11 -108, 200, 78, 200, 98 79.529

15/109 39 → -31 -167, 178, 172, 184, 162, 197, -107, 78.132

-193, 190, -89, 195, 126, -171, -75,

173, -182

16/109 -69 → 74 145, -92, 170, -163, 86 62.202

17/109 37 → 38 93, -199, 83, 179, 127 80.253

18/109 63 → 31 -181, -151, -176, -123, 103, 109, -117, 24.733

166, -160

19/109 -19 → 44 93, -199, -78, -199, 83, 179, 127 60.657

20/109 42 → 8 172, 184, 162, 197, -107, 188, -89, 195, 79.993

-112, -168, -75, 173, 141

21/109 -6 → 32 -127, -179, -83, 199, -93 82.940

22/109 70 → 71 148, 94, 158, 131, 140, -128, 134, -135, 74.295

154, 118, 130, 97, 155, -80, -122

23/109 14 → 13 -108, 200, 78, 200, 98 78.925

24/109 -70 → 73 192, -85, -104, 86 69.642

25/109 16 → 63 -166, 117, 187, 144, -125, -103, 123, 25.332

-186, 176, 151, 181

26/109 30 → 7 160, -166, 117, -109, -103, 123, -186, 71.092

177, 151

27/109 11 → 4 -127, -98, -200, -78, -200, 108, -161, 81.860

-156

28/109 21 → -2 93, -199, 83, 179, 127, -101, -98, -200, 83.332

108

29/109 19 → 9 117, -109, -103, -124, -186, 177, 151 76.122

30/109 -18 → 54 -108, 200, 78, 200, 98, -113, 93, -199, 74.650

83, 179, 127

31/109 45 → 36 192, -85, -106 81.250

32/109 69 → -38 148, 95, 158, 132, 140, 129, 134, 136, 59.917

154, 119, 130, -96, 155, -81, -122, 76,

-147

33/109 -24 → 34 93, -199, 83, 179, 127 81.458

34/109 18 → -3 -108, 200, 78, 199, 78, 200, 98, -91, 84.465

156

35/109 43 → 40 84 81.967

36/109 38 → 29 147, -76, 122, 81, -155, 96, -130, -119, 59.468

-154, -136, -134, -129, -140, -132,

-158, -95, -148, -69, 145, -92, 170,

-163, 86, 74, 150

37/109 -3 → 41 -164, -82, 174, 164 69.766

38/109 54 → 2 106, 85 71.296

39/109 9 → 61 -189, -133, -173, 75, 168, 112, -195, 10.649

89, -188, 107, -197, -162, -180, 51,

-142

40/109 -57 → 60 -100, -165, -64, -78, -199, 83, 68, -143 36.098

41/109 -5 → 53 -183, -84 77.231

42/109 66 → 46 -183, -84 39.190

43/109 -64 → 68 -78, -199, 83 60.218

44/109 61 → 73 147, -71, 122, 80, -155, -97, -130, 5.897

-118, -154, 135, -134, 128, -140, -131,

-158, -94, -148, -70, 192, -85, -104, 86

45/109 58 → -56 100, 88, -108, 200, 78, 200, 98, -62, 11.685

-111

46/109 11 → 18 -127, -98, -200, -78, -200, 108, -161, 2.466

-156, 91, -98, -200, 108, -161, -156,

91, -98, -200, 108, -161, -156, 91, -98,

-200, -78, -200, 108

47/109 47 → 50 105, 121, -102 68.372

48/109 33 → -12 -49 86.157

49/109 48 → -28 105, -120, -102 80.178

50/109 62 → -58 -98, -200, -78, -200, 108, -88, -100 67.188

51/109 67 → 39 90 60.275

52/109 -15 → 63 0.918

53/109 34 → 9 2.537

54/109 -29 → 69 -150, -74, -86, 163, -170, 92, -145 44.999

55/109 61 → -36 147, -71, 122, 80, -155, -97, -130, 49.448

-118, -154, 135, -134, 128, -140, -131,

-158, -94, -148, -70, 192, -85, -104,

86, 73, 150

56/109 -57 → 68 -100, -165, -64, -78, -199, 83 4.142

57/109 -15 → 42 90 76.624

58/109 72 → -41 137, -99, -144 23.010

59/109 -60 → 64 143, -68, -83, 199, 78 26.712

60/109 34 → 45 -92 68.758

61/109 55 → 66 -100, -165 61.140

62/109 -26 → 67 2.113

63/109 71 → -70 2.473

64/109 71 → 8 1.407

65/109 39 → 12 3.941

66/109 -8 → 70 1.218

67/109 27 → 21 145, -92, 170 81.538

68/109 28 → 16 2.481

69/109 71 → 35 2.131

70/109 29 → 39 184, 162, 197, -107, -194, 191, -89, 42.420

196, -112, -171, -75, 173, 133, 189, 67,

90

71/109 -11 → 44 -98, -200, -78, -199, 83, 179, 127 5.720

72/109 62 → 68 -98, -200, -78, -199, 83 8.589

73/109 -17 → 30 149, -185, 149, -185, 149 81.708

74/109 40 → 25 -111 80.570

75/109 24 → -5 139, -175, 139 74.751

76/109 44 → 23 110 57.658

77/109 22 → 33 2.435

78/109 -2 → 52 1.462

79/109 25 → 43 -114, 159, 115, -79, -114 8.767

80/109 64 → 57 165, 100 42.657

81/109 31 → 16 182, -173, 75, 169, 153, -196, 89, -190, 9.528

193, 107, -197, -162, -184, -172, -178,

167

82/109 30 → -21 2.257

83/109 57 → 56 138, 87, 138 35.179

84/109 50 → 35 110 69.173

85/109 55 → 46 -100, -165, 66, -183, -84 41.131

86/109 72 → -16 137, -99, -144, -187, -117, 166 3.486

87/109 -27 → 48 -116 80.569

88/109 72 → -19 137, -99, -144, -187, -117 1.281

89/109 53 → 55 -142 70.698

90/109 1 → 72 137 37.380

91/109 -28 → 47 -116 82.326

92/109 60 → 57 165, 100 7.993

93/109 74 → 29 150 76.002

94/109 51 → 61 -142 80.467

95/109 26 → -6 143 83.065

96/109 8 → 33 157 82.005

97/109 71 → -61 -147 43.355

98/109 7 → 5 2.619

99/109 73 → -36 150 62.026

100/109 17 → -15 157 76.565

101/109 56 → 62 111 48.475

102/109 32 → -10 96.589

103/109 68 → 60 -143 53.175

104/109 35 → -4 174 83.133

105/109 60 → 58 165, 100 68.338

106/109 41 → -1 144, 99, -137, -72, -137 40.145

107/109 36 → 70 -150, -73, -86, 104, 85, -192 43.235

108/109 16 → 31 -166, 117, 187, 144, -125, -103, 123, 43.161

-186, 176, 151, 181, 63, -181, -151,

-176, -123, 103, 109, -117, 166, -160

109/109 70 → -61 148, 94, 158, 131, 140, -128, 134, -135, 22.036

154, 118, 130, 97, 155, -80, -122, 71,

-147

Applying bridges (2022-07-15 00:34:55)

--------------------------------------

Unicycler now applies to the graph in decreasing order of quality. This ensures that when multiple, contradictory bridges exist, the most supported option is used.

Bridge type Start → end Path Quality

simple long read -30 → 17 -149, 185, -149, 185, -149 100.000

simple long read 12 → -33 49 99.779

miniasm 32 → -10 99.351

miniasm 4 → -35 -174 99.290

simple long read -61 → -51 142 98.760

simple long read 26 → -6 143 98.484

simple long read 17 → -15 157 98.433

simple long read 8 → 33 157 98.176

simple long read -47 → 28 116 97.678

simple long read 74 → 29 150 95.723

simple long read -48 → 27 116 95.517

simple long read -25 → -40 111 95.468

miniasm 60 → 58 165, 100 92.606

simple long read -15 → 42 90 91.505

miniasm 27 → 21 145, -92, 170 90.643

simple long read 5 → -24 -139, 175, -139 89.614

miniasm -40 → -43 -84 88.521

miniasm 28 → -48 102, 120, -105 87.506

simple long read -55 → -53 142 86.382

miniasm 34 → 45 -92 85.597

long read 18 → -3 -108, 200, 78, 199, 78, 200, 98, -91, 84.465

156

miniasm 45 → 36 192, -85, -106 84.068

long read 21 → -2 93, -199, 83, 179, 127, -101, -98, -200, 83.332

108

simple long read 50 → 35 110 83.183

long read -6 → 32 -127, -179, -83, 199, -93 82.940

long read 52 → -25 -159, 114, 79, -115, -159, 114 81.951

long read 11 → 4 -127, -98, -200, -78, -200, 108, -161, 81.860

-156

long read -1 → 14 -127, -179, -83, 199, 78, 199, -93 81.776

long read -24 → 34 93, -199, 83, 179, 127 81.458

miniasm -9 → -19 -151, -177, 186, 124, 103, 109, -117 81.012

long read -43 → 52 114, -77, -115, -159, 114 80.654

long read 37 → 38 93, -199, 83, 179, 127 80.253

long read -10 → 16 -133, -173, 75, 169, 153, -196, 89, 80.015

-190, 194, 107, -197, -162, -184, -172,

-178, 167

long read 42 → 8 172, 184, 162, 197, -107, 188, -89, 195, 79.993

-112, -168, -75, 173, 141

long read -20 → 37 146, 180, 162, 197, -107, 188, -89, 195, 79.896

-112, -168, -75, 173, -182

long read 23 → 11 -108, 200, 78, 200, 98 79.529

long read 14 → 13 -108, 200, 78, 200, 98 78.925

simple long read 73 → -36 150 78.200

long read 39 → -31 -167, 178, 172, 184, 162, 197, -107, 78.132

-193, 190, -89, 195, 126, -171, -75,

173, -182

simple long read 56 → 62 111 77.426

miniasm -7 → -30 -151, -177, 186, -123, 103, 109, -117, 77.379

166, -160

long read -5 → 53 -183, -84 77.231

miniasm -73 → 70 -86, 104, 85, -192 76.981

long read 20 → -7 -152, -173, 75, 169, -126, -195, 89, 76.811

-190, 193, 107, -198, -162, -180, -146

long read 46 → 12 -98, -200, -78, -200, 108 76.561

simple long read 67 → 39 90 75.254

miniasm -64 → 68 -78, -199, 83 75.249

long read -18 → 54 -108, 200, 78, 200, 98, -113, 93, -199, 74.650

83, 179, 127

long read 70 → 71 148, 94, 158, 131, 140, -128, 134, -135, 74.295

154, 118, 130, 97, 155, -80, -122

miniasm -2 → -54 -85, -106 74.169

miniasm -50 → -47 102, -121, -105 73.373

simple long read -60 → -68 143 73.062

miniasm -66 → -55 165, 100 72.706

long read 22 → 26 -152, -173, 75, 169, -126, -195, 89, 72.649

-191, 194, 107, -198, -162, -184, -172,

-178

miniasm -3 → 41 -164, -82, 174, 164 71.493

long read 59 → 22 -108, 200, 78, 200, 98 70.799

long read 9 → 51 -189, -133, -173, 75, 169, -126, -195, 69.520

89, -188, 107, -197, -162, -180

simple long read 44 → 23 110 69.085

miniasm -74 → 69 -86, 163, -170, 92, -145 68.283

long read 62 → -58 -98, -200, -78, -200, 108, -88, -100 67.188

long read 13 → 59 -127, -179, -83, 199, 78, 199, -93 64.377

long read 29 → 67 184, 162, 197, -107, -194, 191, -89, 63.086

196, -112, -171, -75, 173, 133, 189

long read -19 → 44 93, -199, -78, -199, 83, 179, 127 60.657

long read 69 → -38 148, 95, 158, 132, 140, 129, 134, 136, 59.917

154, 119, 130, -96, 155, -81, -122, 76,

-147

miniasm -57 → -64 -100, -165 55.475

miniasm 71 → -61 -147 55.316

simple long read 57 → 56 138, 87, 138 52.689

miniasm -72 → -1 -137 46.273

miniasm -46 → -66 84, 183 43.834

long read 16 → 63 -166, 117, 187, 144, -125, -103, 123, 43.161

-186, 176, 151, 181

long read 63 → 31 -181, -151, -176, -123, 103, 109, -117, 43.161

166, -160

long read 41 → -72 144, 99, -137 40.145

Saving /data5/xuhao/3data/wang1_wang9/01.Cleandata/wang1/wang1_hy/004_bridges_applied.gfa

Bridged assembly graph (2022-07-15 00:34:57)

--------------------------------------------

The assembly is now mostly finished and no more structural changes will be made. Ideally the assembly graph should now have one contig per replicon and no erroneous contigs (i.e a complete assembly). If there are more contigs, then the assembly is not complete.

Saving /data5/xuhao/3data/wang1_wang9/01.Cleandata/wang1/wang1_hy/005_final_clean.gfa

Component Segments Links Length N50 Longest segment Status

total 4 4 5,452,357 5,232,708 5,232,708

1 1 1 5,232,708 5,232,708 5,232,708 complete

2 1 1 149,719 149,719 149,719 complete

3 1 1 65,148 65,148 65,148 complete

4 1 1 4,782 4,782 4,782 complete

Polishing assembly with Pilon (2022-07-15 00:34:57)

---------------------------------------------------

Unicycler now conducts multiple rounds of Pilon in an attempt to repair any remaining small-scale errors with the assembly.

Aligning reads to find appropriate insert size range...

Insert size 1st percentile: 221

Insert size 99th percentile: 565

Pilon polish round 1

Total number of changes: 76

Pilon polish round 2

Total number of changes: 54

Pilon polish round 3

Total number of changes: 13

Pilon polish round 4

Total number of changes: 4

Pilon polish round 5

No Pilon changes

Pilon polish round 6

No Pilon changes

Saving /data5/xuhao/3data/wang1_wang9/01.Cleandata/wang1/wang1_hy/006_polished.gfa

Rotating completed replicons (2022-07-15 02:11:30)

--------------------------------------------------

Any completed circular contigs (i.e. single contigs which have one link connecting end to start) can have their start position changed without altering the sequence. For consistency, Unicycler now searches for a starting gene (dnaA or repA) in each such contig, and if one is found, the contig is rotated to start with that gene on the forward strand.

Segment Length Depth Starting gene Position Strand Identity Coverage

1 5,232,707 1.00x UniRef90_Q8XBZ3 1,079,489 reverse 95.3% 100.0%

2 149,719 1.30x UniRef90_J5VKS5 101,844 reverse 99.7% 100.0%

3 65,148 0.76x UniRef90_Q6U5J6 38,076 forward 97.6% 100.0%

4 4,782 11.69x none found

Saving /data5/xuhao/3data/wang1_wang9/01.Cleandata/wang1/wang1_hy/007_rotated.gfa

Assembly complete (2022-07-15 02:15:31)

---------------------------------------

Saving /data5/xuhao/3data/wang1_wang9/01.Cleandata/wang1/wang1_hy/assembly.gfa

Saving /data5/xuhao/3data/wang1_wang9/01.Cleandata/wang1/wang1_hy/assembly.fasta

Wang9

Starting Unicycler (2022-07-15 08:03:44)

----------------------------------------

Welcome to Unicycler, an assembly pipeline for bacterial genomes. Since you provided both short and long reads, Unicycler will perform a hybrid assembly. It will first use SPAdes to make a short-read assembly graph, and then it will use various methods to scaffold that graph with the long reads.

For more information, please see https://github.com/rrwick/Unicycler

Command: ./unicycler-runner.py --spades_path /home/xhao/software/SPAdes/bin/spades.py --racon_path /home/xhao/miniconda2/bin/racon --bowtie2_path /home/xhao/software/bowtie2-2.4.2-linux-x86_64/bowtie2 --bowtie2_build_path /home/xhao/software/bowtie2-2.4.2-linux-x86_64/bowtie2-build --pilon_path /home/xhao/software/pilon-1.22.jar -1 /data5/xuhao/3data/wang1_wang9/illumina_Result/01.Cleandata/wang9/wang9.L350_FDSW220003795-2r_1.fq.clean.gz -2 /data5/xuhao/3data/wang1_wang9/illumina_Result/01.Cleandata/wang9/wang9.L350_FDSW220003795-2r_2.fq.clean.gz -l /data5/xuhao/3data/wang1_wang9/01.Cleandata/wang9/barcode08.fastq -o /data5/xuhao/3data/wang1_wang9/01.Cleandata/wang9/wang9_hy

Unicycler version: v0.4.9

Using 8 threads

Making output directory:

/data5/xuhao/3data/wang1_wang9/01.Cleandata/wang9/wang9_hy

Dependencies:

Program Version Status

spades.py 3.13.0 good

racon 1.4.13 good

makeblastdb 2.9.0+ good

tblastn 2.9.0+ good

bowtie2-build 2.4.2 good

bowtie2 2.4.2 good

samtools 1.9 good

java 1.8.0_282 good

pilon 1.22 good

bcftools not used

SPAdes read error correction (2022-07-15 08:05:45)

--------------------------------------------------

Unicycler uses the SPAdes read error correction module to reduce the number of errors in the short read before SPAdes assembly. This can make the assembly faster and simplify the assembly graph structure.

Command: /home/xhao/software/SPAdes/bin/spades.py -1 /data5/xuhao/3data/wang1_wang9/illumina_Result/01.Cleandata/wang9/wang9.L350_FDSW220003795-2r_1.fq.clean.gz -2 /data5/xuhao/3data/wang1_wang9/illumina_Result/01.Cleandata/wang9/wang9.L350_FDSW220003795-2r_2.fq.clean.gz -o /data5/xuhao/3data/wang1_wang9/01.Cleandata/wang9/wang9_hy/spades_assembly/read_correction --threads 8 --only-error-correction --phred-offset 33

Corrected reads:

/data5/xuhao/3data/wang1_wang9/01.Cleandata/wang9/wang9_hy/spades_assembly/corrected_1.fastq.gz

/data5/xuhao/3data/wang1_wang9/01.Cleandata/wang9/wang9_hy/spades_assembly/corrected_2.fastq.gz

Choosing k-mer range for assembly (2022-07-15 08:41:30)

-------------------------------------------------------

Unicycler chooses a k-mer range for SPAdes based on the length of the input reads. It uses a wide range of many k-mer sizes to maximise the chance of finding an ideal assembly.

SPAdes maximum k-mer: 127

Median read length: 150

K-mer range: 27, 47, 63, 77, 89, 99, 107, 115, 121, 127

SPAdes assemblies (2022-07-15 08:43:27)

---------------------------------------

Unicycler now uses SPAdes to assemble the short reads. It scores the assembly graph for each k-mer using the number of contigs (fewer is better) and the number of dead ends (fewer is better). The score function is 1/(c*(d+2)), where c is the contig count and d is the dead end count.

K-mer Contigs Dead ends Score

27 1,245 1 2.68e-04

47 550 1 6.06e-04

63 454 1 7.34e-04

77 388 1 8.59e-04

89 307 1 1.09e-03

99 291 1 1.15e-03

107 288 1 1.16e-03

115 279 1 1.19e-03

121 270 1 1.23e-03

127 258 1 1.29e-03 ← best

Read depth filter: removed 9 contigs totalling 5825 bp

Deleting /data5/xuhao/3data/wang1_wang9/01.Cleandata/wang9/wang9_hy/spades_assembly/

Determining graph multiplicity (2022-07-15 09:02:24)

----------------------------------------------------

Multiplicity is the number of times a sequence occurs in the underlying sequence. Single-copy contigs (those with a multiplicity of one, occurring only once in the underlying sequence) are particularly useful.

Saving /data5/xuhao/3data/wang1_wang9/01.Cleandata/wang9/wang9_hy/001_best_spades_graph.gfa

Cleaning graph (2022-07-15 09:02:24)

------------------------------------

Unicycler now performs various cleaning procedures on the graph to remove overlaps and simplify the graph structure. The end result is a graph ready for bridging.

Graph overlaps removed

Removed zero-length segments:

155, 157, 158, 160, 161, 162, 163, 165, 166, 168, 170, 171, 178, 182, 185, 193, 194, 201, 202, 203, 204, 205, 206, 213, 228, 230, 251

Removed zero-length segments:

156, 225, 234, 237, 239, 257

Merged small segments:

223, 224, 227, 229, 231, 232, 233, 235, 236, 240, 241, 242, 243, 244, 245, 246, 247, 248, 249, 250, 252, 253, 254, 255, 256, 258

Saving /data5/xuhao/3data/wang1_wang9/01.Cleandata/wang9/wang9_hy/002_overlaps_removed.gfa

Unicycler now selects a set of anchor contigs from the single-copy contigs. These are the contigs which will be connected via bridges to form the final assembly.

72 anchor segments (5,407,869 bp) out of 202 total segments (5,481,463 bp)

Creating SPAdes contig bridges (2022-07-15 09:02:25)

----------------------------------------------------

SPAdes uses paired-end information to perform repeat resolution (RR) and produce contigs from the assembly graph. SPAdes saves the graph paths corresponding to these contigs in the contigs.paths file. When one of these paths contains two or more anchor contigs, Unicycler can create a bridge from the path.

Bridge

Start Path End quality

-36 147 60 50.8

-31 -173 → -114 → 126 42 25.6

-25 120 45 62.6

-7 46 13 3.5

1 136 71 37.1

23 139 → -174 → 139 6 25.7

28 151 74 61.3

33 151 73 52.7

44 -120 26 63.2

56 147 57 36.9

57 131 → 101 → -134 → 129 → -140 → -89 → 148 68 10.7

59 153 69 38.7

60 131 → -102 → -134 → -130 → -140 → 88 → 148 67 10.7

61 -72 -66 7.0

71 136 → 97 → 143 2 9.3

Creating loop unrolling bridges (2022-07-15 09:02:25)

-----------------------------------------------------

When a SPAdes contig path connects an anchor contig with the middle contig of a simple loop, Unicycler concludes that the sequences are contiguous (i.e. the loop is not a separate piece of DNA). It then uses the read depth of the middle and repeat contigs to guess the number of times to traverse the loop and makes a bridge.

Loop count Loop count Loop Bridge

Start Repeat Middle End by repeat by middle count quality

-6 -139 174 -23 0.92 0.65 1 38.8

-9 150 184 29 0.03 0.77 1 16.6

Loading reads (2022-07-15 09:02:25)

-----------------------------------

2,361,594 / 2,361,594 (100.0%) - 16,942,884,655 bp

Assembling contigs and long reads with miniasm (2022-07-15 09:06:06)

--------------------------------------------------------------------

Unicycler uses miniasm to construct a string graph assembly using both the short read contigs and the long reads. It will then use the resulting string graph to produce bridges between contigs. This method requires decent coverage of long reads and therefore may not be fruitful if long reads are sparse. However, it does not rely on the short read assembly graph having good connectivity and is able to bridge an assembly graph even when it contains many dead ends.

Unicycler uses two types of "reads" as assembly input: anchor contigs from the short-read assembly and actual long reads which overlap two or more of these contigs. It then assembles them with miniasm.

Aligning long reads to graph using minimap

Saving to /data5/xuhao/3data/wang1_wang9/01.Cleandata/wang9/wang9_hy/miniasm_assembly/01_assembly_reads.fastq:

71 short-read contigs

259,434 long reads

Finding overlaps with minimap...

success

399,010,213 overlaps

Assembling reads with miniasm...

success

381 segments, 380 links

Saving /data5/xuhao/3data/wang1_wang9/01.Cleandata/wang9/wang9_hy/miniasm_assembly/11_branching_paths_removed.gfa

Merging segments into unitigs:

9 circular unitigs

1 linear unitig

total size = 5,827,882 bp

Saving /data5/xuhao/3data/wang1_wang9/01.Cleandata/wang9/wang9_hy/miniasm_assembly/12_unitig_graph.gfa

Polishing miniasm assembly with Racon (2022-07-15 11:22:32)

-----------------------------------------------------------

Unicycler now uses Racon to polish the miniasm assembly. It does multiple rounds of polishing to get the best consensus. Circular unitigs are rotated between rounds such that all parts (including the ends) are polished well.

Saving to /data5/xuhao/3data/wang1_wang9/01.Cleandata/wang9/wang9_hy/miniasm_assembly/racon_polish/polishing_reads.fastq:

71 short-read contigs

2,361,594 long reads

Polish Assembly Mapping

round size quality

begin 5,827,882 1,674,508.32

1 5,851,700 1,842,921.32

Best polish: /data5/xuhao/3data/wang1_wang9/01.Cleandata/wang9/wang9_hy/miniasm_assembly/racon_polish/006_rotated.fasta

Saving /data5/xuhao/3data/wang1_wang9/01.Cleandata/wang9/wang9_hy/miniasm_assembly/13_racon_polished.gfa

Saving /data5/xuhao/3data/wang1_wang9/01.Cleandata/wang9/wang9_hy/003_long_read_assembly.gfa

Contigs in the short-read assembly graph which end in dead ends may contain bogus sequence near the dead end. Unicycler therefore uses the read clipping values from the miniasm assembly to trim these dead ends to only the parts which aligned well to long reads.

No dead ends required trimming.

Unicycler now places the single copy contigs back into the unitig graph. This serves two purposes: a) it replaces long read assembly sequences (which may be error prone) with Illumina assembly sequence (which is probably quite accurate), improving the assembly quality, and b) it defines inter-contig sequences for use in building bridges.

Searching for contigs using 5,000 bp of contig ends.

Contig Result Start pos End pos Strand

1 found in unitig 1 1885785 2536693 -

2 found in unitig 1 1535798 1881058 -

3 found in unitig 1 2854793 3192413 +

4 found in unitig 1 1079307 1416897 -

5 not found

6 found in unitig 1 4728717 4997991 -

7 found in unitig 1 4334778 4549284 -

8 found in unitig 1 3835900 4047584 -

9 found in unitig 1 4105306 4302123 +

10 found in unitig 1 3195044 3371611 +

11 found in unitig 1 796 159125 -

12 found in unitig 1 360594 474058 +

13 not found

14 found in unitig 1 2647908 2753087 -

15 found in unitig 1 2541583 2644420 +

16 found in unitig 1 3377114 3478614 -

17 found in unitig 1 1437909 1530024 -

18 found in unitig 1 161040 250939 +

19 found in unitig 1 3745153 3830384 +

20 found in unitig 1 989677 1074747 +

21 found in unitig 1 2770629 2849308 -

22 found in unitig 1 278611 357105 -

23 found in unitig 1 4998425 5074222 -

24 found in unitig 3 -42935 28706 -

25 found in unitig 1 918091 988657 +

26 found in unitig 1 833111 899536 +

27 not found

28 found in unitig 1 3592020 3653749 +

29 found in unitig 1 4049689 4104835 -

30 found in unitig 1 3489199 3543912 +

31 found in unitig 1 5076842 5120582 -

32 found in unitig 2 178605 218318 -

33 found in unitig 1 5145219 5183336 +

34 found in unitig 2 32745 70279 -

35 found in unitig 1 3707127 3739569 +

36 not found

37 found in unitig 1 3549371 3581249 +

38 found in unitig 3 76827 103979 +

39 found in unitig 1 4303046 4329158 +

40 found in unitig 3 50327 75524 -

41 not found

42 not found

43 not found

44 found in unitig 1 814207 832806 +

45 found in unitig 1 901059 917790 -

47 found in unitig 1 797474 812680 -

48 found in unitig 3 31930 46620 -

49 found in unitig 1 5211318 5225679 +

50 found in unitig 1 4714376 4727374 -

51 found in unitig 1 1418692 1431457 +

52 found in unitig 1 4703270 4713330 +

53 found in unitig 2 71654 81162 +

54 found in unitig 1 2757978 2767135 +

55 found in unitig 2 160083 167988 +

56 found in unitig 1 5203186 5210121 -

57 found in unitig 1 5196515 5203038 -

58 found in unitig 1 3480654 3487074 +

59 found in unitig 2 21863 28201 -

60 found in unitig 8 2390 8191 -

61 found in unitig 2 2675 8111 -

62 found in unitig 2 172453 177407 -

64 found in unitig 1 4698234 4702592 +

65 found in unitig 1 3582171 3586378 -

66 found in unitig 2 220539 224598 +

67 found in unitig 8 23699 27699 -

68 found in unitig 1 5189807 5193683 -

69 found in unitig 2 17970 21729 -

70 found in unitig 2 87026 90657 +

71 found in unitig 1 1882190 1885561 -

73 found in unitig 1 5183476 5186633 +

74 found in unitig 8 18163 21308 +

Searching for contigs using 2,500 bp of contig ends.

Contig Result Start pos End pos Strand

5 found in unitig 1 477806 797009 +

13 not found

27 found in unitig 2 92019 159263 +

36 not found

41 not found

42 found in unitig 1 5121307 5143414 +

43 not found

Searching for contigs using 1,000 bp of contig ends.

Contig Result Start pos End pos Strand

13 not found

36 not found

41 not found

43 not found

Searching for contigs using 500 bp of contig ends.

Contig Result Start pos End pos Strand

13 found in unitig 1 4564970 4671550 +

36 found in unitig 1 3672190 3704510 +

41 found in unitig 1 255828 278148 -

43 not found

Saving /data5/xuhao/3data/wang1_wang9/01.Cleandata/wang9/wang9_hy/miniasm_assembly/15_contigs_placed.gfa

Creating miniasm/Racon bridges (2022-07-15 19:58:35)

----------------------------------------------------

Now that the miniasm/Racon string graph is complete, Unicycler will use it to build bridges between anchor segments.

Start → end Best path Quality

1/70 -11 → 18 -152, 193, 164, 92, 111, 122 83.197

2/70 -45 → 25 -120 96.150

3/70 25 → 20 137, -173, -113, 126, 166 90.860

4/70 20 → -4 -94, -202, -82, -128, 98, 96, -201, -110 71.714

5/70 -4 → 51 86, -108 75.557

6/70 51 → -17 128, 82, 202, 94, 117, 96, -201, -78, 59.866

-201, -110

7/70 -17 → -2 110, 201, 78, 201, 78, 201, -96 67.967

8/70 -2 → -71 -143, -97, -136 44.839

9/70 -71 → -1 -136 57.401

10/70 -1 → 15 128, 82, 202, 78, 202, 94 69.417

11/70 15 → -14 110, 201, 78, 201, -96 74.235

12/70 18 → -41 -94, -202, -78, -202, -82, -128 54.676

13/70 -14 → 54 128, 82, 202, 78, 202, 94 57.819

14/70 54 → -21 110, 201, 78, 201, -96 68.209

15/70 -21 → 3 -154, 165, 75, -170, 132, 181, -90, 190, 65.114

195, -109, -199, -160, -183, -167, -175

16/70 3 → 10 128, 82, 202, 94 80.425

17/70 10 → -16 133, 165, 75, -170, -159, 182, -90, 68.910

-189, 195, -109, -200, -160, -183, -167,

-175, -163

18/70 -16 → 58 -162, -122, -186, -143, -125, -92, -164, 34.231

-192, 152, -177

19/70 58 → 30 177, -152, 192, 176, 92, 111, 122, 162, 35.737

-157

20/70 30 → 37 179, 165, 75, -171, 132, 181, -90, -189, 68.142

-194, -109, -200, -160, -183, -167,

-175, -163

21/70 37 → -65 -91 76.939

22/70 -65 → 28 -188, 133, 165, 75, -171, 121, -158, 62.684

-196, 182, -90, 190, 195, -109, -200,

-160, -183

23/70 -41 → -22 -112 75.205

24/70 28 → 36 151, 74, -87, 161, -166, -126, 113, 172, 46.154

-137, -67, -148, -88, 140, 130, 134,

102, -131, -60, -147

25/70 36 → 35 128, 82, 202, 94 79.154

26/70 35 → 19 179, 165, 75, -116, -158, 181, -90, 185, 67.618

-109, -200, -160, 178, 198, -145

27/70 19 → -8 -154, 165, 75, -170, 132, 181, -90, 66.130

-189, -194, -109, -199, -160, 178, 197,

-145

28/70 -8 → -29 -152, 193, 164, 92, 111, 122, 162, -157 80.095

29/70 -29 → 9 -150, -184, -150, -184, -150 95.368

30/70 9 → 39 91 87.110

31/70 39 → -7 167, 183, 160, 200, 109, -185, 90, -181, 65.670

158, 116, -75, -165, -142

32/70 -7 → 13 46 49.037

33/70 13 → 64 110, 201, 78, 201, -96, 43, -85, 180 30.775

34/70 -22 → 12 110, 201, 78, 201, -96 72.636

35/70 64 → 52 168, -103 82.666

36/70 52 → -50 115, 141, -127, -135 85.981

37/70 -50 → -6 -85, 180 87.163

38/70 -6 → -23 -139, 174, -139 90.301

39/70 -23 → -31 -94, -202, -82, -128 79.999

40/70 -31 → 42 -173, -114, 126 90.834

41/70 42 → 33 191, 86, -108 81.800

42/70 33 → 73 151 82.208

43/70 73 → -68 -87, 104, -86, -191 77.573

44/70 -68 → -57 -148, 89, 140, -129, 134, -101, -131 79.890

45/70 12 → 5 128, 96, -201, -78, -201, -110 74.969

46/70 -57 → -56 -147 57.484

47/70 -56 → 49 105, 141, 107 89.717

48/70 49 → -11 -197, -178, 160, 200, 109, -185, 90, 59.986

-181, -132, 170, -75, -165, -133, 188

49/70 -61 → -69 72, -93, 81, 144, -79, -155, 84 16.162

50/70 -69 → -59 -153 60.433

51/70 -59 → -34 60.286

52/70 -34 → 53 -83 53.426

53/70 53 → 70 46.969

54/70 70 → 27 83 30.651

55/70 27 → 55 103, -169, -149 91.640

56/70 5 → -47 112 83.054

57/70 55 → -62 -76, 149, 169, -103, 187, 127, -141, 68.710

-115, -146

58/70 -62 → -32 135, 127, -141, -115, -146 82.196

59/70 -32 → 66 149, 169, -103, 138, 105, 141, 107 69.210

60/70 66 → -61 72 54.879

61/70 -24 → -48 -118, 156, -119, 80, -118, 156 77.916

62/70 -48 → -40 -118, 156, -119, 77, -118 75.713

63/70 -40 → 38 -85 88.217

64/70 38 → -24 -76 79.102

65/70 -47 → 44 99, -124, 106 75.287

66/70 -60 → 74 43.760

67/70 74 → -67 -87, 161, -166, -126, 113, 172, -137 72.434

68/70 -67 → -60 -148, -88, 140, 130, 134, 102, -131 79.754

69/70 44 → 26 -120 97.083

70/70 26 → -45 99, -123, 106 87.489

Creating simple long read bridges (2022-07-15 20:02:08)

-------------------------------------------------------

Unicycler uses long read alignments (from minimap) to resolve simple repeat structures in the graph. This takes care of some "low-hanging fruit" of the graph simplification.

Aligning long reads to graph using minimap

Two-way junctions are defined as cases where two graph contigs (A and B) join together (C) and then split apart again (D and E). This usually represents a simple 2-copy repeat, and there are two possible options for its resolution: (A→C→D and B→C→E) or (A→C→E and B→C→D). Each read which spans such a junction gets to "vote" for option 1, option 2 or neither. Unicycler creates a bridge at each junction for the most voted for option.

Op. 1 Op. 2 Neither Final Bridge

Junction Option 1 Option 2 votes votes votes op. quality

120 -26 → 120 → -44, -26 → 120 → 45, 3824 3 95 1 96.5

-25 → 120 → 45 -25 → 120 → -44

83 -53 → 83 → 27, -53 → 83 → 34, 0 1778 32 2 34.0

70 → 83 → 34 70 → 83 → 27

147 -36 → 147 → 57, -36 → 147 → 60, 0 5808 63 2 57.7

56 → 147 → 60 56 → 147 → 57

91 9 → 91 → -37, 65 9 → 91 → 39, 65 2 4843 68 2 82.5

→ 91 → 39 → 91 → -37

151 28 → 151 → 73, 28 → 151 → 74, 85 6044 57 2 81.4

33 → 151 → 74 33 → 151 → 73

112 5 → 112 → -47, 5 → 112 → 41, 22 4309 1 72 1 77.3

22 → 112 → 41 → 112 → -47

Simple loops are parts of the graph where two contigs (A and B) are connected via a repeat (C) which loops back to itself (via D). It is possible to traverse the loop zero times (A→C→B), one time (A→C→D→C→B), two times (A→C→D→C→D→C→B), etc. Long reads which span the loop inform which is the correct number of times through. In this step, such reads are found and each is aligned against alternative loop counts. A reads casts its "vote" for the loop count it agrees best with, and Unicycler creates a bridge using the most voted for count.

Read Loop Bridge

Start Repeat Middle End count Read votes count quality

-9 150 184 29 2679 0 loops: 5 votes 2 99.1

1 loop: 9 votes

2 loops: 2662

votes 3 loops:

3 votes

-7 46 13 125 0 loops: 125 votes 0 99.8

-6 -139 174 -23 2184 bad: 1 vote 0 1 94.0

loops: 2 votes

1 loop: 2180 votes

2 loops: 1 vote

Determining low score threshold (2022-07-15 20:38:00)

-----------------------------------------------------

Before conducting semi-global alignment of the long reads to the assembly graph, Unicycler must determine a minimum alignment score threshold such that nonsense alignments are excluded. To choose a threshold automatically, it examines alignments between random sequences and selects a score a few standard deviations above the mean.

Automatically choosing a threshold using random alignment scores.

Random alignment mean score: 61.66

standard deviation: 1.31

Low score threshold: 61.66 + (7 x 1.31) = 70.86

Aligning reads (2022-07-15 20:43:22)

------------------------------------

2,361,594 / 2,361,594 (100.0%)

Read alignment summary (2022-07-18 11:49:19)

--------------------------------------------

Total read count: 2,361,594

Fully aligned reads: 2,177,570

Partially aligned reads: 171,544

Unaligned reads: 12,480

Total bases aligned: 16,895,855,424 bp

Mean alignment identity: 91.1%

Deleting /data5/xuhao/3data/wang1_wang9/01.Cleandata/wang9/wang9_hy/read_alignment/

Building long read bridges (2022-07-18 11:49:56)

------------------------------------------------

Unicycler uses the long read alignments to produce bridges between anchor segments. These bridges can be formed using as few as one long read, giving Unicycler the ability to bridge the graph even when long-read depth is low.

Start → end Best path Quality

1/357 38 → -24 -76 80.578

2/357 60 → 67 131, -102, -134, -130, -140, 88, 148 76.715

3/357 48 → 24 -156, 118, -80, 119, -156, 118 81.451

4/357 40 → 48 118, -77, 119, -156, 118 80.539

5/357 74 → -67 -87, 161, -166, -126, 113, 172, -137 65.634

6/357 30 → 37 179, 165, 75, -171, 132, 181, -90, -189, 78.817

-194, -109, -200, -160, -183, -167,

-175, -163

7/357 16 → -10 163, 175, 167, 183, 160, 200, 109, -195, 78.860

189, 90, -182, 159, 170, -75, -165, -133

8/357 13 → 43 110, 201, 78, 201, -96 75.079

9/357 -28 → 65 183, 160, 200, 109, -195, -190, 90, 70.543

-182, 196, 158, -121, 171, -75, -165,

-133, 188

10/357 19 → -8 -154, 165, 75, -170, 132, 181, -90, 78.871

-189, -194, -109, -199, -160, 178, 197,

-145

11/357 15 → -14 110, 201, 78, 201, -96 80.420

12/357 -3 → 21 175, 167, 183, 160, 199, 109, -195, 77.436

-190, 90, -181, -132, 170, -75, -165,

154

13/357 35 → 19 179, 165, 75, -116, -158, 181, -90, 185, 79.015

-109, -200, -160, 178, 198, -145

14/357 39 → -7 167, 183, 160, 200, 109, -185, 90, -181, 78.176

158, 116, -75, -165, -142

15/357 54 → -21 110, 201, 78, 201, -96 72.267

16/357 36 → 35 128, 82, 202, 94 80.427

17/357 -14 → 54 128, 82, 202, 78, 202, 94 66.094

18/357 -1 → 15 128, 82, 202, 78, 202, 94 81.567

19/357 57 → 68 131, 101, -134, 129, -140, -89, 148 76.934

20/357 3 → 10 128, 82, 202, 94 82.440

21/357 49 → -11 -197, -178, 160, 200, 109, -185, 90, 69.241

-181, -132, 170, -75, -165, -133, 188

22/357 29 → 8 157, -162, -122, -111, -92, -164, -193, 73.468

152

23/357 73 → -68 -87, 104, -86, -191 69.410

24/357 41 → -18 128, 82, 202, 78, 202, 94 63.329

25/357 -12 → 22 96, -201, -78, -201, -110 78.400

26/357 31 → 23 128, 82, 202, 94 81.649

27/357 12 → 5 128, 96, -201, -78, -201, -110 82.411

28/357 -16 → 58 -162, -122, -186, -143, -125, -92, -164, 31.952

-192, 152, -177

29/357 -38 → 40 85 81.334

30/357 58 → 30 177, -152, 192, 176, 92, 111, 122, 162, 31.170

-157

31/357 20 → -4 -94, -202, -82, -128, 98, 96, -201, -110 82.172

32/357 43 → 64 -85, 180 46.278

33/357 42 → 33 191, 86, -108 78.414

34/357 2 → 17 96, -201, -78, -202, -78, -201, -110 82.166

35/357 -11 → 18 -152, 193, 164, 92, 111, 122 72.649

36/357 -4 → 51 86, -108 72.095

37/357 6 → 50 -180, 85 80.375

38/357 65 → -37 91 66.404

39/357 -49 → 56 -107, -141, -105 79.988

40/357 45 → -26 -106, 123, -99 79.682

41/357 -44 → 47 -106, 124, -99 69.747

42/357 52 → -50 115, 141, -127, -135 74.942

43/357 9 → 39 91 78.279

44/357 71 → 2 136, 97, 143 39.065

45/357 34 → 59 22.866

46/357 66 → -61 72 49.847

47/357 25 → 20 137, -173, -113, 126, 166 82.698

48/357 51 → -17 128, 82, 202, 94, 117, 96, -201, -78, 73.992

-201, -110

49/357 -31 → 42 -173, -114, 126 79.959

50/357 62 → -55 146, 115, 141, -127, -187, 103, -169, 65.207

-149, 76

51/357 64 → 52 168, -103 68.997

52/357 -9 → 29 150, 184, 150, 184, 150 82.669

53/357 -32 → 66 149, 169, -103, 138, 105, 141, 107 59.543

54/357 53 → 70 15.145

55/357 67 → -28 137, -172, -113, 126, 166, -161, 87, 47.404

-74, -151

56/357 -34 → 53 -83 44.730

57/357 70 → 27 83 24.420

58/357 68 → -33 191, 86, -104, 87, -73, -151 45.789

59/357 23 → 6 139, -174, 139 78.117

60/357 22 → 41 112 64.784

61/357 32 → 62 146, 115, 141, -127, -135 63.115

62/357 47 → -5 -112 71.205

63/357 27 → 55 103, -169, -149 67.733

64/357 44 → 26 -120 82.181

65/357 -25 → 45 120 81.288

66/357 1 → 2 136, 71, 136, 97, 143 57.850

67/357 43 → 52 -85, 180, 64, 168, -103 39.042

68/357 69 → 61 -84, 155, 79, -144, -81, 93, -72 17.860

69/357 40 → -38 2.926

70/357 -12 → 55 3.398

71/357 1 → 71 136 46.189

72/357 -36 → 60 147 64.695

73/357 67 → 74 2.708

74/357 61 → 32 -72, -66, -107, -141, -105, -138, 103, 35.805

-169, -149

75/357 28 → 74 151 75.841

76/357 37 → 28 -91, -65, -188, 133, 165, 75, -171, 121, 56.644

-158, -196, 182, -90, 190, 195, -109,

-200, -160, -183

77/357 -38 → 48 85, 40, 118, -77, 119, -156, 118 13.993

78/357 -7 → 13 46 83.063

79/357 -12 → 39 2.055

80/357 53 → 27 -79, 100, 81, 95, 84, 70, 83 5.357

81/357 60 → -28 131, -102, -134, -130, -140, 88, 148, 46.339

67, 137, -172, -113, 126, 166, -161, 87,

-74, -151

82/357 -15 → 51 2.341

83/357 56 → 57 147 45.937

84/357 22 → -18 112, 41, 128, 82, 202, 78, 202, 94 15.567

85/357 -16 → 30 -162, -122, -186, -143, -125, -92, -164, 33.850

-192, 152, -177, 58, 177, -152, 192,

176, 92, 111, 122, 162, -157

86/357 57 → -33 131, 101, -134, 129, -140, -89, 148, 68, 47.546

191, 86, -104, 87, -73, -151

87/357 -29 → 35 1.921

88/357 33 → 73 151 65.194

89/357 55 → -32 -76, 149, 169, -103, 187, 127, -141, 32.668

-115, -146, -62, 135, 127, -141, -115,

-146

90/357 59 → 61 153, 69, -84, 155, 79, -144, -81, 93, 19.214

-72

91/357 34 → -23 0.454

92/357 67 → 24 0.363

93/357 -15 → 37 2.080

94/357 40 → 38 1.134

95/357 -10 → 24 0.599

96/357 43 → 13 1.273

97/357 50 → -43 135, 127, -141, -115, -52, 103, -168, 17.867

-64, -180, 85

98/357 62 → 3 0.501

99/357 38 → -5 0.545

100/357 17 → 4 110, 201, 78, 201, -96, -117, -94, -202, 18.191

-82, -128, -51, 108, -86

101/357 -16 → 60 1.115

102/357 -1 → 59 0.344

103/357 5 → 44 112, -47, 99, -124, 106 9.610

104/357 -6 → 62 0.503

105/357 57 → -56 0.828

106/357 12 → 65 0.809

107/357 49 → -21 0.871

108/357 -14 → 39 1.385

109/357 -3 → 24 0.507

110/357 5 → 22 1.414

111/357 12 → -11 1.843

112/357 19 → 4 1.093

113/357 -24 → 74 0.365

114/357 67 → 60 1.789

115/357 24 → 14 0.488

116/357 -8 → 24 0.600

117/357 6 → 58 0.409

118/357 -15 → 35 1.115

119/357 -4 → 27 0.960

120/357 67 → -6 1.048

121/357 39 → 70 0.146

122/357 -27 → 45 0.828

123/357 -11 → 26 1.261

124/357 8 → -7 0.998

125/357 59 → -34 1.226

126/357 -51 → 67 0.978

127/357 28 → 21 1.133

128/357 33 → -23 0.916

129/357 52 → -14 1.078

130/357 50 → -6 1.109

131/357 51 → 4 1.035

132/357 58 → -35 0.429

133/357 31 → 10 128, 82, 202, 94, 54, 110, 201, -96, 0.255

-98, 128, 82, 202, 78, 202, 78, 201,

-96, -98, 128, 82, 202, 94

134/357 -11 → 35 0.881

135/357 29 → -25 0.865

136/357 -39 → 40 0.526

137/357 -1 → 50 1.039

138/357 -49 → 68 -107, -141, -105, 56, 147, 57, 131, 101, 1.726

-134, 129, -140, -89, 148

139/357 44 → -25 1.080

140/357 38 → 44 0.305

141/357 74 → -7 0.951

142/357 52 → -23 0.915

143/357 64 → -50 168, -103, 52, 115, 141, -127, -135 3.323

144/357 -17 → 52 1.052

145/357 67 → -56 0.843

146/357 15 → 67 0.901

147/357 15 → 32 0.541

148/357 47 → 13 1.042

149/357 33 → 49 151, 73, -87, 104, -86, -191, -68, -148, 36.177

89, 140, -129, 134, -101, -131, -57,

-147, -56, 105, 141, 107

150/357 34 → 69 0.577

151/357 17 → 6 0.947

152/357 41 → -14 128, 82, 202, 78, 201, 78, 201, 78, 201, 0.214

78, 201, -96

153/357 38 → -32 -76, 149, 169, -103, 187, 127, -141, 0.901

-115, -146, -62, 135, 127, -141, -115,

-146

154/357 68 → -40 0.171

155/357 69 → -66 -84, 155, 79, -144, -81, 93, -72 1.964

156/357 -6 → 17 0.940

157/357 55 → -42 0.481

158/357 59 → 32 153, 69, -84, 155, 79, -144, -81, 93, 8.967

-72, 61, -72, -66, -107, -141, -105,

-138, 103, -169, -149

159/357 36 → 28 0.952

160/357 -58 → 74 0.279

161/357 64 → 24 0.200

162/357 24 → 12 0.266

163/357 -60 → 74 0.862

164/357 20 → 35 0.648

165/357 42 → 42 2.896

166/357 27 → 42 0.525

167/357 73 → -23 0.472

168/357 32 → -27 1.444

169/357 -4 → 70 0.209

170/357 56 → 68 147, 57, 131, 101, -134, 129, -140, -89, 2.848

148

171/357 -49 → 57 -107, -141, -105, 56, 147 9.067

172/357 -28 → 36 0.952

173/357 -45 → 70 0.183

174/357 -33 → 48 0.287

175/357 18 → 40 0.321

176/357 37 → 65 1.088

177/357 63 → -14 0.013

178/357 9 → -8 0.837

179/357 16 → 21 0.869

180/357 48 → -21 0.424

181/357 37 → -9 0.918

182/357 59 → 69 153 44.895

183/357 31 → 13 0.887

184/357 56 → 6 0.906

185/357 73 → -57 -87, 104, -86, -191, -68, -148, 89, 140, 15.026

-129, 134, -101, -131

186/357 24 → 54 0.475

187/357 62 → 62 0.870

188/357 69 → -53 -84, -95, -81, -100, 79 0.125

189/357 -24 → 59 0.060

190/357 53 → 38 0.049

191/357 48 → 44 0.240

192/357 49 → 23 0.811

193/357 2 → 58 0.242

194/357 19 → 65 0.730

195/357 44 → -41 0.573

196/357 74 → -60 -87, 161, -166, -126, 113, 172, -137, 13.035

-67, -148, -88, 140, 130, 134, 102, -131

197/357 -13 → 42 0.778

198/357 53 → -3 0.104

199/357 67 → 33 0.806

200/357 -1 → 48 0.305

201/357 13 → 10 110, 201, 78, 201, 78, 201, 78, 202, 94 0.239

202/357 62 → -24 0.089

203/357 3 → 37 0.853

204/357 37 → 48 0.243

205/357 -24 → 66 0.200

206/357 62 → -51 0.252

207/357 19 → 41 0.690

208/357 17 → 8 0.557

209/357 54 → 28 0.652

210/357 37 → 37 1.299

211/357 -4 → 24 0.192

212/357 -6 → 27 0.400

213/357 -13 → 56 0.777

214/357 -14 → 24 0.326

215/357 41 → 17 128, 82, 202, 94, 117, 96, -201, -78, 0.433

-201, -110

216/357 -36 → 67 147, 60, 131, -102, -134, -130, -140, 5.894

88, 148

217/357 37 → 26 0.804

218/357 41 → -3 128, 96, -201, -78, -202, -82, -128 0.056

219/357 11 → 37 -188, 133, 165, 75, -170, 132, 181, -90, 2.470

-189, -194, -109, -200, -160, -183,

-167, -175, -163

220/357 47 → -44 2.902

221/357 -37 → 65 163, 175, 167, 183, 160, 200, 109, 194, 1.307

189, 90, -181, -132, 171, -75, -165,

-133, 188

222/357 28 → 36 151, 74, -87, 161, -166, -126, 113, 172, 35.787

-137, -67, -148, -88, 140, 130, 134,

102, -131, -60, -147

223/357 71 → -24 0.435

224/357 10 → 28 133, 165, 75, -171, 121, -158, -196, 2.129

182, -90, 190, 195, -109, -200, -160,

-183

225/357 68 → 73 0.779

226/357 7 → 37 142, 165, 75, -171, 132, 181, -90, -189, 3.410

-194, -109, -200, -160, -183, -167,

-175, -163

227/357 3 → 16 0.855

228/357 16 → -7 163, 175, 167, 183, 160, 200, 109, -195, 3.354

189, 90, -182, 159, 170, -75

229/357 11 → 26 0.868

230/357 30 → -4 0.665

231/357 19 → 3 -154, 165, 75, -170, 132, 181, -90, 190, 2.513

195, -109, -199, -160, -183, -167, -175

232/357 11 → 59 0.212

233/357 -37 → 52 0.816

234/357 -14 → 15 128, 82, 202, 78, 202, 94 2.775

235/357 15 → 54 110, 201, -96, -98, 128, 82, 202, 94 0.022

236/357 3 → 54 128, 82, 202, 78, 202, 94 2.039

237/357 41 → 54 128, 82, 202, 78, 202, 94 2.729

238/357 41 → 12 128, 82, 202, 78, 201, -96 1.846

239/357 35 → 28 179, 165, 75, -116, -158, 181, -90, 190, 2.549

195, -109, -200, -160, -183

240/357 40 → 8 0.420

241/357 -15 → 19 0.751

242/357 -8 → 11 0.537

243/357 30 → -28 0.798

244/357 14 → 14 96, -201, -78, -202, -82, -128 2.888

245/357 47 → 29 0.824

246/357 48 → 4 0.346

247/357 58 → -17 0.220

248/357 -10 → 29 0.761

249/357 62 → -20 0.268

250/357 -12 → 43 0.536

251/357 33 → 4 0.845

252/357 -37 → 68 0.470

253/357 62 → -38 146, 115, 141, -127, -187, 103, -169, 0.700

-149, 76

254/357 18 → 57 0.589

255/357 40 → 73 0.142

256/357 65 → 48 0.385

257/357 -4 → 36 0.673

258/357 -28 → 71 0.609

259/357 32 → 24 0.118

260/357 40 → 56 0.251

261/357 28 → 50 1.195

262/357 66 → 24 0.147

263/357 -1 → 19 0.747

264/357 12 → 22 128, 96, -201, -78, -201, -110 4.134

265/357 62 → -12 0.522

266/357 -21 → 67 0.507

267/357 30 → 38 0.372

268/357 21 → -13 96, -201, -78, -201, -110 3.430

269/357 68 → -57 0.819

270/357 -5 → 67 0.777

271/357 54 → -14 110, 201, 78, 201, -96 2.843

272/357 54 → -2 110, 201, 78, 201, -96 1.318

273/357 54 → 15 110, 201, 78, 202, 94 1.652

274/357 54 → 54 110, 201, 78, 202, 94 2.067

275/357 57 → 58 0.129

276/357 21 → -15 96, -201, -78, -201, -110 2.541

277/357 -5 → 43 110, 201, 78, 201, -96 1.837

278/357 21 → -17 96, -201, -78, -201, -110 1.270

279/357 18 → -13 -94, -202, -78, -201, -110 2.817

280/357 17 → 54 110, 201, 78, 202, 94 1.486

281/357 17 → -2 110, 201, 78, 201, -96 2.425

282/357 62 → -21 0.197

283/357 62 → -14 0.148

284/357 62 → 43 0.110

285/357 61 → 41 0.214

286/357 60 → 28 0.621

287/357 48 → 48 -156, 118, -80, 119, -156, 118 1.872

288/357 -1 → 32 0.489

289/357 9 → 65 0.696

290/357 61 → 61 -72 10.099

291/357 33 → 21 0.593

292/357 34 → 29 0.167

293/357 55 → -24 -76 1.019

294/357 57 → 67 131, 101, -134, 129, -140, -89, 148 2.057

295/357 8 → 58 0.382

296/357 58 → 4 0.223

297/357 21 → 12 0.558

298/357 36 → 10 128, 82, 202, 94 2.433

299/357 36 → -20 128, 82, 202, 94 2.040

300/357 36 → 23 128, 82, 202, 94 2.076

301/357 -4 → 9 0.741

302/357 2 → 22 0.848

303/357 -23 → 38 0.431

304/357 47 → 4 0.651

305/357 7 → 19 142, 165, 75, -116, -158, 181, -90, 185, 2.726

-109, -200, -160, 178, 198, -145

306/357 74 → 67 0.702

307/357 67 → 40 0.192

308/357 63 → 4 0.011

309/357 16 → 18 0.812

310/357 43 → 44 0.325

311/357 31 → 4 0.674

312/357 58 → 43 0.373

313/357 44 → 65 0.544

314/357 63 → -43 0.009

315/357 19 → -12 0.727

316/357 26 → -16 0.730

317/357 58 → 18 177, -152, 192, 176, 92, 111, 122 0.492

318/357 -4 → 33 86, -108 2.555

319/357 -3 → 35 0.792

320/357 35 → -10 0.778

321/357 -4 → 63 0.012

322/357 26 → 44 99, -124, 106 2.424

323/357 41 → -39 0.815

324/357 3 → 22 128, 96, -201, -110 0.022

325/357 -8 → 16 -152, 192, 164, 92, 125, 143, 186, 122, 8.249

162

326/357 73 → -67 -87, 161, -166, -126, 113, 172, -137 9.577

327/357 70 → 40 0.083

328/357 50 → 1 0.842

329/357 -50 → 64 -85, 180 1.194

330/357 -50 → 52 0.764

331/357 43 → -6 -85, 180 5.051

332/357 11 → 38 0.316

333/357 -11 → 60 0.931

334/357 23 → 8 0.866

335/357 73 → 4 0.816

336/357 48 → 53 0.070

337/357 52 → 62 115, 141, -127, -135 0.593

338/357 67 → 20 137, -172, -113, 126, 166 2.314

339/357 67 → 42 137, -172, -113, 126 1.191

340/357 9 → 47 1.276

341/357 -20 → 31 -166, -126, 113, 173 0.533

342/357 20 → 63 0.010

343/357 58 → -49 0.331

344/357 16 → 60 0.777

345/357 7 → 28 75, -171, 121, -158, -196, 182, -90, 3.471

-189, 195, -109, -200, -160, -183

346/357 64 → -27 168, -103 1.197

347/357 49 → 63 0.007

348/357 40 → 1 0.376

349/357 -35 → 38 0.524

350/357 33 → 74 151 2.143

351/357 22 → -10 0.951

352/357 -26 → 45 120 4.362

353/357 56 → 60 147 1.258

354/357 49 → -7 -197, -178, 160, 200, 109, -185, 90, 1.055

-181, -132, 170, -75

355/357 56 → -33 147, 57, 131, 101, -134, 129, -140, -89, 45.071

148, 68, 191, 86, -104, 87, -73, -151

356/357 40 → 24 118, -80, 119, -156, 118 3.843

357/357 21 → 14 96, -201, -78, -201, -110, -54, -94, 21.163

-202, -78, -202, -82, -128

Applying bridges (2022-07-18 11:59:45)

--------------------------------------

Unicycler now applies to the graph in decreasing order of quality. This ensures that when multiple, contradictory bridges exist, the most supported option is used.

Bridge type Start → end Path Quality

simple long read -7 → 13 46 99.785

simple long read -9 → 29 150, 184, 150, 184, 150 99.123

simple long read -26 → -44 120 97.469

simple long read -25 → 45 120 96.533

simple long read 28 → 74 151 94.724

simple long read -6 → -23 -139, 174, -139 93.983

simple long read 9 → 39 91 93.449

miniasm 27 → 55 103, -169, -149 91.640

miniasm 25 → 20 137, -173, -113, 126, 166 90.860

miniasm -31 → 42 -173, -114, 126 90.834

miniasm -56 → 49 105, 141, 107 89.717

miniasm -40 → 38 -85 88.217

miniasm 26 → -45 99, -123, 106 87.489

miniasm -50 → -6 -85, 180 87.163

miniasm 52 → -50 115, 141, -127, -135 85.981

simple long read 5 → -47 112 85.355

miniasm -11 → 18 -152, 193, 164, 92, 111, 122 83.197

miniasm 64 → 52 168, -103 82.666

simple long read 65 → -37 91 82.538

long read 3 → 10 128, 82, 202, 94 82.440

long read 12 → 5 128, 96, -201, -78, -201, -110 82.411

miniasm 33 → 73 151 82.208

miniasm -62 → -32 135, 127, -141, -115, -146 82.196

long read 20 → -4 -94, -202, -82, -128, 98, 96, -201, -110 82.172

long read 2 → 17 96, -201, -78, -202, -78, -201, -110 82.166

miniasm 42 → 33 191, 86, -108 81.800

long read 31 → 23 128, 82, 202, 94 81.649

long read -1 → 15 128, 82, 202, 78, 202, 94 81.567

long read 48 → 24 -156, 118, -80, 119, -156, 118 81.451

long read 38 → -24 -76 80.578

long read 40 → 48 118, -77, 119, -156, 118 80.539

long read 36 → 35 128, 82, 202, 94 80.427

long read 15 → -14 110, 201, 78, 201, -96 80.420

miniasm -8 → -29 -152, 193, 164, 92, 111, 122, 162, -157 80.095

miniasm -68 → -57 -148, 89, 140, -129, 134, -101, -131 79.890

miniasm -67 → -60 -148, -88, 140, 130, 134, 102, -131 79.754

simple long read -36 → 60 147 79.477

long read 35 → 19 179, 165, 75, -116, -158, 181, -90, 185, 79.015

-109, -200, -160, 178, 198, -145

long read 19 → -8 -154, 165, 75, -170, 132, 181, -90, 78.871

-189, -194, -109, -199, -160, 178, 197,

-145

long read 16 → -10 163, 175, 167, 183, 160, 200, 109, -195, 78.860

189, 90, -182, 159, 170, -75, -165, -133

long read 30 → 37 179, 165, 75, -171, 132, 181, -90, -189, 78.817

-194, -109, -200, -160, -183, -167,

-175, -163

long read -12 → 22 96, -201, -78, -201, -110 78.400

long read 39 → -7 167, 183, 160, 200, 109, -185, 90, -181, 78.176

158, 116, -75, -165, -142

miniasm 73 → -68 -87, 104, -86, -191 77.573

long read -3 → 21 175, 167, 183, 160, 199, 109, -195, 77.436

-190, 90, -181, -132, 170, -75, -165,

154

simple long read 22 → 41 112 77.289

miniasm -4 → 51 86, -108 75.557

miniasm -47 → 44 99, -124, 106 75.287

long read 13 → 43 110, 201, 78, 201, -96 75.079

long read 51 → -17 128, 82, 202, 94, 117, 96, -201, -78, 73.992

-201, -110

miniasm 74 → -67 -87, 161, -166, -126, 113, 172, -137 72.434

long read 54 → -21 110, 201, 78, 201, -96 72.267

long read -28 → 65 183, 160, 200, 109, -195, -190, 90, 70.543

-182, 196, 158, -121, 171, -75, -165,

-133, 188

long read 49 → -11 -197, -178, 160, 200, 109, -185, 90, 69.241

-181, -132, 170, -75, -165, -133, 188

miniasm -32 → 66 149, 169, -103, 138, 105, 141, 107 69.210

miniasm 55 → -62 -76, 149, 169, -103, 187, 127, -141, 68.710

-115, -146

long read -14 → 54 128, 82, 202, 78, 202, 94 66.094

long read 41 → -18 128, 82, 202, 78, 202, 94 63.329

miniasm -69 → -59 -153 60.433

miniasm -59 → -34 60.286

simple long read -53 → 34 83 59.203

long read 1 → 71 136 57.850

long read 71 → 2 136, 97, 143 57.850

simple long read 56 → 57 147 57.688

miniasm 66 → -61 72 54.879

miniasm 53 → 70 46.969

long read 43 → 64 -85, 180 46.278

miniasm 58 → 30 177, -152, 192, 176, 92, 111, 122, 162, 35.737

-157

miniasm -16 → 58 -162, -122, -186, -143, -125, -92, -164, 34.231

-192, 152, -177

simple long read 70 → 27 83 33.966

long read 69 → 61 -84, 155, 79, -144, -81, 93, -72 19.214

Saving /data5/xuhao/3data/wang1_wang9/01.Cleandata/wang9/wang9_hy/004_bridges_applied.gfa

Bridged assembly graph (2022-07-18 11:59:46)

--------------------------------------------

The assembly is now mostly finished and no more structural changes will be made. Ideally the assembly graph should now have one contig per replicon and no erroneous contigs (i.e a complete assembly). If there are more contigs, then the assembly is not complete.

Saving /data5/xuhao/3data/wang1_wang9/01.Cleandata/wang9/wang9_hy/005_final_clean.gfa

Component Segments Links Length N50 Longest segment Status

total 4 4 5,610,616 5,232,712 5,232,712

1 1 1 5,232,712 5,232,712 5,232,712 complete

2 1 1 223,403 223,403 223,403 complete

3 1 1 149,719 149,719 149,719 complete

4 1 1 4,782 4,782 4,782 complete

Polishing assembly with Pilon (2022-07-18 11:59:46)

---------------------------------------------------

Unicycler now conducts multiple rounds of Pilon in an attempt to repair any remaining small-scale errors with the assembly.

Aligning reads to find appropriate insert size range...

Insert size 1st percentile: 223

Insert size 99th percentile: 574

Pilon polish round 1

Total number of changes: 73

Pilon polish round 2

Total number of changes: 26

Pilon polish round 3

Total number of changes: 25

Pilon polish round 4

Total number of changes: 8

Pilon polish round 5

Total number of changes: 5

Pilon polish round 6

No Pilon changes

Pilon polish round 7

No Pilon changes

Saving /data5/xuhao/3data/wang1_wang9/01.Cleandata/wang9/wang9_hy/006_polished.gfa

Rotating completed replicons (2022-07-18 14:14:47)

--------------------------------------------------

Any completed circular contigs (i.e. single contigs which have one link connecting end to start) can have their start position changed without altering the sequence. For consistency, Unicycler now searches for a starting gene (dnaA or repA) in each such contig, and if one is found, the contig is rotated to start with that gene on the forward strand.

Segment Length Depth Starting gene Position Strand Identity Coverage

1 5,232,708 1.00x UniRef90_Q8XBZ3 2,612,112 reverse 95.3% 100.0%

2 223,404 0.54x UniRef90_A0A109LD79 116,261 forward 97.8% 100.0%

3 149,719 1.84x UniRef90_J5VKS5 5,329 forward 99.7% 100.0%

4 4,782 20.79x none found

Saving /data5/xuhao/3data/wang1_wang9/01.Cleandata/wang9/wang9_hy/007_rotated.gfa

Assembly complete (2022-07-18 14:19:13)

---------------------------------------

Saving /data5/xuhao/3data/wang1_wang9/01.Cleandata/wang9/wang9_hy/assembly.gfa

Saving /data5/xuhao/3data/wang1_wang9/01.Cleandata/wang9/wang9_hy/assembly.fasta
