# Supplementary figures and images for: Coexistence of blaIMP-4, blaNDM-1 and blaOXA-1 in blaKPC-2-producing Citrobacter freundii of clinical origin in China
Source: Front Microbiol. 2023 Jun 12;14:1074612. doi: 10.3389/fmicb.2023.1074612 (PMC10291173; doi:10.3389/fmicb.2023.1074612)

*bla*IMP


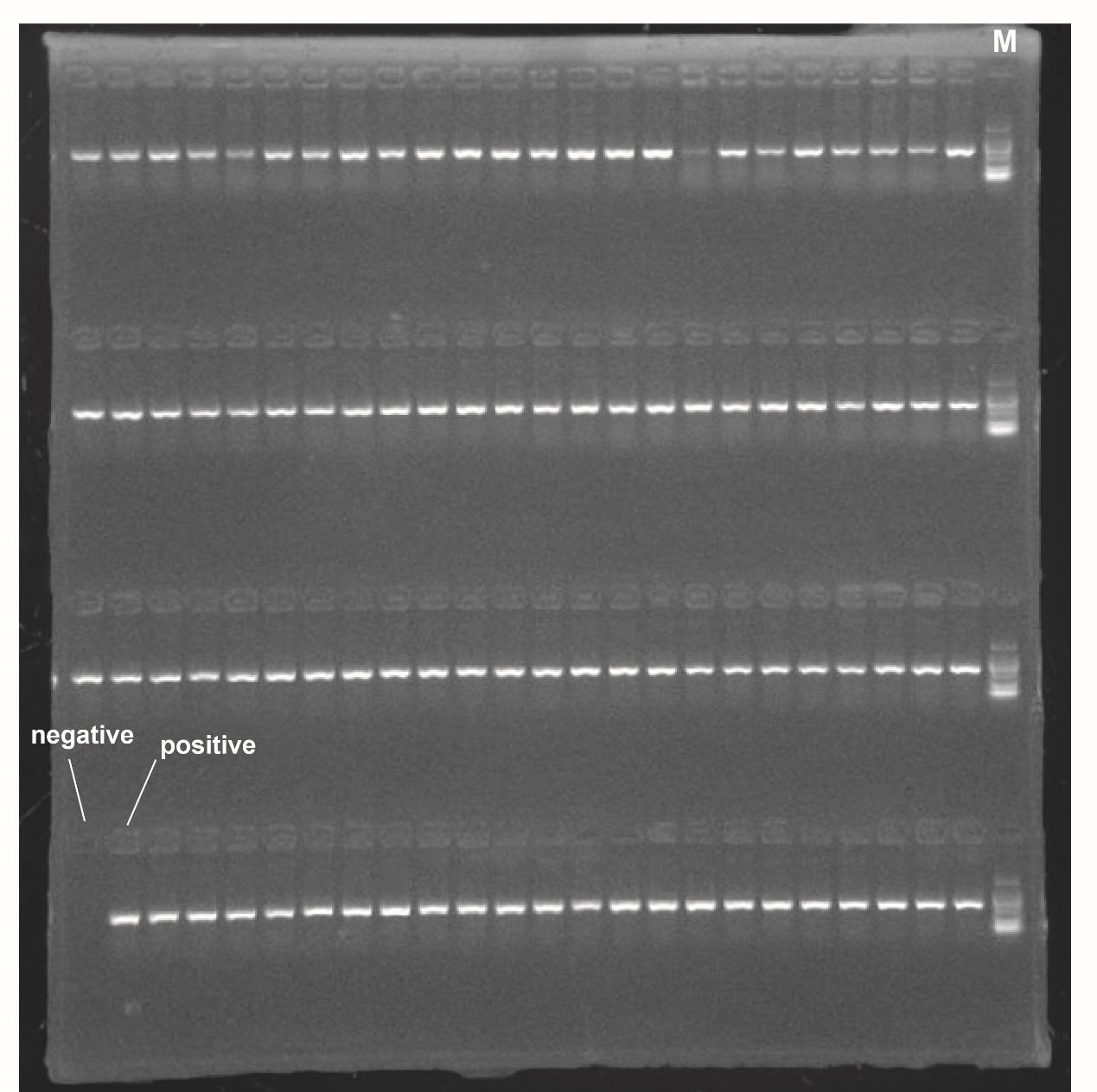


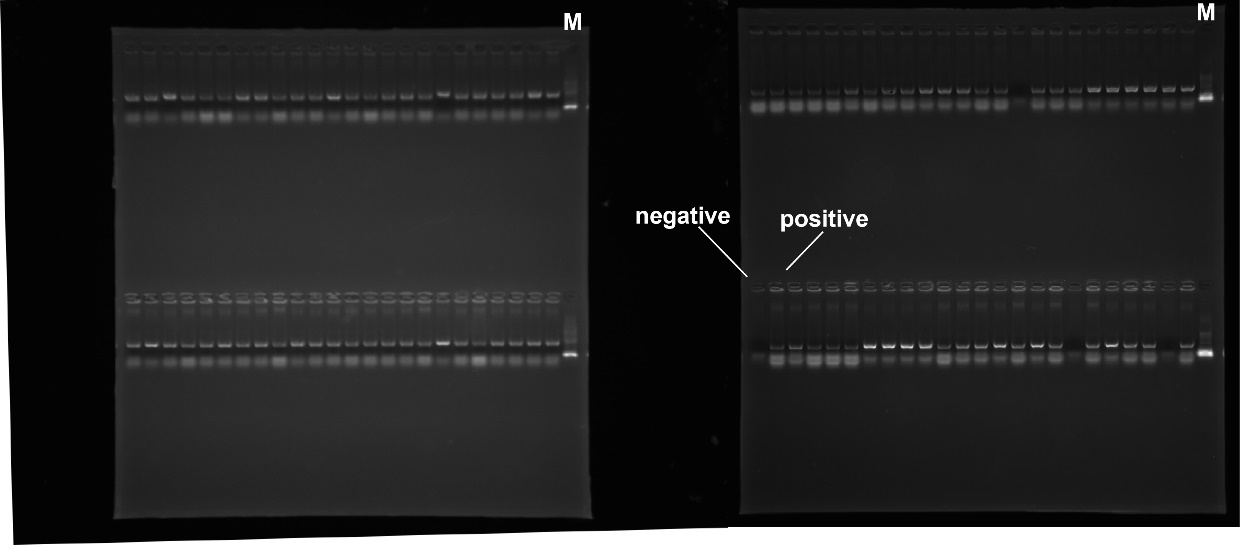


*bla*KPC


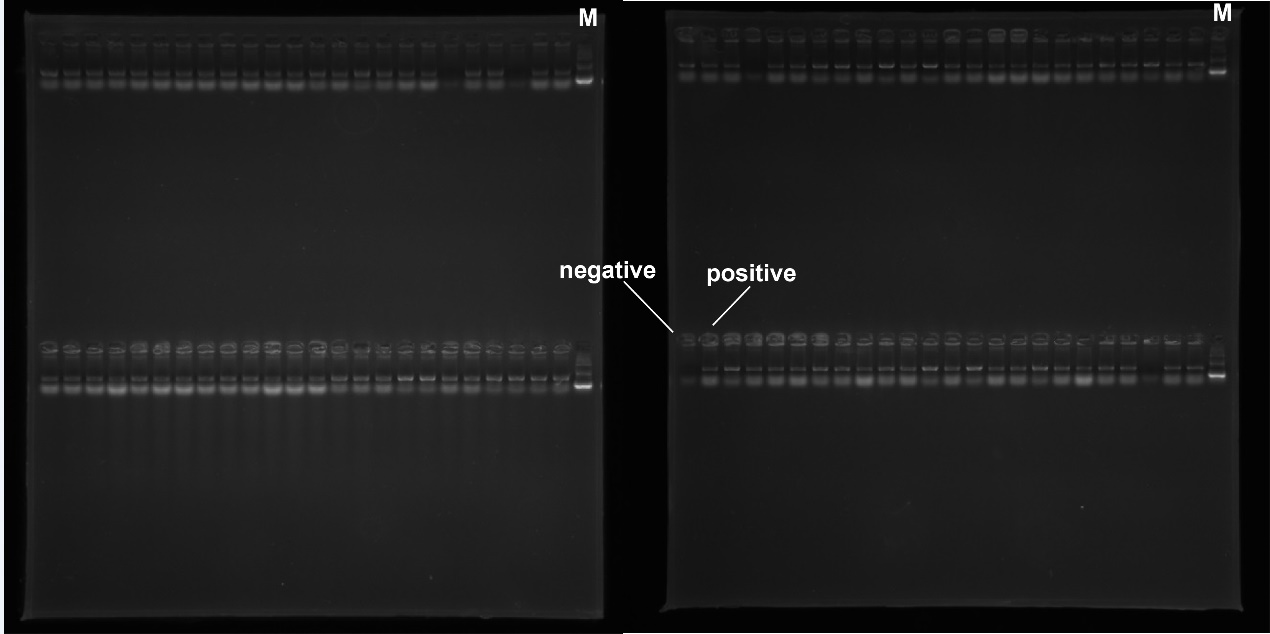


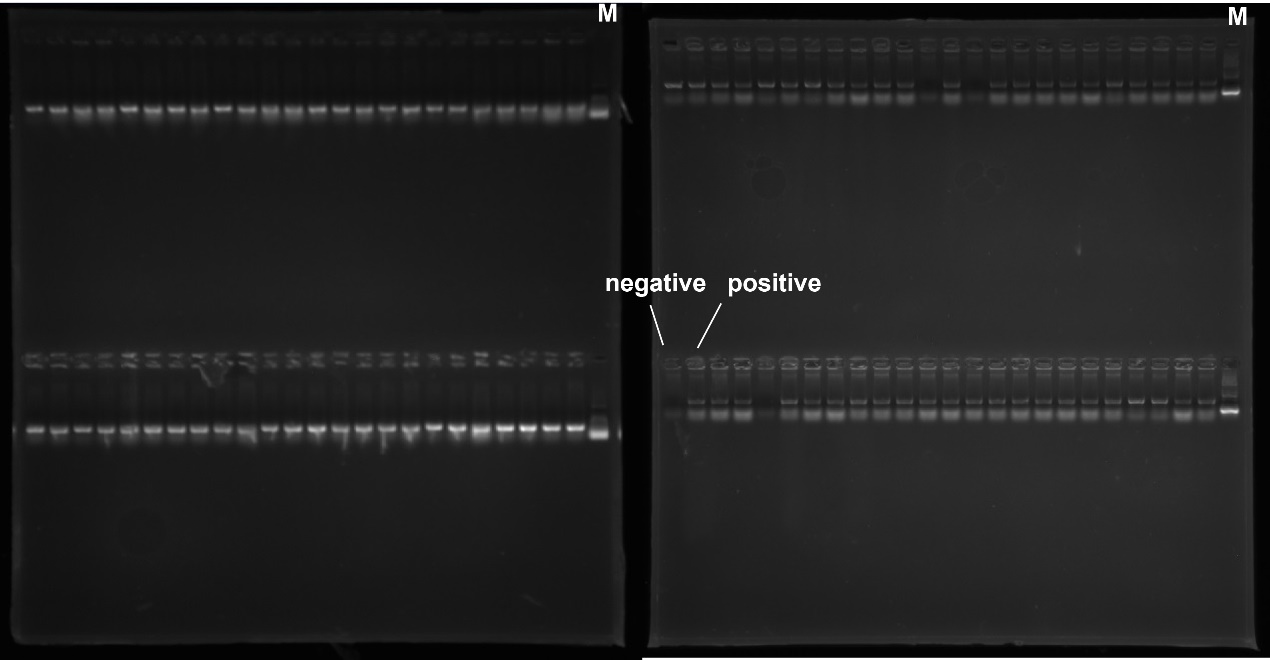

Supplement: Supplementary file 1 [file Data_Sheet_1.zip › Table S4.docx]

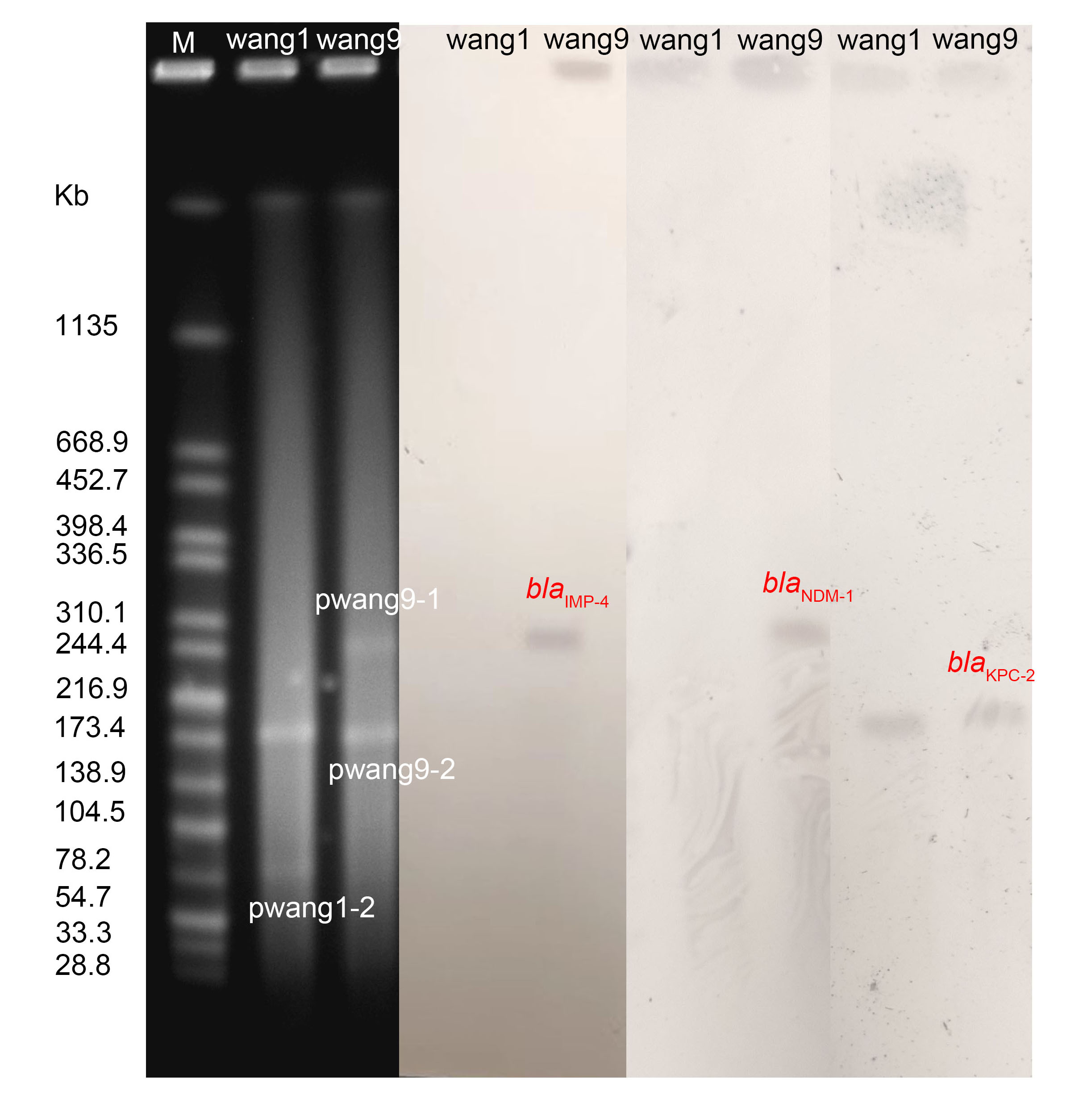

Supplement: Supplementary file 1 [file Data_Sheet_1.zip › Figure S3.jpg]

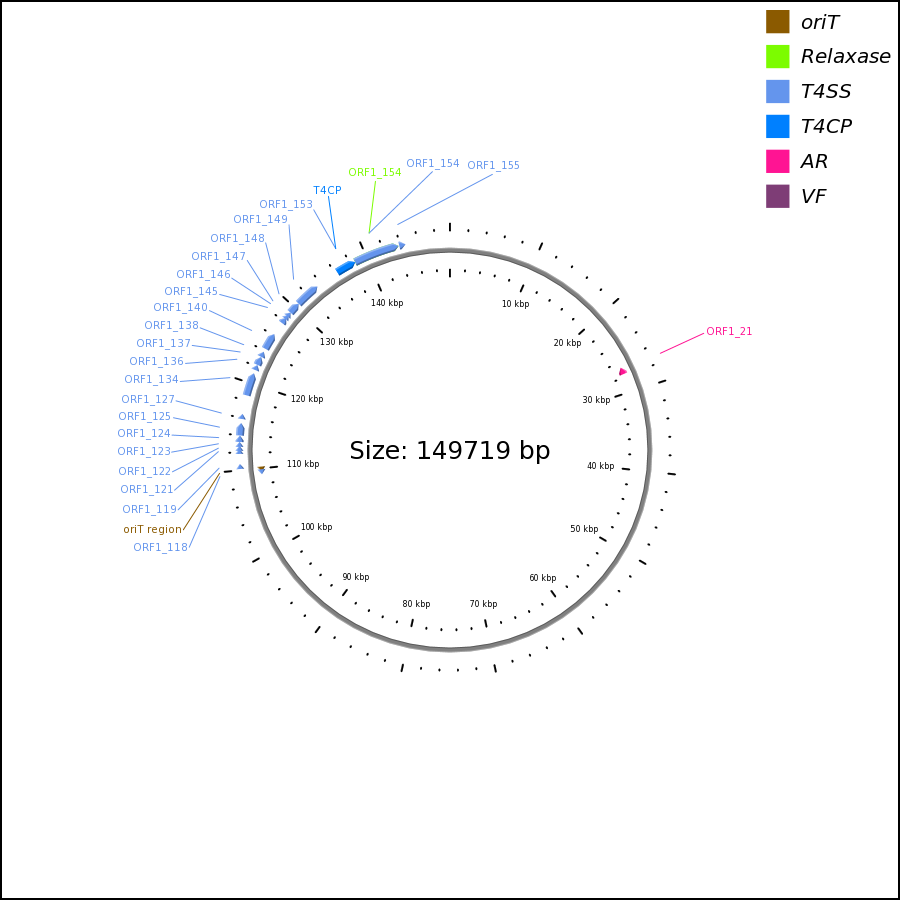

Supplement: Supplementary file 1 [file Data_Sheet_1.zip › Figure S4.jpg]

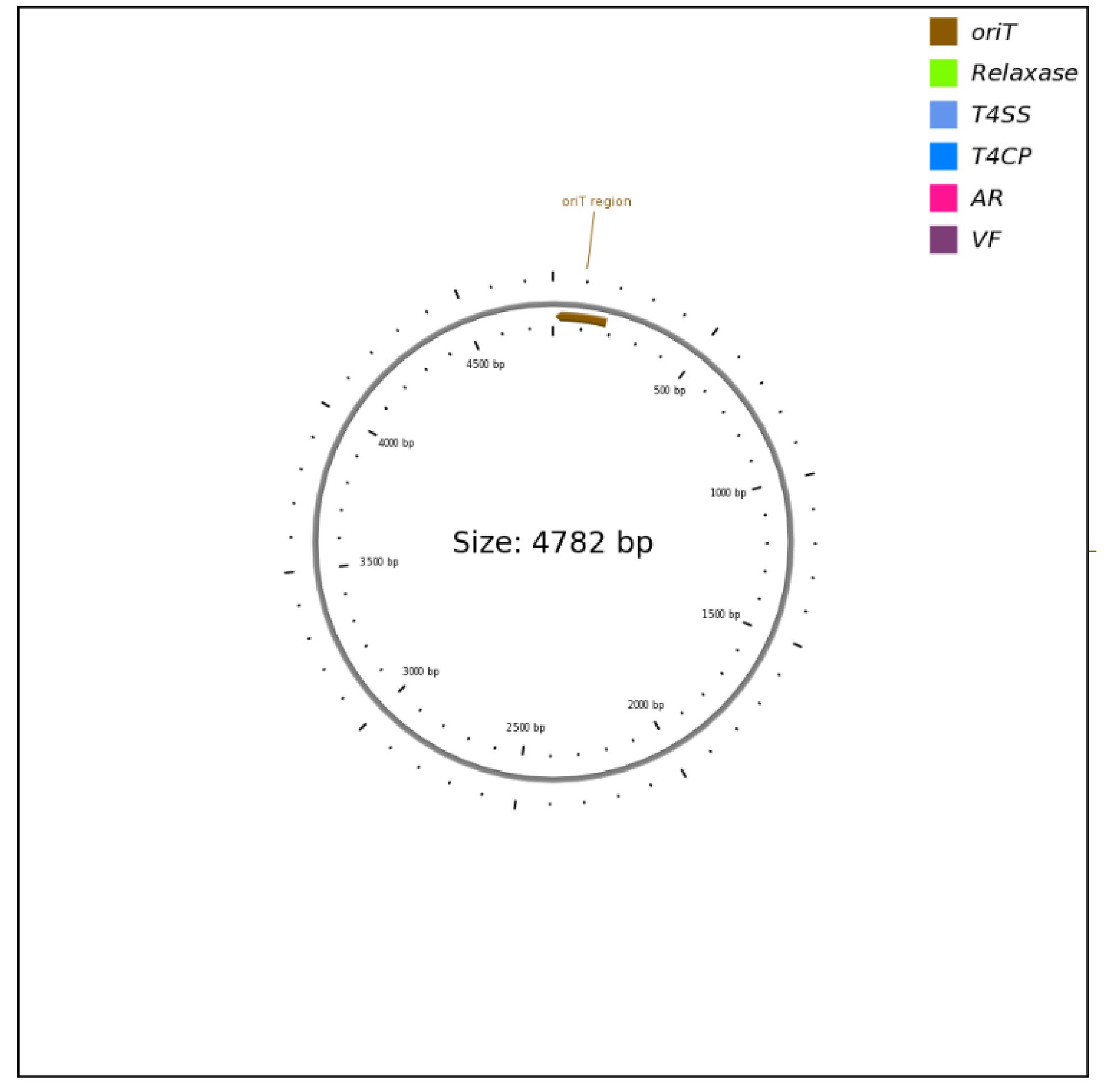

Supplement: Supplementary file 1 [file Data_Sheet_1.zip › Figure S5.jpg]

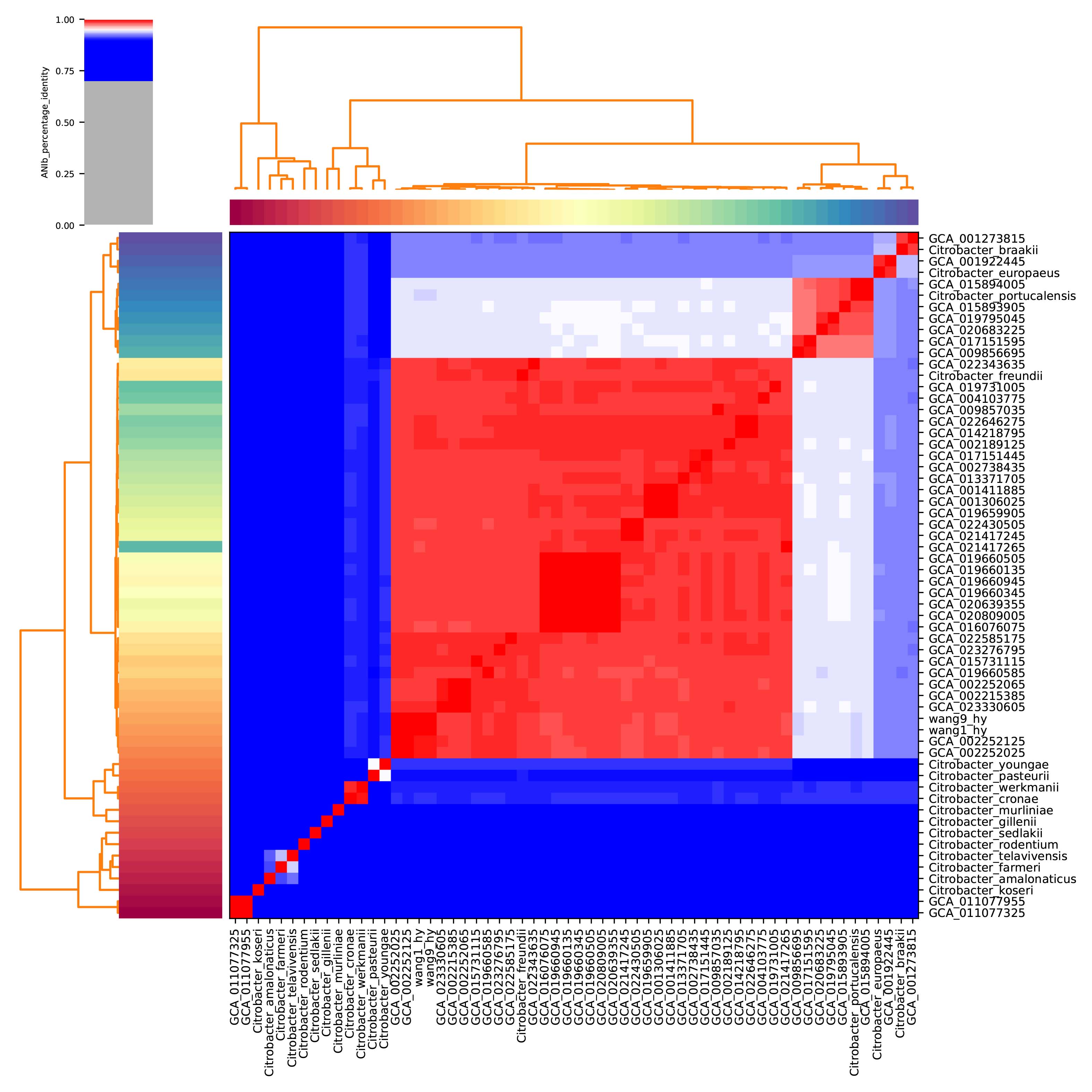

Supplement: Supplementary file 1 [file Data_Sheet_1.zip › Figure S6.jpg]

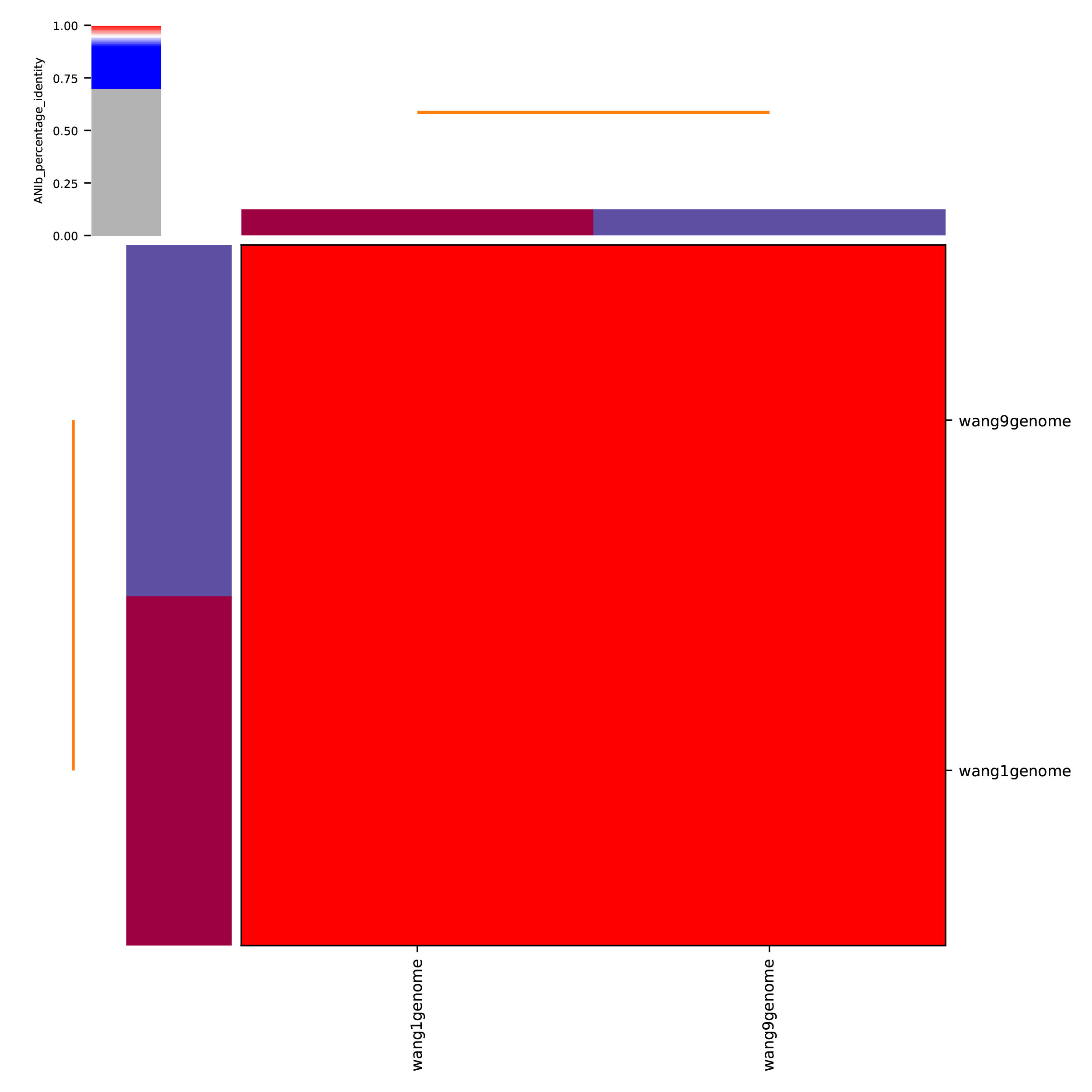

Supplement: Supplementary file 1 [file Data_Sheet_1.zip › Figure S7.jpg]

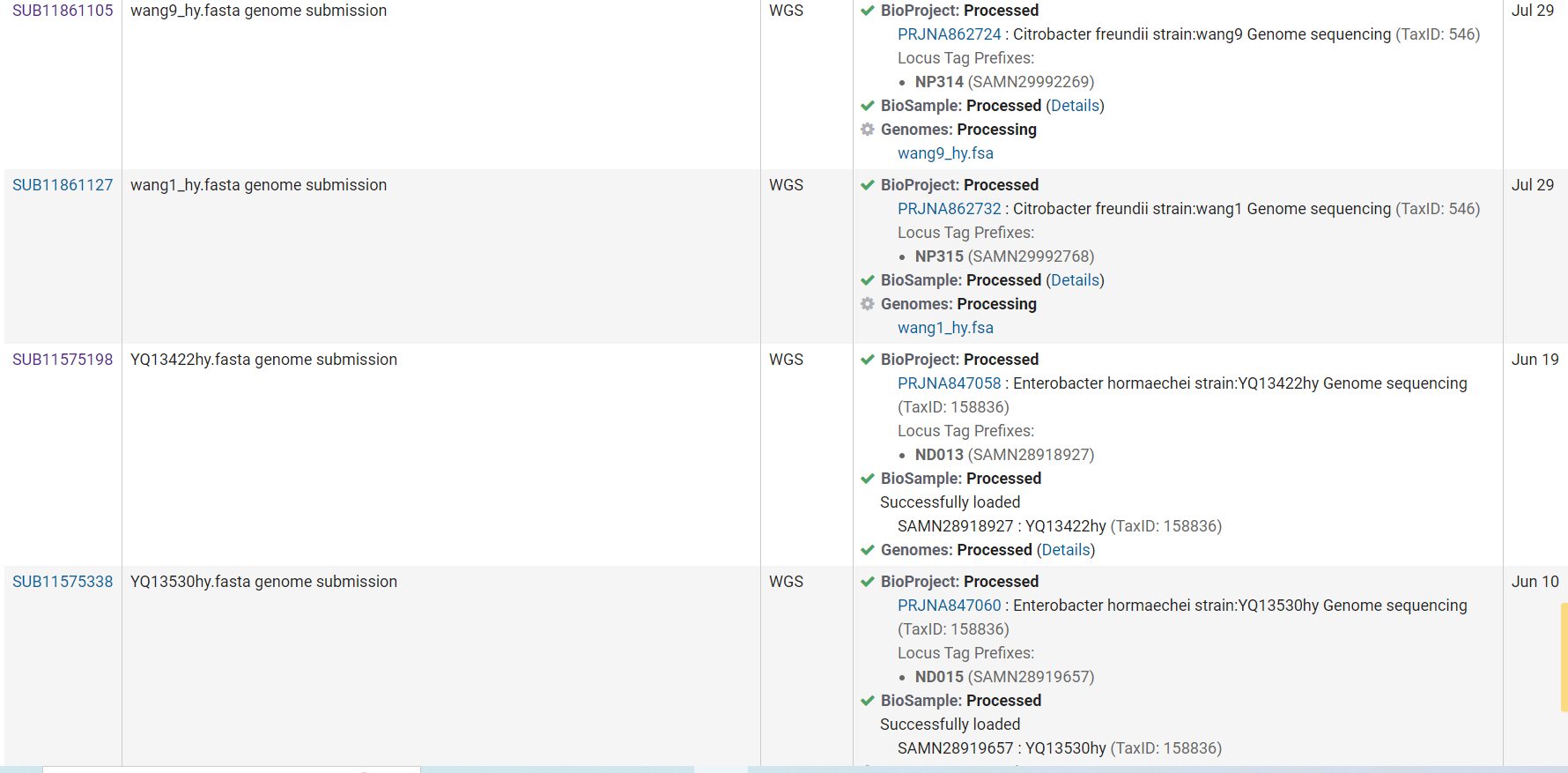

Supplement: Supplementary file 1 [file Data_Sheet_1.zip › Figure S8.jpg]
